# Irregular distribution of grid cell firing fields in rats exploring a 3D volumetric space

**DOI:** 10.1038/s41593-021-00907-4

**Published:** 2021-08-11

**Authors:** Roddy M. Grieves, Selim Jedidi-Ayoub, Karyna Mishchanchuk, Anyi Liu, Sophie Renaudineau, Éléonore Duvelle, Kate J. Jeffery

**Affiliations:** 1 Department of Experimental Psychology, Institute of Behavioural Neuroscience, University College London, London, UK; 2 Department of Psychological and Brain Sciences, Dartmouth College, Hanover, NH USA

**Keywords:** Cognitive neuroscience, Hippocampus, Spatial memory

## Abstract

We investigated how entorhinal grid cells encode volumetric space. On a horizontal surface, grid cells usually produce multiple, spatially focal, approximately circular firing fields that are evenly sized and spaced to form a regular, close-packed, hexagonal array. This spatial regularity has been suggested to underlie navigational computations. In three dimensions, theoretically the equivalent firing pattern would be a regular, hexagonal close packing of evenly sized spherical fields. In the present study, we report that, in rats foraging in a cubic lattice, grid cells maintained normal temporal firing characteristics and produced spatially stable firing fields. However, although most grid fields were ellipsoid, they were sparser, larger, more variably sized and irregularly arranged, even when only fields abutting the lower surface (equivalent to the floor) were considered. Thus, grid self-organization is shaped by the environment’s structure and/or movement affordances, and grids may not need to be regular to support spatial computations.

## Abstract (web summary)

Grieves et al. show that when rats explore a 3D space, grid cells in the entorhinal cortex exchange their usual spatially regular firing patterns for more irregular ones, suggesting that 3D space is mapped differently than previously thought.

## Main

Entorhinal grid cells tile a horizontal environment’s surface with a hexagonal-close-packed (HCP) array of approximately circular firing fields, the regular spacing of which is widely thought to provide a distance metric supporting the brain’s spatial cognitive map^1^. An unresolved but central question is whether this map can be three-dimensional (3D), as befits the behavioral ecology of most vertebrates. Hippocampal place cells, the core of the cognitive map in mammals^2^ and probably birds^3^ (but see ref. ^4^), form spatially defined firing fields in a volumetric environment in both bats^5,6^ and rats^7^, suggesting a capacity for the vertebrate brain to fully map volumetric space. We investigated whether this map could be founded on a regular 3D entorhinal grid.

**Fig. 1:**
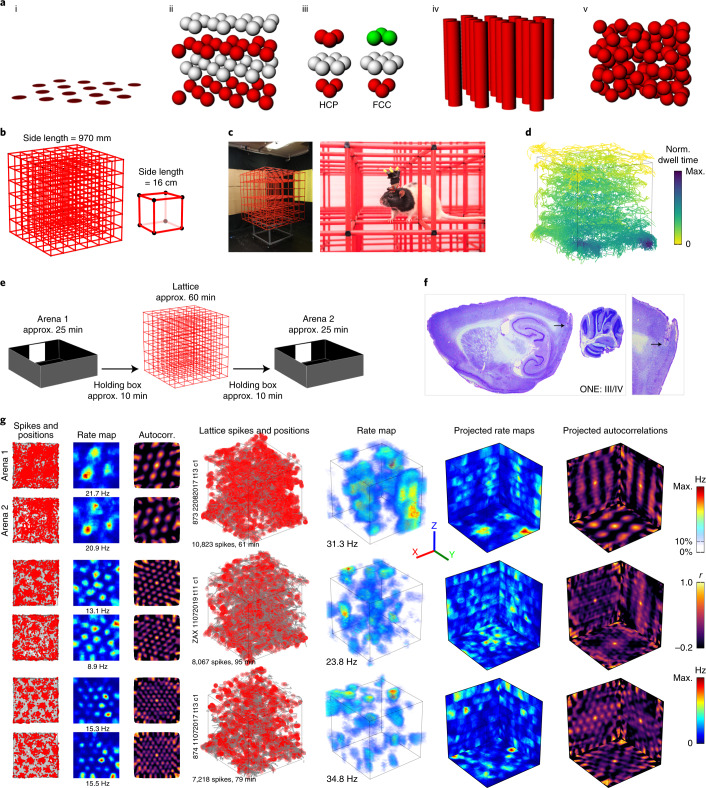
Grid cells produced firing fields in a 3D climbing lattice. **a**, Hypothetical grid field packings: standard horizontal hexagonal field configuration (i); exploded close-packed lattice, in this case HCP (layers color coded for clarity) (ii); units of the two optimal packings: HCP (left) alternates two layer-arrangements whereas FCC (right) has three (iii); columnar field configuration (iv); and random field configuration (v). **b**, Lattice maze schematic. **c**, Lattice maze photographs. See Extended Data Fig. 1a for arena photographs. **d**, Example coverage in a lattice session. Color denotes normalized (Norm.) dwell time in each region. **e**, Recording protocol. **f**, Example histology. Data for all animals can be seen in Supplementary Fig. 2. **g**, Three representative grid cells in the arenas (left) and lattice (right). Left–right: arena spike plots (gray shows coverage; red dots show spikes), arena rate maps, arena autocorrelations, volumetric spike plots, volumetric firing rate maps, rate maps as projected on to each of the three coordinate planes and projected autocorrelations. Color bars from top to bottom correspond to volumetric rate maps, autocorrelations and planar rate maps. All grid cells can be seen in Supplementary Fig. 1. Source data

Theoretical considerations suggest that in a volumetric space, the corresponding (and theoretically optimal) grid structure for 3D spatial mapping would be an HCP or face-centered-cubic (FCC) lattice of firing fields^8–13^ (Fig. 1a). However, previous studies on vertical surfaces found that grid fields formed vertical stripes^14^ or expanded blobs^15^ depending on the locomotor affordances (movement constraints) of the surface. In the present study, using wireless telemetry in rats exploring a cubic lattice maze (Fig. 1b–e), we investigated whether grid fields are indeed close packed (that is, optimally organized), randomly dispersed or somewhere in between (irregular with local order). We show that grid cells do stably express focal, 3D fields, but these are larger, more variably sized/shaped and more widely spaced than on a horizontal surface, and are distributed in a random pattern throughout the volume. We explore the implications of this for spatial computations.

## Results

**Extended Data Fig. 1:**
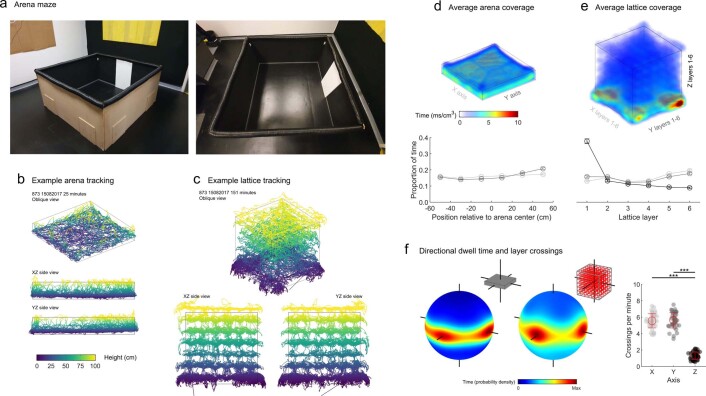
Animals explored the whole lattice maze with a bias for horizontal movements. (**a**) Side and top-down views of the arena. (**b**) Different views of the 3D positions for a representative arena session. (**c**) Different views of the 3D positions for a representative lattice maze session. (**d**) Top) averaged dwell time histogram for all arena sessions (n=42); Bottom) mean ± SEM proportion of time spent at different positions within the arena averaged across sessions. Rats explored the entire arena homogenously. (**e**) Top) averaged dwell time histogram for all lattice maze sessions (n=42); bottom) Mean ± SEM proportion of time spent in each layer of the maze, averaged across sessions. Rats explored the entire lattice homogenously in X and Y but with a strong vertical bias for the bottom layer. (**f**) Left) spherical heatmaps showing the time spent moving at every possible yaw × pitch angle in the arena and lattice (n=42 sessions). Inset schematics give the maze shape and the corresponding axes shown extending through the spheres. Right) Filled markers represent sessions (n=42), open circles denote mean, error bars denote SEM. As reported previously, rats were biased towards horizontal movements in both mazes and were far more likely to move parallel to the maze axes: the walls of the arena or the sides and bars of the lattice (*F*(2,123) = 417.2, *p* = 1.56 × 10^−55^, *η*^2^ = 0.872; one-way ANOVA).

We recorded medial entorhinal cortex (mEC) grid cells in seven rats freely foraging within a 3D climbing lattice maze^7,16–18^ (approximately 1 m^3^; Fig. 1b,c) and a standard horizontal arena (1.2 m^2^; Fig. 1e and Extended Data Fig. 1a). These recordings were made in an ‘A–B–A’ format: rats were recorded first in the horizontal arena (‘Arena 1’ trial) followed by the 3D lattice maze (‘Lattice’ trial), with a final session back in the original horizontal arena (‘Arena 2’ trial; Fig. 1e). As we used 3D tracking in all settings, grid fields were analyzed volumetrically even on the arena.

### Rats fully explored 3D, but anisotropically

Rats fully explored the environments, but in the lattice they spent more time in the bottom layer (Fig. 1d and Extended Data Fig. 1b–e). As we have found previously^18^, they mainly moved parallel to the maze boundaries and prioritized horizontal movements over vertical ones (Extended Data Fig. 1f; see ref. ^17^ for in-depth behavior analysis).

### Grid cells exhibited significant and stable spatial activity in the lattice

We recorded a total of 115 grid cells in layers II–IV of the mEC. For the main analysis, we selected only those cells that had stable grid firing in both arena sessions (*n* = 47; Fig. 1f,g, Supplementary Fig. 1 for all grid cells, Supplementary Video 1 for rotating plots, Supplementary Fig. 2 for all histology and Supplementary Table 1 for per-rat summary). Analyses for the remaining 68 cells (which were stable in one of the two arena sessions but not in both) are shown in Extended Data Fig. 5; including or omitting these cells did not change the results.

**Extended Data Fig. 2:**
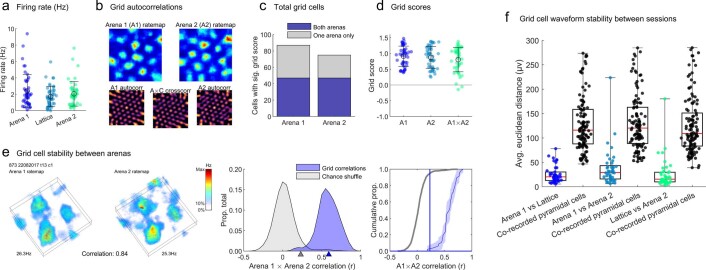
Grid cells were more stable than chance throughout recordings. For panels A, D, E & F: n=47 cells. For panels **a** & **d**: filled markers represent cells, open circles denote mean, error bars denote SEM. (**a**) Grid cell firing rates did not differ between the mazes (*F*(2,138) = 2.0, *p* = .135, *η*^2^ = 0.029, one-way ANOVA). (**b**) Example grid cell ratemaps and autocorrelations for the two arena sessions. In addition to ratemap autocorrelations we also calculated the cross-correlation between arenas 1 and 2. Only cells with a grid score exceeding the 95^th^ percentile of a shuffle in both arena sessions were analysed. (**c**) Total number of cells with a grid score exceeding the 95^th^ percentile of a shuffle in each arena session. The purple bar sections represent the cells categorised as grid cells and included in the main analyses, grey bars represent unstable cells that met our grid cell criteria in only one arena session. Main results were also replicated using only these unstable grid cells (Extended Data Fig. 5). (**d**) Grid scores of all grid cells calculated for both arenas and the cross-correlation between arena maps. These scores did not differ (*F*(2,138) = 1.1, *p* = .337, *η*^2^ = 0.016, one-way ANOVA). (**e**) Left) example firing of a grid cell in both arenas. Middle) to determine if grid cells were stable between arenas we correlated their arena firing rate maps (blue area; blue triangle denotes median) and compared this distribution to correlations between 5000 shuffled ratemaps (grey area; grey triangle denotes 95^th^ percentile). Right) cumulative density curves of the same distributions. Shaded areas denote 95% confidence intervals. The blue vertical line marks the 95^th^ percentile of the shuffle on the x-axis and the blue horizontal line (very close to the x-axis) marks the y-intercept of the observed correlation distribution with this. Grid cells were more stable than chance (shuffled arena correlations 95^th^ percentile: 0.23, grid cell median correlation: 0.59, D = 0.96, *p* < .001, two-sample Kolmogorov-Smirnov test). (**f**) The Euclidean distance between waveforms in different session pairs for all grid cells (Methods: *Waveform stability*). Red horizontal lines denote medians; black horizontal lines denote 1^st^ and 3^rd^ quantiles, error bars denote data range. Distances did not change across recordings (*F*(2,124) = 2.6, *p* = .0787; one-way ANOVA) suggesting grid cells were stably recorded throughout the experiment. Although distances were smallest when comparing the lattice to each arena (Group average Lattice vs Arena 1: 25.4, Lattice vs Arena 2: 25.5, Arena 1 vs Arena 2: 38.1) which is consistent with a gradual decrease in stability over time. In each case the distances between recording pairs were also significantly lower than chance which was estimated using pyramidal cell pairs co-recorded on the same tetrodes (*p* < .0001 in all cases, two-sample t-tests; black distributions).

The grid cells selected for the main analysis were stable throughout recording, as shown by similar firing rates throughout sessions, high grid scores (a measure of hexagonality) in the arena sessions and high cross-correlation between the two arena sessions. Spatial correlations were also high between the first and second arena trial maps, and cluster waveforms were stable throughout recording. These effects can be seen in Extended Data Fig. 2.

**Extended Data Fig. 3:**
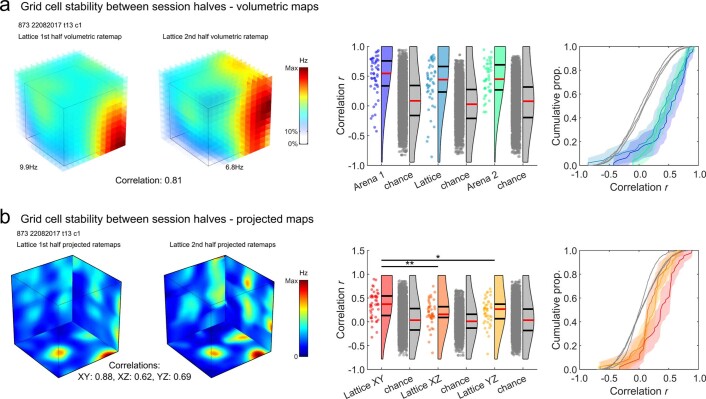
Grid cells were more stable than chance within sessions, especially in the XY plane. See Methods: *Spatial stability within sessions*. For panels **a** & **b**: n=47 cells. (**a**) Left) representative grid cell volumetric firing rate maps used for the volumetric correlation analysis; each represents one half of the same lattice session. Note that these maps have much larger voxels than ones used or shown elsewhere. Middle) raincloud plots showing the distribution of correlation values found for the arena and lattice sessions. Grey distributions represent correlations between random grid cells recorded in each maze. Red lines denote medians, black lines denote 1^st^ and 3^rd^ quantiles. Right) cumulative distribution functions of the same distributions. Shaded areas denote 95% confidence intervals. In all sessions grid cells were more stable than chance (*p* < .0001 in all cases; two-sample t-tests) and the mazes did not differ (*F*(2,137) = 1.6, *p* = .203, *η*^2^ = 0.023; one-way ANOVA). (**b**) Left) same cell as **a** but shown using projected maps used for planar correlations. Middle) raincloud plots as in **a**, showing the correlations found for each projected plane of the lattice for all grid cells. Right) cumulative distribution functions of these data. Shaded areas denote 95% confidence intervals. All projections were more stable than chance (*p* < .001 in all cases; two-sample t-tests) but horizontal (XY) projections yielded significantly higher correlations than vertical ones (*F*(2,135) = 5.8, *p* = .00377, *η*^2^ = 0.079; one-way ANOVA) which is consistent with previous findings in place cells.

**Extended Data Fig. 4:**
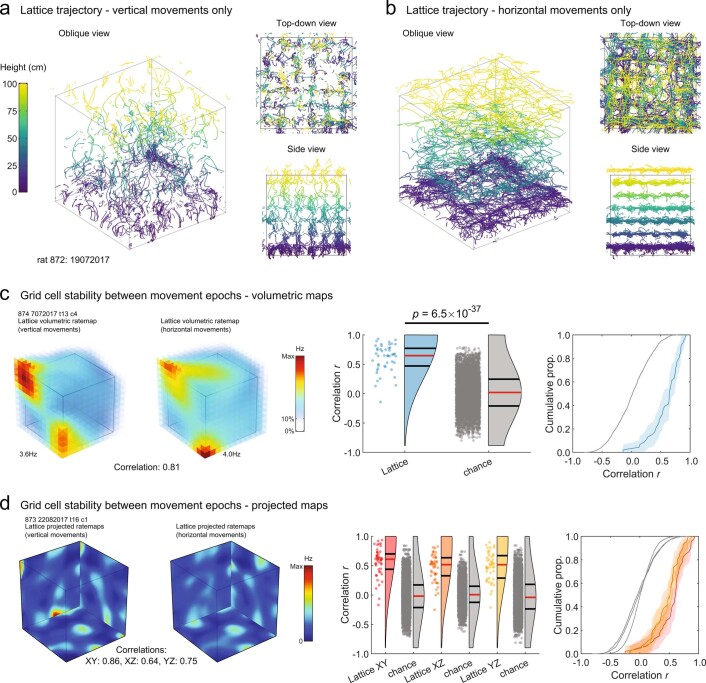
Grid cells did not remap according to movement direction. See Methods: *Stability between movement epochs*. For panels **c** & **d**: n=47 cells. (**a**) An example lattice trajectory filtered to show only vertical movements. (**b**) Same as **a** but for horizontal movements. (**c**) Left) example grid cell volumetric firing rate maps used for the movement direction analysis; each map represents the same grid cell activity filtered for vertical (left) or horizontal (right) movement epochs. Note that these maps have much larger voxels than ones used or shown elsewhere. Middle) raincloud plots showing the distribution of correlation values found for grid cells in the lattice (blue) and between random grid cells (grey). Red lines denote medians, black lines denote 1^st^ and 3^rd^ quantiles. Right) cumulative distribution functions of the same distributions. Shaded areas denote 95% confidence intervals. Grid cells were more stable than chance (*t*(4834) = 12.8, *p* = 6.49 × 10^−37^; two-sample t-test). (**d**) Left) example grid cell projected maps used for planar correlations. Middle) raincloud plots as in **a**, showing the correlations found for each projected plane of the lattice for all grid cells. Right) cumulative distribution functions of these data. Shaded areas denote 95% confidence intervals. All projections were more stable than chance (*p* < .0001 in all cases; two-sample t-tests) and they did not differ (*F*(2,135) = 2.1, *p* = .1249, *η*^2^ = 0.0303; one-way ANOVA).

**Extended Data Fig. 5:**
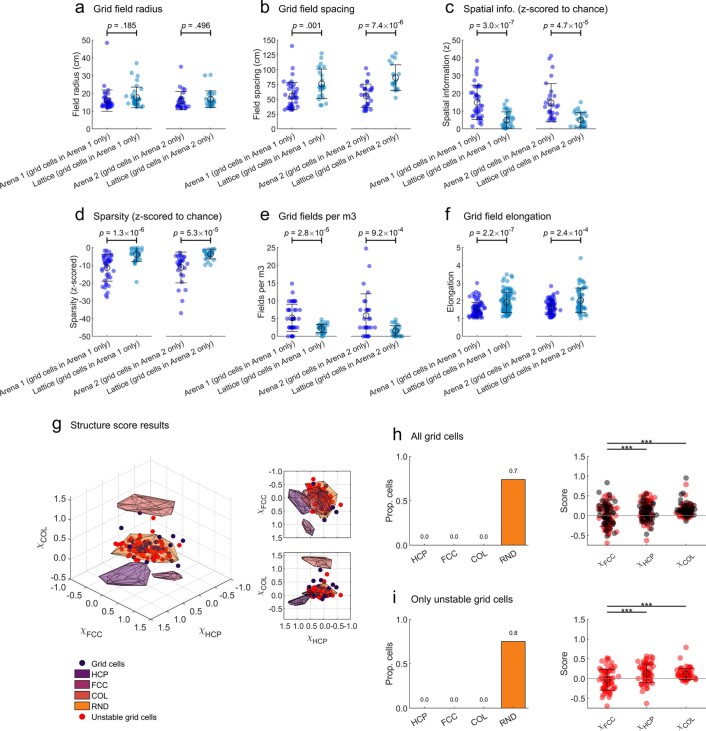
Main results were similar in unstable grid cells. These plots supplement main figures but focus on unstable grid cells (that is cells that met our grid cell criteria in only one arena session; grey areas in Extended Data Fig. 2c). For panels **a**-**f**, **h** (right) & **i** (right): open circles denote mean, error bars denote SEM and text gives the results of one-way ANOVAs. For multiple comparisons: *** = *p* < .001, ** = *p* < .01, ** = *p* < .05, all two-sided tests with Dunn-Sidak correction. (**a**) Supplement to Fig. 2g; Grid field radius was similar in the lattice and arena sessions. n=40, 35, 28 & 27 cells. (**b**) Supplement to Fig. 2i; grid spacing was significantly larger in the lattice. n=40, 25, 28 & 20 cells. (**c**) Supplement to Fig. 2b; Z-scored spatial information was higher than chance in all environments but reduced in the lattice. n=40, 36, 28 & 28 cells. (**d**) Z-scored sparsity was also lower than chance in all environments but was higher in the lattice. n=40, 36, 28 & 28 cells. (**e**) Supplement to Fig. 2f; grid cells exhibited significantly fewer fields per m^3^ in the lattice maze. n=76, 82, 68 & 74 cells. (**f**) Supplement to Fig. 4a; fields were significantly more elongated in the lattice. n=157, 233, 166 & 188 cells. (**g**) Supplement to Fig. 3a; structure scores (χFCC, χHCP and χCOL) for grid cells (n=47, black markers), unstable grid cells (n=68, red markers) and simulations (convex hulls shown as shaded polygons). (**h**) Left) Supplement to Fig. 3c; All grid cells (stable & unstable) categorized based on which convex hull they fell into. Right) configuration specific scores for stable (n=47, black markers) and unstable (n=68, red markers) grid cells. (**i**) Left) Supplement to Fig. 3c; unstable grid cells categorized based on which convex hull they fell into. Right) configuration specific scores for unstable grid cells (n=68) only.

In both the arena and lattice maze sessions, grid cell firing was spatially stable between session halves (halves versus shuffled: *P* < 0.001 in all cases, one-sample Student’s *t*-tests; no difference between mazes: *P* = 0.20, one-way analysis of variance (ANOVA); Extended Data Fig. 3), albeit with greater stability in the horizontal *XY* plane of the lattice than the vertical *XZ* or *YZ* planes (Extended Data Fig. 3b). Furthermore, grid cell activity was also spatially stable when comparing data separated into horizontal and vertical movement epochs (Extended Data Fig. 4), confirming that there was no movement-related change in firing (remapping) that might have obscured spatial firing patterns.

**Fig. 2:**
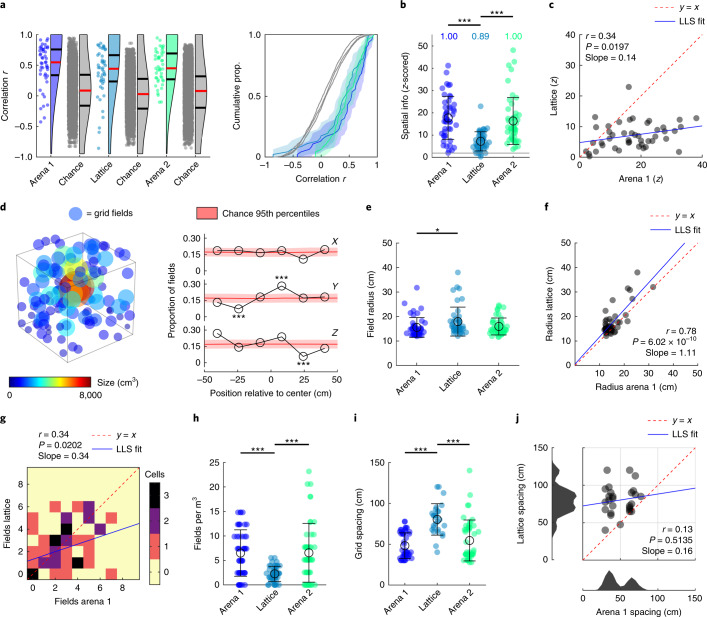
Grid cells mapped the lattice with large and widely spaced but stable fields. **a**, Grid cell firing was spatially correlated between session halves (red lines denote medians; black lines denote 1st and 3rd quantiles; see also Extended Data Fig. 4). Colored data points represent grid cells, gray represents shuffled values (*n* = 5,000). **b**, *Z*-scored spatial information was higher than chance in all environments but reduced in the lattice. Text gives the proportion of cells exceeding the shuffle 95th percentile (*z* = 1.96; gray line). **c**, Arena and lattice spatial information was significantly positively correlated (*n* = 46 cells). LLS, linear least squares line fit. **d**, Position and size of every grid field (left) and proportion of fields in every lattice layer (right; see also Supplementary Fig. 7b) (n = 133 fields). **e**, Grid field radius was significantly larger in the lattice than in the first arena. **f**, The size of grid fields in the arena and lattice was significantly positively correlated (*n* = 46 cells). **g**, The number of fields per grid cell in the arena and lattice was positively correlated. **h**, Grid cells exhibited significantly fewer fields per m^3^ in the lattice maze. **i**, Grid spacing was significantly larger in the lattice (*n* = 47, 43 and 47 cells for Arena 1, Lattice and Arena 2, respectively). **j**, Grid spacing (maximum 120 cm) in the arena and lattice was uncorrelated, and arena grid modules (bottom histogram) were disrupted in the lattice (*n* = 43 cells). Cells for which no lattice spacing could be estimated are not shown in **i** or **j**. **a**,**b**,**f**,**g**, *n* = 47, 46 and 47 cells. **b**,**f**,**g**,**i**, Markers represent cells, black open circles denote mean and error bars denote s.e.m. For multiple comparisons: ***P < 0.001, **P < 0.01, **P < 0.05, all two-sided tests with Dunn–Sidak correction. See Supplementary Fig. 4 for schematic and validation of procedures in **g**–**j**. Source data

Grid cell firing was more spatially clustered than chance even in the lattice (Fig. 2b), indicated by spatial information being consistently higher than spike-train-shuffled data (*P* < 0.05 in all cases, one-sample Student’s *t*-tests). However, spatial information was lower in the lattice than the arena and was closer to chance (*F*[2,137] = 20.3, *P* < 0.0001, *η*^2^ = 0.228, Lattice versus Arena 1 or Arena 2: *P* < 0.0001; all other: *P* > 0.05, one-way ANOVA; Fig. 2b). Although this suggests a disruption of grid cell spatial specificity, there was still a positive correlation between arena and lattice spatial information (Fig. 2c). Similar results were found when using sparsity as an alternative to spatial information content: this was lower than spike-train-shuffled data in all three mazes (Arena 1, Lattice and Arena 2 means: −12.4, −5.06 and −11.7; *P* < 0.05 in all cases, one-sample Student’s *t*-tests), but was closer to chance in the lattice (*F*[2,137] = 18.6, *P* < 0.0001, *η*^2^ = 0.213, Lattice versus Arena 1 or Arena 2: *P* < 0.0001; all other *P* > 0.05, one-way ANOVA).

### Grid fields were larger, more variable and more widely spaced in the lattice

Grid fields were observed throughout the lattice maze volume (Fig. 2d and Supplementary Fig. 3b). These tended, on average, to be larger in the lattice, as shown by the grid field radius (Supplementary Fig. 4), which was significantly larger in the lattice compared with the first arena session (*F*[2,131] = 3.7, *P* = 0.0284, *η*^2^ = 0.053, Arena 1 versus Lattice *P* = 0.0254; all other *P* > 0.05, one-way ANOVA; Fig. 2e). There was a significant positive within-cell correlation between field sizes in the arena and lattice (Fig. 2f).

Fields were also more variable in size: we assessed this by computing the coefficient of variation (CV), which is the standard deviation (s.d.) divided by the mean; this was significantly larger in the lattice, as shown by one-way ANOVA (Supplementary Fig. 3a; *F*[2,133] = 10.1, *P* = 0.0001, *η*^2^ = 0.1318). Post hoc tests also confirmed that the lattice differed significantly from each arena trial (*P* = 0.0004 and 0.0005, respectively). This suggests that fields did not conform to a grid structure.

The number of fields expressed in the arena and lattice was positively correlated (Fig. 2g), suggesting that the clumpiness of grid cell firing in two dimensions was still present to some extent in three dimensions. However, surprisingly, the numbers of fields exhibited in the arena and lattice did not differ (Arena 1: 2.6 ± 0.28; Lattice: 2.9 ± 0.29; Arena 2: 2.6 ± 0.34, mean ± s.e.m. fields; *F*[2,137] = 0.3, *P* = 0.72), meaning that there were significantly fewer grid fields per m^3^ in the lattice than in the arena (*F*[2,137] = 13.8, *P* < 0.0001, *η*^2^ = 0.167, Lattice versus Arena 1 or 2 *P* < 0.0001; all other *P* > 0.05, one-way ANOVA; Fig. 2f). This is consistent with findings from hippocampal place cells in the lattice maze, which also exhibited fewer fields per m^3^ than expected^7^, and from both place and grid cells on a vertical wall^15^.

Grid fields also exhibited significantly larger spacing in the lattice (*F*[2,118] = 22.0, *P* < 0.0001, *η*^2^ = 0.271, Lattice versus Arena 1 or 2 *P* < 0.0001; all other *P* > 0.05, one-way ANOVA; Fig. 2i) but spacing in the lattice was not correlated with arena spacing; instead, arena grid scale modules were disrupted in the lattice (Fig. 2j).

To summarize, grid cells in the lattice produced stable firing fields, but these were larger, more variably sized and more widely spaced.

### Grid fields did not form a close-packed configuration in the lattice

**Fig. 3:**
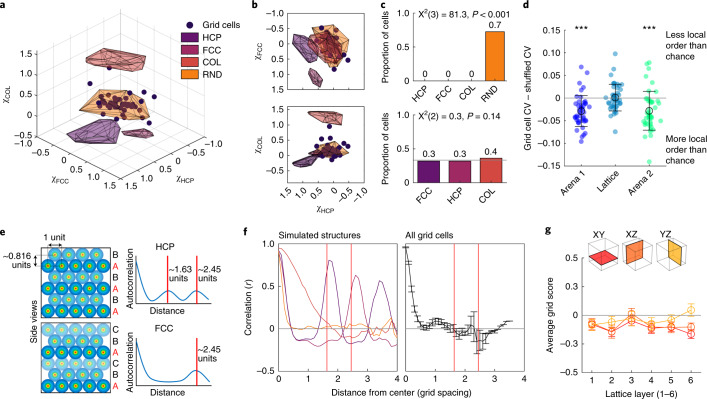
Grid fields were randomly distributed in the lattice. **a**, Structure scores (χFCC, χHCP and χCOL) for grid cells (markers) and simulations (simulated fields shown as shaded polygons; HCP, FCC, COL and RND are HPC, FCC, columnar and uniformly random field simulations respectively). **b**, The 2D side projections of **a** showing that grid cells overlapped the most with random configuration scores. **c**, Grid cells categorized based on which field region they fell into (top; 30% of cells are uncategorized) or which configuration score was maximal (bottom). **d**, Interfield distance CV for grid field distances in the arena and lattice compared with their respective shuffled data. Lattice data did not differ from spike-train shuffles (*n* = 45, 45 and 46 cells). Markers represent cells, open circles denote mean, and error bars denote s.e.m. **e**, Schematic of simulated HCP (top) and FCC (bottom) arrangements. Distance between repetitions of the same layer differ between layer repetitions in HCP (top) and FCC (bottom). **f**, The analysis shown in **e** finds the expected peak correlation patterns in simulated configurations (left; colors same as **a**) but not real grid cells (right; mean and s.e.m.). **g**, Grid cells exhibited low grid scores in all Cartesian coordinate planes of the lattice. Open circles denote average across cells and error bars denote s.e.m. **a**,**b**,**f**,**g**, *n* = 46 cells. For multiple comparisons: ***P = < 0.001, **P < 0.01, **P < 0.05; all two-sided tests with Dunn–Sidak correction. Source data

We next looked at the spatial pattern of the firing fields in the lattice maze. Previous theoretical and computational work suggests that the optimal packing of grid fields in 3D spaces would be an HCP or FCC configuration^8–13^. To test this, we first calculated a close-packed quality score (χ_CP_) that measures the presence of either close-packed structure. This score was significantly lower than would be expected if an FCC or HCP arrangement was present and was instead consistent with a non-close-packed arrangement of fields such as columnar or random (COL or RND; Fig. 3a; *F*[4,442] = 1,612.2, *P* < 0.0001, *η*^2^ = 0.936; all groups differ: *P* < 0.001 except grid cells and random *P* > 0.05). Configuration-specific scores (for FCC, HCP and columns; Supplementary Figs. 5 and 6) were also all close to zero and significantly lower than simulated configurations (Fig. 3a–c and Supplementary Fig. 7). Simulated field configurations most closely matching the real data were uniformly random (Fig. 3c) or shuffled ones (Supplementary Fig. 7b).

We explored this further by computing the CV of the interfield distances: this should be low if interfield distances are uniform. In the arena, interfield distances had a low CV, which is expected in a regular grid pattern. However, CVs in the lattice maze were no different from chance (Fig. 3d). Together, these findings show that grid fields in the lattice were randomly dispersed rather than regularly structured.

In an alternative approach, we reasoned that if a cell expressed an FCC or HCP firing pattern in the lattice maze, its firing rate map should be periodically self-similar (that is, correlate highly with itself), peaking with vertical shifts at multiples of approximately 0.816*d*, where *d* is the cell’s grid spacing. Furthermore, the position of these self-similarity peaks would depend on the firing pattern (Fig. 3e). Although this was correctly detected in our simulations, grid cells did not show evidence of either pattern (Fig. 3f). In addition, grid scores were generally negative for all layers of the lattice (Fig. 3g). Last, we took every possible plane sliced through grid cell firing maps and calculated the maximum possible grid score among them. These values still did not differ from chance in the lattice maze (Supplementary Fig. 7e), meaning that a hexagonal grid pattern could not be found at any arbitrary orientation.

In conclusion, a variety of analyses suggests that grid fields were randomly dispersed in the lattice.

### A minority of grid cells exhibited hexagonally arranged columnar fields

**Fig. 4:**
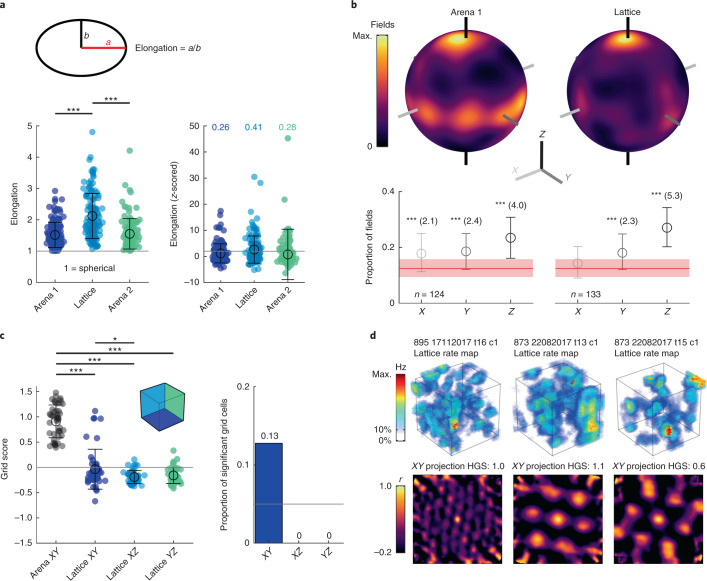
Grid fields in the lattice were vertically elongated and some formed hexagonal columns. **a**, Filled markers represent fields. Top: field elongation in the arena was calculated as the ratio of the largest (‘a’) and second largest (‘b’) axes. In the lattice, ‘b’ was instead calculated as the average of the two smallest axes. Bottom left: fields were significantly more elongated in the lattice. Right: *z*-scored elongation relative to 100 shuffles; text gives the proportion of fields exceeding the shuffle 95th percentile (that is, nonspherical; *z* = 1.96, gray line; *n* = 124, 133 and 120 fields for Arena 1, Lattice and Arena 2 trials respectively). **b**, Top: spherical heatmaps of the direction of all grid field principal axes (see Supplementary Fig. 9 for a schematic). Bottom: mean and 95% confidence interval of fields with a principal axis parallel to the *X*, *Y* or *Z* axis relative to chance (red area denotes 99th percentiles). Asterisks denote significant deviation; the numbers give effect size (Cohen’s *d*). **c**, Markers represent cells. Left: grid scores for all grid cells in the arena *XY* plane (*n* = 47) and each projected plane of the lattice maze (*n* = 46). Right: proportion of grid cells with a grid score exceeding the 95th percentile of a chance distribution in each lattice plane. **d**, Examples of significant *XY* grid cells recorded from two rats. Top: volumetric firing rate map. Bottom: autocorrelation of the *XY* projected firing rate map. See Extended Data Fig. 6 for square grid scores and Extended Data Fig. 7 for further analyses on significant *XY* grid cells. **a**,**c**, open circles denote mean and error bars denote s.e.m. For multiple comparisons: ***P < 0.001, **P < 0.01, **P < 0.05; all two-sided tests with Dunn–Sidak correction. Source data

Grid fields were slightly but significantly more elongated in the lattice than in the arena (Fig. 4a; *F*[2,374] = 48.2, *P* < 0.0001, *η*^2^ = 0.205, Lattice versus Arena 1 or Arena 2: *P* < 0.0001; all other *P* > 0.05, one-way ANOVA) and this elongation was mainly along the vertical axis (Fig. 4b). After correcting for this field anisotropy, grid cells still exhibited a random field configuration (Supplementary Fig. 8a,b), confirming that field shape did not obscure our original field configuration analysis.

Consistent with our previous results, when lattice maze activity was projected on to the bounding (coordinate) planes, the resulting grid scores were significantly lower in all three projections than the arena plane (Fig. 4c; *F*[3,181] = 169.6, *P* < 0.0001, *η*^2^ = 0.738, all lattice projections versus arena *P* < 0.0001; lattice *XY* versus *XZ*, *P* = 0.0425; all other *P* > 0.05). However, a small number of cells exhibited higher grid scores than expected by chance when projected on to the equivalent lattice *XY* (horizontal) plane (12.8%; Fig. 4c,d). These cells were recorded in two different rats across multiple sessions and tetrodes (Fig. 4d). These high *XY* grid scores in the lattice were associated with larger-scale grid cells (Extended Data Fig. 7a), but not with differences in theta modulation, animal behavior or mEC layer (Extended Data Fig. 7b–d). This might reflect animal-specific variation in vertical odometry as reduced vertical odometry has been seen in other settings previously^14,15^.

**Extended Data Fig. 6:**
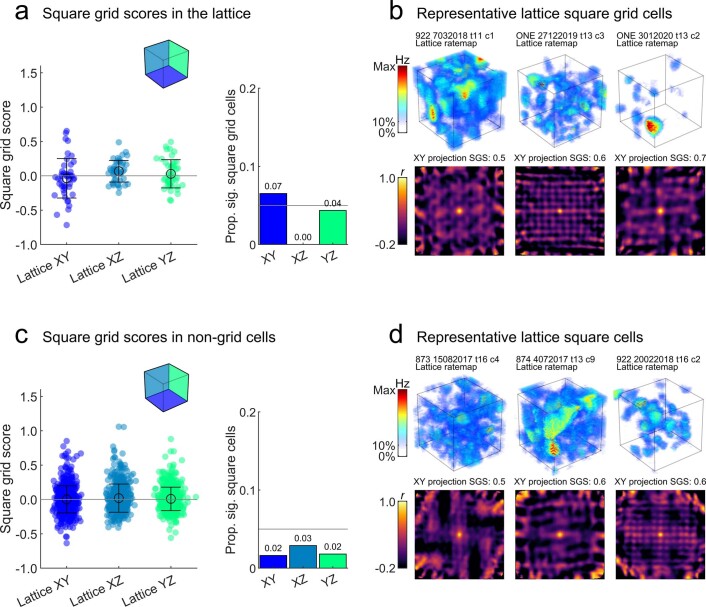
Weak evidence of square pattern activity in grid cells and non-grid cells. For panels **a** & **c**: Filled markers represent cells, open circles denote mean, error bars denote SEM. (**a**) Left) square grid scores for all grid cells (n=46) in each projected plane of the lattice maze. Square grid scores are low in each of the lattice planes and these did not differ (*F*(2,135) = 2.5, *p* = .0872, *η*^2^ = 0.036, one-way ANOVA). Right) proportion of grid cells with a square grid score exceeding the 95th percentile of a chance distribution in each lattice plane. A small number of cells exhibited a significant square firing pattern when projected onto the XY and YZ planes. Grey line shows the value that would be expected by chance (5%). (**b**) Three examples of these significant XY square grid cells. Top row shows the volumetric firing rate map, text gives the rat number, date, tetrode and cluster. Bottom row shows the autocorrelation of the XY projected firing rate map, text gives the square grid score (SGS) of the autocorrelation. (**c**-**d**) same as **a**-**b** but for all non-grid cells (n=550, 551 & 550). Square grid scores were again low and did not differ between projected planes (*F*(2,1648) = 1.2, *p* = .2975, *η*^2^ = 0.0015, one-way ANOVA). No plane exhibited more square grid cells than would be expected by chance (5%, grey line).

Evidence for square firing patterns^19^ was also observed in some grid cells but the overall proportion was close to that expected by chance (6.5% for *XY* plane; Extended Data Fig. 6a,b) and their square grid scores were similar to those found for nongrid cells (Extended Data Fig. 6c,d).

## Discussion

We found that rat grid cell firing fields filled a volumetric environment but did not make a regular (HCP or FCC) pattern^11,20^. They instead formed irregular configurations of enlarged, slightly vertically elongated, variably sized and more widely spaced fields. This irregularity is surprising given the regular pattern the cells create on horizontal surfaces but is consistent with computational models that predict local order in the absence of a regular close-packed structure^12,19,21,22^. It is also consistent with recent experimental results on a 2D vertical wall^15^.

We looked to see whether there might be hidden order in the form of a preserved local interfield distance, as suggested by a preliminary report in bats^23^, but found that distances were more consistent with a random arrangement. This difference in findings may be due to intrinsic species differences in physiology, but we think it more likely to be due to how movement patterns through the volumetric spaces can affect grid self-organization. Bats can fly directly in any direction, whereas the rats were largely constrained to paths aligned to the maze axes, and they prioritized horizontal movements. These movement constraints may have affected the initial conditions that influenced where the fields first coalesced^19^. Similar sensitivity to self-organization conditions occurs in other physical systems such as crystals. We can perhaps think of the 3D irregular grid field structure as analogous to an amorphous variant of the 2D, regular ‘crystalline’ one, similar to how amorphous silica is an irregular variant of quartz (regular) or glass (irregular with local order, similar to the bat findings). Such insights have important theoretical consequences. For example, our findings support the attractor models of grid formation, because the alternative oscillatory interference models would predict either a perfect lattice, if conditions allowed, or complete breakdown of the grids if they did not; they would not predict the randomly dispersed fields that we observed here. Our results also raise the possibility that, from a functional perspective, regularity and symmetry are not the critical features of grid cell firing, so much as the chunking of space that is a consistent feature of grid cell activity.

Theta, head direction, speed coding, spike dynamics and spatial information properties were largely preserved in the lattice maze (Extended Data Fig. 8 and Supplementary Figs. 10–12), suggesting that the lack of grid structure in three dimensions was not due to a disruption of these signals. However, grid field size and spacing increased whereas speed cell modulation decreased (Supplementary Fig. 10b) and grid spacing modules broke down—findings reminiscent of those seen in rats navigating a vertical surface^15^. These results could be explained by a partial failure to integrate the distance traveled through space, because this would result in breakdown of the grid firing pattern. Alternatively, if grid cells are updated using the same dual-axis rule that governs head direction cell activity in three dimensions^24,25^, then cumulative 3D movements could also disrupt overall grid activity^22^. If this were the case, then we might have expected to still see some hexagonal activity in the bottom layer of the maze, where rats mainly moved horizontally. However, it is possible that any tendency to regularity would have been disrupted by the development of nearby fields higher up in the maze.

**Extended Data Fig. 7:**
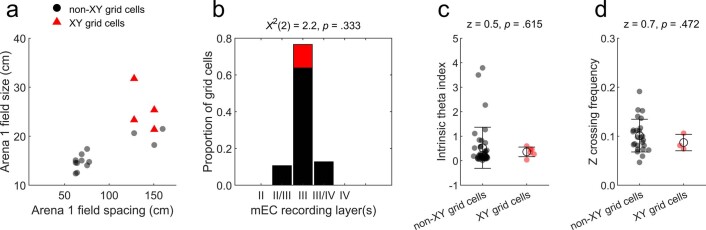
Cells with significant XY hexagonality in the lattice maze. For panels **c** & **d**: Filled markers represent cells, open circles denote mean, error bars denote SEM. Six grid cells (1 from one animal, 5 from another across a total of 3 sessions) showed a significant hexagonal grid score in the XY plane of the lattice maze. (**a**) Both grid spacing (z = −3.3, p < .001) and field size (z = 4.5, p < .0001; permutation tests: Methods: *Statistics and figures*) in the arena were significantly higher in the XY grid cells (n=6) than the remaining grid cells (n=41) suggesting that the hexagonality of large scale grid cells may be better preserved in 3D space. (**b**) The layer of the mEC the cells were recorded in was not related to XY hexagonality. Text gives the result of a two-sided Chi-square test of expected proportions which indicates that the layers where significant XY grid cells (red area) were found could be drawn by chance from the underlying distribution of grid cells (black area). (**c**-**d**) Theta modulation (Methods: *Spike phase and autocorrelation analyses*) and the proportion of layer crossings that were vertical in the lattice maze (Methods: *Behaviour and spherical heatmaps*) were also not related to XY hexagonality. Text gives the result of permutation tests (Methods: *Statistics and figures*). n=41 & 6 cells.

**Extended Data Fig. 8:**
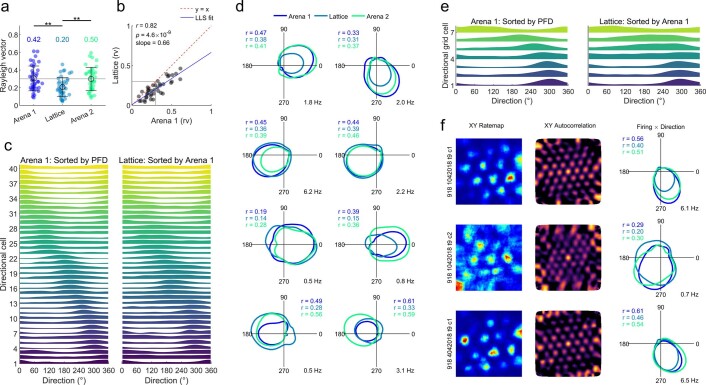
Directional modulation was preserved in the lattice. These analyses were conducted on head direction estimated from speed filtered displacement (Methods: Directional analyses and shuffles). (**a**) Markers represent cells, open circles denote mean, error bars denote SEM. Rayleigh vector lengths for all 40 directionally modulated cells (that is, any cells with a Rayleigh vector length exceeding the 95^th^ percentile of 100 spike train shuffles in both arena sessions, may include grid cells). Grey line denotes a commonly used arbitrary cut-off (0.3) and text gives the proportion of cells with a Rayleigh vector length greater than this. Vector lengths were lower in the lattice (*F*(2,117) = 7.1, *p* = .0013, *η*^2^ = 0.108, one-way ANOVA). (**b**) Vector lengths were correlated between Arena 1 and the Lattice suggesting preserved directional modulation in this maze. LLS: linear least squares line fit. Text gives the result of a Pearson linear correlation and the slope of the LLS. (**c**) Left) normalized Arena 1 tuning curves for all directionally modulated cells sorted by their preferred firing direction (PFD; peak firing). Right) normalized lattice tuning curves sorted by their Arena 1 PFD. The preserved diagonal ordering suggests that cells maintained the same allocentric firing directions in the lattice; the population correlation between arena and lattice tuning curves (*r* = 0.85) exceeded the 95^th^ percentile of 1000 shuffles (*r* = 0.33; z = 8.3, *p* < .0001) confirming this. (**d**) Overlaid arena and lattice polar tuning curves for 8 example directional cells. Coloured text gives the Rayleigh vector length for the corresponding tuning curves. (**e**) Same as **c** but for all conjunctive grid × direction cells (n = 7). As before, the correlation between arena and lattice tuning curves (*r* = 0.91) exceeded the 95^th^ percentile of 1000 shuffles (*r* = 0.55; z = 3.7, *p* = .0001) confirming that conjunctive grid cells maintained the same allocentric firing directions in the lattice. (**f**) Example conjunctive grid cells, one per row, ratemap, spatial autocorrelation and overlaid tuning curves as in D.

Despite the general breakdown of the grid firing pattern, we did observe planar (horizontally aligned) hexagonal activity in a minority of grid cells, recorded across two different animals. This activity approximated vertical columns and could be explained by preserved horizontal integration of distance traveled but poorer vertical processing in these animals^13,26^. Alternatively, this could be a feature of the self-organizing process: in a learning network model^19^ a behavioral bias for horizontal movements, which we did observe in our rats, sometimes led to the formation of hexagonally planar field arrangements rather than close-packed ones. Columnar fields have also been suggested as an efficient way for grid cells to represent higher-dimensional spaces, although in this case we would expect the fields of some cells to intersect rather than to all form vertical columns^27^. The sessions in which these columnar cells were recorded had no detectable peculiarity in terms of behavior or recording location (Extended Data Fig. 7). However, larger grid spacing was significantly associated with column-like activity, suggesting that the periodic activity of large-scale grid cells may be better preserved in 3D environments than small-scale cells. One possibility is that this effect is scale dependent: perhaps rats were not able to resolve their positions at a fine spatial resolution, resulting in expanded grid and place fields in the lattice maze, whereas distance estimation was preserved enough to maintain hexagonal grid firing at larger resolutions. Alternatively, columnar activity in the small-scale grid cells may have been disrupted by the structure of the maze. Further research is needed to disentangle these possibilities.

Given previous findings that place cells express spatially localized firing fields in volumetric space^7^ and that rats can navigate accurately in the lattice maze^17,18^, our results suggest that place cells and spatial mapping can perhaps function even when grid cell firing fields are irregularly distributed^18^. It may be that place cells do not require grid cell inputs for positioning when visual cues are available^28^, although future studies that simultaneously record place and grid cells in three dimensions, in the presence and absence of visual cues, are needed to fully explore this relationship. Alternatively, it may be that the information that grid cells supply to place cells is independent of the regularity of their fields.

In summary, our findings show that grid cells do generate firing fields that are distributed throughout a volumetric space, forming the potential substrate for a volumetric cognitive map such as the place cell map. However, they also show that the self-organizing process for grid cells is sensitive to environment type, with the result being variation in field size and regularity or irregularity of the grid depending on environment structure and/or movement affordances. These findings invite a reappraisal of the computational contributions that grid cells make to spatial mapping, because they suggest that any metric contribution of grid fields to spatial localization in 3D space (if there is one) must arise from the statistics of their dispersal rather than their precise arrangement.

## Methods

This experiment complied with the national (Animals (Scientific Procedures) Act, 1986, United Kingdom) and international (European Communities Council Directive of November 24, 1986 (86/609/EEC)) legislation governing the maintenance of laboratory animals and their use in scientific experiments. Experimental procedures were approved by the UK Home Office and ethical approval was granted through consultation with veterinary staff at University College London.

### Animals

Nine male Lister Hooded rats were used (only seven of these animals contributed grid cells; rats sourced from Charles River Laboratories), weighing approximately 400–450 g at the start of the experiment. No statistical methods were used to predetermine sample sizes but our sample sizes are similar to those reported in previous publications^7,15^. Before surgery all animals were housed for a minimum of 8 weeks in a large (2.15 × 1.55 × 2 m^3^) cage enclosure, lined on the inside with chicken wire. This was to provide the rats with sufficient 3D climbing experience. During this time, they were given unlimited access to a miniature version of the lattice maze (11 cm spacing rather than 16 cm for the recording lattice). The animals were housed individually in cages after surgery and given access to a hanging hammock or climbable nest box for continued 3D experience.

The animals were maintained under a 12 h light:dark cycle (light starting at 6am) and testing was performed during the light phase of this cycle. Throughout testing, rats were food restricted such that they maintained approximately 90% (and not less than 85%) of their free-feeding weight. A summary of the sessions and cells recorded from each rat can be seen in Supplementary Table 1.

### Electrodes and surgery

Axona (MDR-xx) microdrives were used. Drives supported four or eight tetrodes composed of four HML-coated, 17-µm diameter, 90% platinum/10% iridium wires (California Fine Wire), gold-plated (Non-Cyanide Gold Plating Solution, Neuralynx) to reduce their impedance to 180–300 kΩ measured at 1 kHz. Microdrives were implanted using standard stereotaxic procedures under isoflurane anesthesia^29^. Briefly, six support screws were inserted in the skull, electrodes were implanted after removal of dura, and the drive, skull and screw assembly was secured with an optional first layer of dental cement (Super-Bond C&B Metabond), followed by several layers of regular acrylic dental cement (Simplex Rapid Acrylic, Kemdent). Electrodes were implanted at a postero-anterior 8–11° slope (i.e., with the electrode tip pointing towards the animal’s nose), 1–1.5 mm below the dura, approximately 4.5 mm medial to the midline and as close as possible to the transverse sinus. See Supplementary Fig. 2 for histology results and a photo of an implanted drive.

### Apparatus

All experiments were conducted in the same room (3.2 × 2.1 × 2.2 m^3^) under moderately dimmed light conditions. Three of the room walls were covered with black material with large high-contrast cues on two of them (1.5 × 1.2 m^2^ cardboard sheet and a 1 × 1.7 m^2^ yellow plastic sheet). The fourth wall was covered with a white cotton sheet. The floor of the room was covered with black anti-static linoleum flooring. We used two pieces of experimental apparatus: a square open field environment (‘arena’) and a cubic lattice composed of horizontal and vertical climbing bars (‘lattice’).

The arena was a 1.2 × 1.2 m^2^ square high-walled wooden enclosure, composed of four 1.8 × 0.65 m^2^ matte black-painted walls (Extended Data Fig. 1a). The top edges of these walls were covered with large, corrugated tubing to prevent the rats from exploring this area. The bottom edge of this square was highlighted with a strip of 50% gray paint. One 0.45 × 0.65 m^2^ matte white wooden cue was affixed to one wall. Rats were recorded freely foraging in the arena for randomly dispersed, flavored puffed rice (CocoPops, Kelloggs).

The cubic lattice maze (Fig. 1a,b) was constructed from a children’s toy-set (miniQUADRO, Quadroplay). Hollow cubes were created by attaching red plastic tubes (length: 150 mm, diameter: 10 mm) using six- or four-way connectors (each 10-mm wide). These cubes were then assembled into a 6 × 6 × 6 cubic maze (0.97 × 0.97 × 0.97 m^3^). The maze was raised 0.45 m above the ground, initially on black metal stools but later on a narrow wooden frame. To encourage exploration, malt paste (GimCat Malt-Soft Paste, H. von Gimborn) was smeared onto the bars of the lattice by the experimenter. This paste was spread evenly throughout the maze, midway along the bars, equally between horizontal and vertical bars and reapplied every 15 min.

### Recording setup and procedure

Single unit activity was collected using a customized 64-channel recording system (Axona) running Axona dacqUSB software. The rat’s microdrive was connected to a wireless headstage (custom 64-channel, W-series, Triangle Biosystems Int.). Analog signals were transmitted to a wireless base station via dual receiver antennae situated approximately 1 m above the maze environments. Unfiltered signals were sampled at 50 kHz, amplified 100 times and transmitted at approximately 3.375 GHz (300 µW at 3 m). They were passed to an Axona preamplifier and amplified a further 100 times, and then to a system unit for single unit recording where the signal was bandpass (Butterworth) filtered between 300 and 7,000 Hz. Signals were digitized at 48 kHz and could be further amplified 10–40 times at the experimenter’s discretion. For local field potential (LFP) recording, a 4.8-kHz signal was saved as above, which was then bandpass filtered between 6 and 12 Hz (fourth-order Butterworth filter, MATLAB butter and filtfilt) for theta analyses described below. The position of the animal was recorded using five infrared sensitive CCTV cameras (Samsung SCB-5000P) tracking four wide-angle infrared light-emitting diodes (Osram Opto SFH 487P, 880 nm) fixed to the wireless headstage.

After recovery from surgery, rats were screened for single unit activity and the presence of theta oscillations once or twice a day, 5 d a week. Screening was performed in the open field apparatus, after which rats freely foraged on the lattice maze for the same duration to ensure equal novelty between the mazes. Once the presence of grid cells was confirmed, rats were recorded using the experimental procedure (Fig. 1e).

In these sessions, rats were recorded for a minimum of 18 min in the arena and until they had sufficiently explored the environment (mean ± s.d.: 27.8 ± 3.8 min). They were then allowed to rest and drink in an opaque, lidded box for approximately 10 min. During this time, the arena was dismantled and replaced with the lattice maze. Rats were then placed on the bottom layer of the lattice and left to forage in this environment for a minimum of 45 min and until they had sufficiently explored the environment (mean ± s.d.: 69.0 ± 22.8 min). Rats were returned to the opaque box as before and then recorded in the arena for a further minimum of 16 min and until they had sufficiently explored the environment (mean ± s.d.: 27.4 ± 9.9 min). During recordings, the experimenter monitored progress from a connected room, which housed the recording equipment and was separated from the experimental room by a black opaque curtain.

At the end of the recording session, the animals were removed from the apparatus and the electrodes were lowered by at least 20 µm to maximize the chance of recording from a different population of cells on the following day. Grid cells with similar fields seen on the same tetrodes on consecutive days were discarded; all analyzed grid cells can be seen in Supplementary Fig. 1. Rats were tested until grid cells were no longer observed and it was judged that the electrodes had left the desired layer (mean ± s.d.: 4.7 ± 2.7 sessions).

### Trajectory reconstruction

The rat’s position was tracked in real time at a 25-Hz sampling frequency using DacqTrack software (Axona). Position data were synchronized with neural data using a pulsed optic interface—each camera monitored a 1-Hz TTL (through the lens)-initiated light source, controlled by the recording system, which allowed accurate, offline synchronization. The onset of these light pulses was used to continually realign the position data using nearest neighbor interpolation (MATLAB interp1).

The rat’s 3D position was then reconstructed using the direct linear transform algorithm^30^, applied to the data from all five cameras, in pairs. Briefly, these cameras were first calibrated to reverse any distortion introduced by their optical elements (MATLAB estimateCameraParameters, undistortImage and undistortPoints). We then imaged the same checkerboard pattern with each camera and used its 3D pose to calculate the distance and orientation of each camera relative to it and thus to each other (MATLAB extrinsics and cameraMatrix). Using this information, we constructed a fundamental matrix. If *x* represents some points viewed by camera 1 and *x*′ represents the same points viewed by camera 2, the fundamental matrix, *F*, represents the relationship between points *x* and *x*′.

$$x_i^\prime Fx_i = 0.$$

This relationship can be used to triangulate any given pair of points imaged by two cameras into 3D space^30^. For each recording session we reconstructed the animal’s path using every possible pair of cameras (MATLAB triangulate) and we then combined these reconstructions into one single trajectory. This was achieved by taking the weighted mean of each point, where the weighting was the reliability of the point’s estimated location. Reliability was assessed using each point’s reprojection error; after triangulation, each point was projected back into both camera images and the reprojection error was then calculated as the distance between the original and reprojected position of the point.

In this setup, the rats need be viewed by only two cameras at any one time for a successful reconstruction, allowing for almost continuous tracking even in cluttered, complex environments such as the lattice maze. Our cameras were extremely stable; however, recalibrations were conducted once every 2–4 weeks to ensure continued reconstruction accuracy. For segments of missing tracking data, we simultaneously interpolated and smoothed the existing data using an unsupervised, robust, discretized, *n*-dimensional spline-smoothing algorithm (MATLAB smoothn^31,32^). Example 3D trajectories can be seen in Extended Data Fig. 1b,c.

### Behavior and spherical heatmaps

Using smoothed and interpolated 3D reconstructed position data we calculated the instantaneous 3D heading of the animal as the normalized change in position:

$$\hat u = \frac{{\vec u}}{{\left\| {\vec u} \right\|}}$$

where:

$$\vec u = \left( {{\Delta}_X\left( t \right),{\Delta}_Y\left( t \right),{\Delta}_Z\left( t \right)} \right)$$

and

$$\left\| {\vec u} \right\| = \sqrt {{\Delta}_X\left( t \right)^2 + {\Delta}_Y\left( t \right)^2 + {\Delta}_Z\left( t \right)^2}.$$

This gives a unit vector representing the animal’s heading at time *t*. For visualization we projected these vectors on to a unit sphere as described below (Grid field orientation).

Grobéty and Schenk^16^ and Jovalekic et al.^18^ previously reported, in lattice mazes similar to the one used here, that rats exhibited a strong bias for horizontal movements. To test this in our lattice maze, we compared the number of times each animal crossed from one lattice element (the smallest cubic subcomponent of the maze) to another. This was computed in each of the *X*, *Y* and *Z* axes because the lattice edges and bars were aligned with these. We considered a ‘crossing’ to have occurred when the head of the rat moved from one unit to another. A detailed analysis of the behavioral data can be found elsewhere^17^ and so only brief results related to grid cell activity are reported in the present study.

### Spike sorting

Single unit activity was analyzed offline using a combination of MATLAB functions and spike-sorting software. First, the dimensionality of the waveform information was reduced to the first three principal components and amplitude. Based on these parameters, an automated spike-sorting algorithm (Klustakwik v.3.0 (ref. ^33^)) was used to distinguish and isolate separate clusters. The clusters were then further checked and refined manually using a cluster cutting GUI (TINT v.4.4.12, Axona). As well as the previously mentioned features, manual cluster cutting also made use of spike auto- and cross-correlograms.

### Firing rate maps

To generate 3D volumetric firing rate maps we used an adaptive binning method described previously^34,35^. Briefly, for every bin a circle centered on the point was gradually expanded until the following criterion was met:

$$r > \alpha /n\sqrt s$$

where *α* is a constant, *r* is the radius of the circle in pixels, *n* is the number of occupancy samples falling within the circle and *s* is the total number of spikes falling within the circle. Once this criterion has been met, the firing rate assigned to the point was equal to *s*/*n*. For our maps, *α* was set to the value 1,600 and we calculated the firing rate across a 2.5-cm^3^ grid. Periods where the animals were moving <5 cm s^−1^ were not included in any firing rate maps to avoid potential contamination by a low-speed phenomenon (sharp-wave/ripple activity, licking behavior, and so on).

When calculating the stability of spatial activity between volumetric maps to avoid inflated comparisons based on many small voxels, we instead made maps using a standard histogram procedure (MATLAB histcn, B. Luong). For this, data were binned using a much larger 10-cm^3^ grid and smoothed using a Gaussian kernel with an s.d. of 2.5 bins (MATLAB imgaussfilt3). For 2D maps, such as the Cartesian planar projections, we also generated firing rate maps using a standard histogram procedure (MATLAB histcounts2). For this, data were binned using a 2.5-cm^3^ grid and smoothed using a Gaussian kernel with an s.d. of 1 bin (MATLAB imgaussfilt).

### Recording stability between arenas

To verify that cells were stably recorded during our maze sessions we used a similar approach to one described previously^36^. First we computed Pearson’s pairwise correlation (MATLAB corr) between the first and second arena rate maps recorded before and after each lattice maze session. For this we used the 2D *XY*-projected rate maps described above (Firing rate maps). For comparison we correlated first and second arena sessions from random grid cells while maintaining their temporal order (that is, first arena versus second arena from a random grid cell). This shuffle was repeated 5,000 times. The results of this analysis can be seen in Extended Data Fig. 2. See also Waveform stability for an approach independent of spatial activity.

### Waveform stability

As a test for cluster recording stability that is not reliant on spatial activity maps, we computed average waveforms for each tetrode channel (four channels total). We then calculated the Euclidean distance between these waveforms on each tetrode channel (50 samples, sampled at 48 kHz, MATLAB pdist2) between session pairs (Arena 1 versus Lattice, Lattice versus Arena 2 and Arena 1 versus Arena 2) and averaged across all four channels. In the resulting values, small distances indicate unchanging waveform shape and stable recording across all four channels.

On their own these values can highlight whether one recording session was consistently unstable relative to the others, but to assess the general stability of all sessions we generated chance distributions for comparison. For these we repeated the above procedure but compared pyramidal cells (neurons with a peak-to-trough width of waveform >250 µs) that were co-recorded on the same tetrodes. The same tetrodes were used for comparable electrode and recording system impedance. Only putative pyramidal cells were used, so that comparisons would be between cells with similar waveform characteristics (most of the recorded grid cells were also assumed to be pyramidal). For example, the waveforms for one pyramidal cell recorded in arena 1 could be compared with the waveforms of another pyramidal cell co-recorded on the same tetrode in the lattice. These chance distributions reflect the change in Euclidean distance that could be expected if cells randomly shifted position relative to the recording tetrode while remaining detectable. Spurious values >300 µV were excluded from the analysis.

### Spatial stability within sessions

To test the within-session stability of spatial representations, we divided maze sessions into two halves of equal length (first 50% and second 50%) and computed Pearson’s pairwise correlation (MATLAB corr) between the firing rate maps for these halves. The two dimensions were generated from data projected on to the Cartesian coordinate planes and we also compared volumetric maps generated as multivariate histograms with 10 cm^3^ voxels smoothed using a Gaussian with a 2.5-bin s.d. (MATLAB imgaussfilt3). Large bins and smoothing were used here to reduce the huge number of spatial bins compared between maps (from around the 9 × 10^4^ voxels found in adaptively binned rate maps to around 2 × 10^3^ voxels). Examples of these maps can be seen in Extended Data Fig. 3a. In both cases we compared the observed correlation values with shuffled distributions generated by comparing session halves from random grid cells (that is, first 50% versus second 50% from a random grid cell). This shuffle was repeated 5,000 times for each map type. The results of this analysis can be seen in Extended Data Fig. 3.

### Stability between movement epochs

To test the stability of spatial representations when rats were moving vertically versus horizontally, we divided maze sessions based on the animal’s movement direction (defined in Behavior and spherical heatmaps). This consisted of filtering trajectory and spike data to include only vertical movements (defined as movements at a pitch >30° or <−30°) or only horizontal movements (defined as movements at a pitch <30° and >−30°). We used a pitch angle of 30° to delineate movements because this divides the face of a sphere into two halves with equal surface area (a ‘belt’ around the equator for one half and two spherical caps for the other). An example filtered trajectory can be seen in Extended Data Fig. 4a,b. We then repeated the analysis described in Spatial stability within sessions. The results of this analysis can be seen in Extended Data Fig. 4.

### Spatial information and sparsity shuffles

To determine whether the firing of grid cells was less homogeneous (that is, more ‘clumped’) than chance, we generated firing rate maps using a standard histogram procedure (MATLAB histcn, B. Luong) with 2 cm^3^ voxels and smoothed using a Gaussian kernel with an s.d. of 2 voxels (MATLAB imgaussfilt3). We then calculated spatial information content (bits s^−1^) as:

$${\mathrm{Spatial}}\;{\it{{\mathrm{information}}}} = \mathop {\sum }\limits_{i = 1}^N p_i\frac{{\lambda _i}}{\lambda }\log _2\frac{{\lambda _i}}{\lambda }.$$

Next, for each grid cell we shuffled its spike train 100 times by random increments of 0.02 s (minimum 20 s), and for each shuffle we re-computed a firing rate map and spatial information as above. Last, we expressed the observed spatial information values in s.d.s from the shuffle:

$${\mathrm{Standardized}}\;{\mathrm{value}} = \frac{{\left( {{\mathrm{Observed}}\;{\mathrm{value}} - \mu _{{\mathrm{shuffle}}}} \right)}}{{\sigma _{{\mathrm{shuffle}}}}}.$$

This essentially *z*-scores the observed values relative to the shuffles. Scores >1.96 exceed the shuffle 95th percentile and thus deviate significantly from the shuffle at the 0.05 level. Similar results were obtained using sparsity (data not shown).

### Directional analyses and shuffles

As we did not have access to 3D head direction information, we estimated instantaneous projected azimuthal head direction as:

$$\theta \left( t \right) = {\mathrm{tan}}^{ - 1}[{\Delta}y^t/{\Delta}x^t]$$

where ${\Delta}x^t$ and ${\Delta}y^t$ represent the change in *X* or *Y* position, respectively^19^, for which we used the smoothed and interpolated position tracking (Trajectory reconstruction) ignoring movements in the *Z* axis. To generate head direction tuning curves, we binned position and spike directions into 6° bins, smoothed the resulting histograms with a Gaussian kernel (MATLAB imgaussfilt, σ = 3 bins, circular padding) and then generated a tuning curve by dividing the spike histogram by the time spent in each bin. From these we calculated, as a measure of directionality, the Rayleigh vector length (MATLAB circ_r, circular statistics toolbox^37^) and preferred firing direction as the bin containing the maximum firing rate.

Next, for each cell we shuffled its spike train 100 times by random increments of 0.02 s (minimum 20 s) and for each shuffle recomputed a directional rate map and statistics as above. A cell was categorized as directionally modulated if it exhibited a Rayleigh vector greater than the 95th percentile of the shuffled values in both arena sessions and fired at a rate >0.1 Hz in both.

Last, to determine whether directionally modulated cells maintained their allocentric firing directions across mazes (all recordings were made in the same location in the same room), we correlated the tuning curves for all cells as a population in the arena to their tuning curves in the lattice. For comparison we circularly shifted each cell’s lattice tuning curve independently by a random number of bins (between 1 and 60) and recomputed the correlation. We repeated this 1,000 times and, if the original correlation exceeded the 95th percentile of the shuffled values, we considered that the cell population firing was more stable than chance; this difference was also expressed as a standardized *z*-score as described above (Spatial information and sparsity shuffles).

### Autocorrelations

For 2D and 3D autocorrelations, *r*, of each grid cell’s firing rate map was calculated according to:

$$\begin{array}{ll}r\left( {\tau _x,\tau _y,\tau _z} \right) \\ = \frac{{M\mathop {\sum }\nolimits_{x,y,z} \lambda (x,y,z)\lambda (x - \tau _x,y - \tau _y,z - \tau _z) - \mathop {\sum }\nolimits_{x,y,z} \lambda (x,y,z)\mathop {\sum }\nolimits_{x,y,z} \lambda (x - \tau _x,y - \tau _y,z - \tau _z)}}{{\sqrt {\left[ {M\mathop {\sum }\nolimits_{x,y,z} \lambda \left( {x,y,z} \right)^2 - \left[ {\mathop {\sum }\nolimits_{x,y,z} \lambda (x,y,z)} \right]^2} \right]\left[ {M\mathop {\sum }\nolimits_{x,y,z} \lambda \left( {x - \tau _x,y - \tau _y,z - \tau _z} \right)^2 - \left[ {\lambda (x - \tau _x,y - \tau _y,z - \tau _z)} \right]^2} \right]} }}\end{array}$$

where $\lambda (x,y,z)$ is the firing rate at the location (*x*, *y*, *z*) in the firing rate map, *M* is the total number of voxels in the rate map and *τ*_*x*_, *τ*_*y*_ and *τ*_*z*_ correspond to *x*, *y* and *z* coordinate spatial lags^19^.

### Autocorrelation self-similarity

As a measure of firing rate map self-similarity along each axis we extracted, for each grid cell, the autocorrelation values falling along the autocorrelation midlines (MATLAB interp3 with linear interpolation). Intuitively, if a volumetric firing rate map contained circular columns that spanned the entire *z*-axis, then the autocorrelation would also contain a columnar peak in its center, spanning the entire *z*-axis. Thus, values falling along a line drawn straight down the middle of the autocorrelation from top to bottom would all be high, whereas values falling on a line drawn straight across the autocorrelation from one side to the other through the middle would peak near the center, but otherwise contain low values.

### Autocorrelation correction for anisotropy

As in place cells^7^ some grid fields were significantly elongated, if these fields formed a close-packed arrangement this could take two forms. First, fields could be arranged isotropically (field centroids forming equilateral tetrahedra) while remaining individually elongated, perhaps resulting in the overlap of vertical layers. Although this should not present a problem for the planar symmetry analysis proposed by Stella and Treves^12^, a second possibility is that the underlying arrangement itself could be elongated (field centroids forming acute and obtuse tetrahedra) with fields elongating to fill the volume between them. This latter configuration would disrupt the angular relationships between packing layers and the planar symmetry analysis.

To account for this, we repeated the planar analysis after correcting grid cell autocorrelograms to remove anisotropy introduced by field elongation. This would also correct anisotropic close-packed arrangements, allowing FCC and HCP arrangements to be identified. For this, we thresholded autocorrelations at a correlation value of 0.25 (MATLAB imbinarize) and extracted the central region. This central region should reflect the average characteristics of the firing fields in the firing rate map, their elongation and orientation. Using this fact, we resized the autocorrelation along each dimension by the amount necessary to ‘correct’ this central peak into a sphere (MATLAB imresize3 with cubic interpolation and anti-aliasing), thus, correcting any anisotropy in a potentially close-packed arrangement (see Supplementary Fig. 7a for an example).

### Grid score

Gridness scores were calculated similarly to previous studies^1,38^. The 2D autocorrelogram was thresholded to leave only values >0.3 and the seven most central correlation peaks were found. The peak closest to the center of the autocorrelation was excluded and the annulus concentric with the autocorrelogram that contained the other six peaks was isolated. The inner/outer radii defining this annulus were chosen as ±*r*, where *r* was the estimated radius of the most central peak. Pearson’s correlations between rotationally offset copies of the annulus were computed. Gridness score (also called hexagonal gridness score (HGS)) was calculated as the minimum correlation obtained at rotational offsets 60° and 120° minus the maximum obtained at 30°, 90° and 150°, which results in a high value when the autocorrelation exhibits a hexagonal structure and a low value otherwise. In addition, we calculated an equivalent score for a square firing pattern (also called square gridness score (SGS)) as the minimum correlation obtained at rotational offsets 90° and 180° minus the maximum obtained at 45°, 135° and 225°^19^.

### Grid score shuffles

To determine whether a cell’s HGS or other spatial parameter was greater than could be expected by chance, for each session we used a bootstrap-versus-shuffle approach^15,39^. First, estimated spatial parameter values (HGS, SGS, speed score) were obtained by resampling spikes using a bootstrap with replacement procedure (100 iterations). At each iteration, we recreated a firing rate map and spatial autocorrelation, and recalculated the spatial parameter values. Spatial parameter values were then estimated as the median of the collected bootstrapped values.

Next, we repeated the same procedure (100 iterations), but instead of resampling spikes we circularly shifted the spike train of the cell by a random 0.2 s increment (>20 s). If the median parameter value obtained from bootstrapping exceeded the 95th percentile of the values obtained from the shuffles, the spatial parameter was considered to be greater than expected by chance.

### Grid cell criteria

After completing the parameter shuffles described above, a cell was considered to be a grid cell if its grid score exceeded that expected by chance in both arena sessions (recorded before and after the lattice session). The results of this analysis can be seen in Extended Data Fig. 2b–d.

### Grid fields

Firing fields were detected in adaptive binned firing rate maps (Firing rate maps) as regions of >64 contiguous voxels with a firing rate >30% of the map’s peak value (MATLAB imbinarize and regionprops3). In addition, each field had to have a peak firing rate >1 Hz and rats had to visit it more than five times during a session. We then extracted field properties such as volume, centroid, principal axis lengths, eigenvectors and eigenvalues using previously established methods^7^ (MATLAB regionprops3).

### Grid field distribution in the lattice

To test whether grid cell firing fields were distributed uniformly throughout the lattice maze, we pooled fields from all grid cells and binned them according to their lattice layer along each axis of the maze. We then compared the proportion of fields in each layer with a chance distribution. Chance was computed by generating *n* random points within a volume matching the lattice 1,000 times and repeating the above process, where *n* is the number of real grid cells. Intuitively we would expect this chance distribution to be centered on 1/6 because there are six lattice layers along each axis. If the observed proportion of fields in a layer exceeded the lower or higher 99th percentiles of this chance distribution, it was considered to be significantly under- or overrepresented, respectively. For visual assessment, we also plotted the same data for all grid cells individually (Supplementary Fig. 3a).

### Grid fields per cubic meter

To estimate the practical volume of our 3D mazes we calculated an average dwell time map across all sessions and animals. We then thresholded this map so that only bins containing an average dwell time >0.1 s remained. The maze volume was then estimated as the total volume of these remaining voxels. In this way the arena was estimated as 0.4 m^3^ and the lattice as 1.2 m^3^.

### Grid field size

Grid field size was estimated as the radius of a sphere with a volume equivalent to that of the 3D autocorrelation central peak after thresholding at 0.25. This method was validated on simulated FCC arrangements with different field sizes (Supplementary Fig. 4a,b).

To test whether the volume of firing fields was consistent within grid cells for each grid cell we detected its firing fields (Grid fields) and calculated the CV (*µ*/*σ*) of their volumes. A low CV indicates low variability. Next, we pooled all the grid fields, shuffled their cell identities and repeated the above process. We repeated this 100 times and then compared grid cell CV values with the ones obtained from the shuffles.

### Grid field spacing

Grid spacing was estimated by expanding a sphere outward from the center of the 3D autocorrelation, in steps of 1 bin up to a radius 0.6× the maximum side length of the autocorrelation, and calculating the median correlation in bins within a distance of 2 bins from the surface of the sphere. Average field spacing was then estimated as the location of the first peak in the median correlation values after excluding the central autocorrelation peak (MATLAB findpeaks, with a minimum peak prominence of 0.01 and excluding peaks at a distance <5 bins). This method was validated on simulated field arrangements and correctly estimates grid spacing regardless of the underlying configuration (Supplementary Fig. 4c,d). The average median correlation values for all grid cells can be also be seen in Supplementary Fig. 4e.

### Grid field orientation

We extracted each grid field’s orientation and principal axes, which were defined as the orientation and major axes of an ellipsoid with the same normalized second central moments as the field region. In more detail, we calculated the second central moments or covariance matrix that best described a thresholded place field, in effect fitting a multivariate normal distribution to the field. The direction and magnitude of the best-fit ellipse that describes the place field are then given by the eigenvectors and eigenvalues of this covariance matrix respectively (MATLAB regionprops3).

To determine whether fields were oriented in three dimensions along one or more arbitrary axes, we projected the field eigenvectors and their antipodal equivalents on to a unit sphere. We then extracted the number of fields falling within regions on the surface of the sphere corresponding to the intersection of the sphere and the Cartesian *XYZ* axes. These regions are equivalent to ~60° conic sections centered on each respective axis in one direction from the origin, so for each axis we combined the two corresponding directional regions.

To determine whether more fields were parallel to an axis than would be expected by chance, we generated 1,000 random points on the face of a sphere and counted the proportion of points falling within the area around each axis. We did this 1,000 times. Chance was calculated as the interval between the 2.5th and 97.5th percentile ranks of this distribution. If the observed field count for an axis exceeded the upper threshold, it was considered to be overrepresented with respect to chance.

For visualization, we calculated the von Mises–Fisher kernel smoothed density estimate of these grid field vectors across the sphere’s surface. Briefly, the Gaussian used was defined as:

$$g\left( x \right) = e^{\left( { - 0.5\left( {\frac{x}{\sigma }} \right)^2} \right)}$$

where *x* was defined as the inverse cosine of the inner dot product between each vector point and points across a sphere’s surface (MATLAB sphere) and *σ* was the s.d. of the Gaussian, which was set to 10. In this way, the resulting 3D heat plots give a density estimate of points on the sphere, where density is estimated as the sum of the Gaussian weighted distances (along the surface of the sphere) to every data point. These 3D spherical maps are presented in the main text for visualization only. See Supplementary Fig. 9 for a schematic explanation.

### Grid field elongation

After extracting a field’s principal axis lengths (Grid field orientation) we calculated the elongation index as:

$${\mathrm{Elongation}} = \frac{{P1}}{{0.5(P2 + P3)}}$$

where *P*1, *P*2 and *P*3 are the principal axes from the largest to the smallest, respectively. This gives a measure of the curvature of the place field: large elongation values represent elongated fields whereas a value of 1 would represent a sphere. In the case of the arena, where animals were mainly restricted to horizontal (2D) movements, elongation was calculated using the first two principal axes (*P*1/*P*2).

We then tested whether this elongation index deviated significantly from a distribution that would be expected by chance using an analysis inspired by one reported previously^5^. For each field we defined a perfect sphere, centered on the field’s centroid. The diameter of this sphere was calculated such that it would share the same convex volume (the volume of the convex hull enclosing the field voxels) as the place field. This was calculated as:

$${\mathrm{Equivalent}}\;{\mathrm{diameter}} = 6\left( {\frac{{vf}}{\pi }} \right)^{\frac{1}{3}}$$

where *vf* is the convex volume of the place field. This is more accurate than the geometric mean approach reported previously^5^, which assumes that all place fields are perfectly elliptical and thus tends to underestimate equivalent diameter. Next, the spikes emitted within the place field were randomly shuffled among the trajectories through this sphere using a multivariate Gaussian process (MATLAB normrnd). The mean of the Gaussian was the sphere center, and the s.d. of the Gaussian was set at 1.8× the radius of the sphere (to approximate the 20% thresholding used during field detection). Each spike was then assigned to the position of the nearest trajectory data point (MATLAB knnsearch). The result of this procedure was a normally distributed point cloud of spikes centered on the centroid of the original field, with the same equivalent diameter and firing rate. This is more realistic than the previous approach which spread spikes uniformly^5^.

We then recomputed the firing rate map for these shuffled spikes and extracted its elongation index as described above. This procedure was repeated 100 times for each place field. We then expressed the observed field elongation as a *z*-score of these 100 shuffles; place fields with an elongation index that could be expected, by chance, from an underlying spherical field (that is, with an elongation index lower than the 95th percentile rank of the shuffled distribution or lower than *z* = 1.96) were defined as spherical or isotropic: otherwise, place fields were defined as nonspherical, anisotropic or elongated.

### Grid field local order

To estimate the local ordering of grid firing fields we used a method inspired by one described previously^23^. We first generated firing maps as standard histograms with 2 cm^3^ voxels smoothed using a Gaussian kernel with an s.d. of 2.5 bins (MATLAB imgaussfilt3). We thresholded these maps to leave only those values >10% of the peak firing rate. We then found field centroids as regional maxima of the H-maxima transform (MATLAB imextendedmax, H = 0.8). Centroids with a distance <25 cm between them were iteratively replaced with the average of the two centroids.

For each field we then found the distance to its three nearest neighbors. After discarding repeated distances, we calculated the CV (s.d./mean) of all interfield distances. A low CV value indicates that distances were consistent.

Next, we circularly shifted the spike train of the cell by a random 0.2-s increment (>20 s) and generated a firing rate map as above using this shuffled spike train. If the number of fields detected in this map was similar to that observed in the original map (±3 fields), we repeated the CV analysis described above, otherwise the shuffle was discarded. We aimed to collect a maximum of 1,000 of these shuffles per cell, but the procedure was halted if this number was not achieved after 10,000 shuffles. Thus, some grid cells may have less than the maximum 1,000 shuffles (mean and s.d. total shuffles: 767 and 386). To determine whether the local ordering of fields was higher in grid cells than the shuffles, we compared the observed grid cell CV values with the average of their corresponding shuffles (that is, paired comparison of grid cell CV values and average shuffle values). Note that no grid cells, in the arena or lattice, exceeded the 95th percentiles of the shuffled distributions, suggesting that this approach is not sensitive at detecting consistent interfield distances.

### Planar symmetry analysis

FCC is a cubic lattice structure that results from stacking hexagonally arranged layers of spheres in the sequence ABC, where layers B and C are offset hexagonal patterns that rest in the spaces between the spheres below. HCP is a similar lattice structure that describes an ABA sequence (Fig. 1a and Supplementary Fig. 5a)^11,12,19,20^.

Where the autocorrelogram of a 2D grid cell firing rate map resembles a hexagonal grid, centered on a central peak, the autocorrelogram of a 3D FCC configuration also reproduces the same 3D pattern spanning around a central peak^12^. If the field configuration is aligned to the XY axes, a horizontal plane cut through the center of the autocorrelation will pass through a hexagonal field arrangement, and the correlation values falling on this plane will present a high grid score. In addition, three further planes angled at a pitch of 72° from the *XY* plane can be found that also pass through hexagonal arrangements and present high grid scores. These planes will also be arranged with 120° between them in azimuth. In addition, due to the cubic nature of the FCC arrangement (another name for FCC is cubic-close-packed), there are also three planes at a pitch of 57° from the *XY* plane, arranged with 120° between them in azimuth, offset from the 72° hexagonal planes by 60° in azimuth, that transect fields arranged in a square formation. In this way, extracting every possible plane through the autocorrelation center, and mapping their HGS and SGS, respectively, can, in turn, be used to determine the likelihood of an FCC arrangement (Supplementary Figs. 5b,c and 6).

By contrast the autocorrelation of an HCP configuration, while still centered around a peak, does not exactly resemble the original pattern. Although there is still a ‘best plane’ corresponding to a horizontal shift of the grid pattern (as in the 2D case) due to the half overlap of peaks between layers, there are a further six layers that pass through peaks resembling a hexagonal grid^12^. Assuming a field arrangement aligned to the *XY* axes, a horizontal plane cut through the center of the autocorrelation will pass through a hexagonal field arrangement and the correlation values falling on this plane will present a high HGS. In addition, six further planes angled at a pitch of 72° from the *XY* plane can be found that also pass through hexagonal arrangements. However, three of these planes present high HGS (arranged with 120° between them in azimuth), whereas the other three present low HGS (arranged 60° offset to the other planes in azimuth). In an HCP arrangement, planes with square field arrangements can also be found, again at 57° from the *XY* plane in pitch and at the same azimuthal angles as the hexagonal ones (Supplementary Fig. 6).

Last, the autocorrelation of a hexagonal columnar field arrangement is the same arrangement centered around a columnar peak. Assuming that the columns are parallel to the *z*-axis, we would expect horizontal slices to present high grid scores that would decrease as the pitch of the slices diverges from the horizontal (Supplementary Figs. 5c and 6).

In these examples, we assumed that the configurations are aligned to the *XY* axes, but, once grid scores have been mapped for every azimuth and pitch slice combination, it is possible to estimate the 3D orientation of the arrangement using the ‘best plane’ or the transecting plane with the highest grid score. In the FCC case, all four planes are equally ‘best’, but this is useful for HCP and columnar arrangements. In all cases it allows us to correct grid cell autocorrelations, rotating them so that the best plane is always horizontal (Supplementary Fig. 7a).

### Structure scores (χ_CP_, χ_FCC_, χ_HCP_ and χ_COL_)

Once we collected the HGS and SGS for every possible plane transecting a grid cell autocorrelation, we looked to calculate scores that could be used to differentiate the different field configurations. We used an approach similar to that proposed by Stella and Treves^12^, but also took into account the square grid scores, which allowed us to differentiate FCC and HCP arrangements based solely on their autocorrelations. We also extended this analysis to include a score for columnar arrangements (Supplementary Fig. 6).

We built volumetric firing rate maps (Firing rate maps) and autocorrelations (Autocorrelations), and then extracted planes transecting the autocorrelation (65 pitch angles and 65 azimuth angles for a total of 4,225 planes; MATLAB sphere and obliqueslice). For each plane we calculated its HGS and SGS (Grid score). We next found the ‘best plane’ as the one associated with the maximum HGS, and corrected the autocorrelogram so that this plane would form the *XY* horizontal (Supplementary Fig. 7a). We then interpolated the HGS and SGS maps up to 128 pitch and azimuth angles using a spherical nearest neighbor method, where nearest neighbors were found based on the inverse cosine of the inner dot product between each vector point and points across a sphere’s surface (MATLAB sphere).

Next, we extracted the HGSs found at a 72° pitch from the best plane and the SGSs found at a 50° pitch from the best plane. As both FCC and HCP are expected to exhibit high HGSs and SGSs at these respective angles, we calculated a general quality score (χ_CP_) as the median of these two distributions combined.

For an FCC score (χ_FCC_) we found the three 120° offset azimuth angles at 72° pitch from the best plane with the maximum total hexagonal grid score. We then calculated *β* as the median square grid score found at the same azimuthal angles and at a 50° pitch and *α* as the median square grid score found at 60° offsets from these in azimuth and at a 50° pitch (Supplementary Fig. 6 left). From these:

$${\upchi}_{{\mathrm{FCC}}} = \alpha - \beta.$$

For the HCP score (χ_HCP_) we found the three 120° offset azimuth angles at 72° pitch from the best plane, with the maximum total hexagonal grid score. We then calculated *α* as the median square grid score found at the same azimuthal angles and at a 50° pitch, and *β* as the median square grid score found at the same azimuthal angles and at a 72° pitch (Supplementary Fig. 6, middle). From these:

$${\upchi}_{{\mathrm{HCP}}} = \alpha - \beta.$$

For the columnar score (χ_COL_), we calculated *α* as the median hexagonal grid score found at all pitch angles within 60° of the best plane and *β* as the median of all remaining grid scores (Supplementary Fig. 6, right). From these:

$${\upchi}_{{\mathrm{COL}}} = \alpha - \beta.$$

### Simulated field arrangements

Where *S* was the side length of a hexagon, points were separated by *S* along the *x* axis and $\sqrt 3 (0.5S)$ along the *y* axis, with every second row of points along the *y* axis offset by *S*/2 in *x*; this gives points tiling horizontal space in a hexagonal arrangement. For our simulations, *S* was a value randomly drawn for each cell from the uniform distribution between 200 and 600 mm. For close-packed arrangements these horizontal points were then stacked along the *z* axis separated by $(\sqrt 6 /3)S$. For HCP, every second layer along the *z* axis (second onwards) was offset in *y* by $\sqrt 3 (1/3)S$. For FCC, every third layer (second onwards) along the *z* axis was offset in *y* by $\sqrt 3 (1/3)S$ and every third layer (third onwards) along the *z* axis was offset in *y* by $- 2(\sqrt 3 \left( {1/6} \right)S)$. For a columnar configuration, the horizontal hexagonal points were stacked continuously in *z*. For a random arrangement of fields, we generated *n* random points (MATLAB rand), where *n* was the number of points generated for an HCP arrangement, spread within the cuboid volume occupied by an HCP arrangement.

When the desired arrangement of points was generated, for every pixel of the simulated rate map we calculated the Euclidean distance to the nearest field point (MATLAB bwdist) and weighted these using the Gaussian:

$$g\left( x \right) = e^{\left( { - 0.5\left( {\frac{x}{\sigma }} \right)^2} \right)}$$

where *x* is the Euclidean distance and *σ* is the Gaussian s.d., which was set at 2. To ensure that the planar symmetry analysis was capable of differentiating field arrangements even when they are not aligned to the maze/gravity, the resulting simulated firing rate map was then rotated 30° around a random 3D axis (MATLAB imrotate3 and rand). Simulated field arrangements without this last rotation step can be seen in Supplementary Fig. 7a.

### Grid field shuffle

To generate fields in random positions while remaining as close to the real data as possible, we employed a field-shuffling technique described previously^40^. Briefly, adaptive binned firing rate maps were oversmoothed (Firing rate maps, MATLAB imgaussfilt3, sigma 3 bins) and firing fields were segmented through a watershedding procedure. For this, field peaks were detected as local maxima (MATLAB imextendedmax with an H-maxima of 0.2) and watersheds were calculated on the distance transform (MATLAB bwdist) of these peaks (MATLAB watershed).

The following analyses were then performed on the unsmoothed adaptive binned firing rate map: *n* uniformly random points were identified in an empty copy of the firing rate map where *n* was the total number of fields identified in the watershed procedure. For each segmented field (numbered 1 to *n*), the bin with the peak firing rate was copied to one of these random positions. Next, firing rate values were iteratively moved from the original rate map (O) to the new empty copy (E) after this procedure: for each field in turn (1 to *n*) the bin closest to the peak was moved from O to E while maintaining its position relative to the peak as closely as possible (the ‘ideal’ position). Values were not moved to the locations of unvisited bins in O nor could they overwrite values already moved to E, so where the ideal position was not available values were instead moved to the position nearest to it in city-block distance. This procedure was repeated for every field in turn and in the field order 1 to *n* iteratively, until every bin had been moved from O to a position in E.

Intuitively, this method randomly shuffles the positions of fields within a firing rate map but also preserves, as much as possible, the internal structure of each field. Furthermore, because every firing rate value is moved and both maps share the same number of elements, both maps have the same distribution of firing rate values, the same peak firing rate and the same number of unvisited bins. Unvisited bins also retain their spatial positions, which maintains the shape and configuration of any uneven sampling in the original firing rate map. Due to the computational time cost associated with this shuffling method when using 3D maps and the already large time cost of the planar symmetry analysis, this shuffle was performed only twice per cell. Example field-shuffled firing rate maps can be seen in Supplementary Fig. 8c.

### LFP analyses

Before analysis, all LFP data had their direct current offsets removed, slowly changing components and running line noise using the Chronux toolbox^41^ locdetrend function, which subtracts the linear regression line fit within a 1-s moving window. They were then resampled at 250 Hz using a polyphase anti-aliasing filter (MATLAB function resample, pchip interpolation).

To obtain a theta phase angle for each spike, LFPs were first bandpass filtered in the 6- to 12-Hz range (fourth-order Butterworth, MATLAB butter and filtfilt) before a Hilbert transform was applied to obtain the instantaneous phase angle (MATLAB hilbert). Instantaneous frequency was calculated as the derivative of this analytic signal (MATLAB instfreq) and instantaneous amplitude was calculated as its magnitude.

To assess the relationship between running speed and the theta oscillation, we compared the instantaneous theta power/amplitude at every position data point (every 20 ms) with the animals’ instantaneous running speed. Instantaneous speed was estimated as the total distance traveled in every 40-ms window. To quantify the relationship between speed and power. we fitted a linear regression model using a least squares approach (MATLAB polyfit, 1°) and extracted the slope, *y*-intercept and sum of squared error. We also performed the same procedures to test the relationship between running speed and instantaneous frequency.

To calculate general theta characteristics, we computed average power spectral densities for each recording session by first zero-padding LFP data to the next highest power of 2. A Welch spectral estimator was then applied to obtain the power spectral density (MATLAB pwelch, Hamming window, 8 segments, 50% overlap). This was computed for 500 logarithmically spaced points between 0 and 250 Hz. Theta power was estimated as the maximum power found in the theta band (6–12 Hz) and theta frequency was defined as the frequency associated with this maximum power.

### Running speed analyses

Instantaneous running speed was estimated as the total distance traveled in every 40-ms window. For each cell, instantaneous firing rate was estimated as the smoothed spike histogram (20-ms bins, 13-bin or 260-ms Gaussian smoothing window using MATLAB function fspecial). To quantify the relationship between speed and firing rate, we used an analysis similar to that described previously^42^. We binned the animals’ running speeds in 2 cm s^−1^ increments, and calculated the mean firing rate for each running speed bin and the total time spent moving at that speed. We then fitted a linear regression model to the average firing rate/speed data using a least squares approach (MATLAB function polyfit, 1°) and extracted the slope, *y*-intercept and sum of squared error.

### Spike phase and autocorrelation analyses

To quantify the intrinsic theta modulation of every place cell we used an analysis described previously^43,44^. For each cell we calculated the ±500 ms spike autocorrelation in 10-ms bins, normalized this to the maximum value found between 100 and 150 ms and removed values >1. Then we fit the following function to the remaining data:

$$y\left( t \right) = \left( {a \times \left( {\sin \left( {2\pi \omega t + \frac{\pi }{2}} \right) + 1} \right) + b} \right) \times \exp \left( { - \frac{{\left| t \right|}}{{\tau _1}}} \right) + c \times \exp \left( { - \frac{{r^2}}{{\tau _2^2}}} \right)$$

where *a, b*, *c*, *ω*, *τ*_1_ and *τ*_2_ were fit to the data using a nonlinear least squares method (MATLAB fit) and *t* is the autocorrelogram time lag. In simple terms this function fits a sine wave of frequency *ω* to the data, and the exponential term allows for this to decrease exponentially as the time lag increases (reflecting the exponential decay inherent in all spike autocorrelations). The last Gaussian term helps to center the fit on the autocorrelogram peak, which we found to be unnecessary in most cases. A measure of theta modulation strength was defined as *a*/*b*, which intuitively corresponds to the ratio of the sine fit relative to the baseline in the autocorrelogram. The parameter *ω* was extracted as the intrinsic theta modulation of the cell. We restricted possible values for *ω* to [6,12], *a* and *b* were restricted to non-negative values [0 Inf], *c* was restricted to [0,0.8], *τ*_1_ was unrestricted and *τ*_2_ was restricted to [0,0.05]. This fitting procedure was carried out only on cells that fired at least 500 spikes.

For each cell, the instantaneous theta phase of every spike was calculated by linear interpolation of the instantaneous theta phase signal described previously. These phase angles were binned between *−π* and *π* in 0.1-rad bins. The cell’s preferred theta phase was defined as the circular mean of these angles and the strength of this modulation was defined as the mean resultant vector length of these angles (MATLAB circ_mean and circ_r respectively, circular statistics toolbox^37^).

### Histology

At the end of the experiment animals were anesthetized, given an overdose of pentobarbital intraperitoneally (Euthatal, Merial Animal Health Ltd) and perfused with 0.9% saline solution followed by a 4% formalin solution. The brain was extracted and stored in 4% formalin for at least 7 d before any histological analyses. The brains were sliced sagittally in 30-µm sections on a freezing microtome at −20°. These sections were stained with a 0.1% Cresyl violet solution and the slice best representing the electrode track was then imaged. Histology results for every animal can be seen in Supplementary Fig. 2.

### Statistics and figures

Unless otherwise stated, we used two-tailed parametric tests (for example, MATLAB anova1 and ttest2) and post hoc tests compared population means (MATLAB multcompare, Dunn–Sidak correction). In all figures, * indicates significant at the 0.05 level, ** at the 0.01 level and *** at the 0.001 level. For all dot plots, black lines denote the s.d., empty circular markers denote the sample mean and filled markers represent individual data points. Data distributions were assumed to be normal but this was not formally tested. Data collection and analysis were not performed blind to the conditions of the experiments. No animals or data points were excluded from the experiment but putative cell clusters were manually curated to remove noise artifacts.

For the permutation tests described in Extended Data Fig. 7a we computed the mean difference between groups; we then pooled and shuffled samples, divided them into groups matching the original group sizes and recomputed the mean difference. We repeated this 1,000 times and estimated the probability of the outcome by *z*-scoring the original mean difference to the shuffled values and calculating a *P* value as the position of the absolute (one-sided test) *z*-value in the cumulative distribution function of a normal distribution with mean 0 and s.d. 1.

### Reporting Summary

Further information on research design is available in the Nature Research Reporting Summary linked to this article.

## Online content

Any methods, additional references, Nature Research reporting summaries, source data, extended data, supplementary information, acknowledgements, peer review information; details of author contributions and competing interests; and statements of data and code availability are available at 10.1038/s41593-021-00907-4.

## Supplementary information

Supplementary Information Supplementary Figs. 1–12 and Table S1.

Reporting Summary

Supplementary Video 1 Representative grid cell activity in the lattice maze from different animals and sessions—3D rotating spike and position plots with volumetric rate maps, projected rate maps and autocorrelations. Two-dimensional arena firing rate maps are also shown.

## Data Availability

A summary dataset is available for download^45^. The full raw dataset is available from the authors on request. Source data are provided with this paper.

## Source data

Source Data Fig. 1 Unprocessed histology images.
Source Data Fig. 2 Statistical source data.
Source Data Fig. 3 Statistical source data.
Source Data Fig. 4 Statistical source data.

## Extended data

Extended data are available for this paper at 10.1038/s41593-021-00907-4.

## Code availability

MATLAB code is available for download which, together with the summary dataset, can be used to regenerate all of the figures and analyses reported in the present study^45^. Code to analyze raw data is available from the authors on request.
