# Supplementary material for: Irregular distribution of grid cell firing fields in rats exploring a 3D volumetric space
Source: Nat Neurosci. 2021 Aug 11;24(11):1567–73. doi: 10.1038/s41593-021-00907-4 (PMC8553607; doi:10.1038/s41593-021-00907-4)
Supplement: Supplementary file 1 — Supplementary Figs. 1–12 and Table S1. [file 41593_2021_907_MOESM1_ESM.pdf]

nature portfolio

https://doi.org/10.1038/s41593-021-00907-4

Supplementary information

Irregular distribution of grid cell firing fields in rats exploring a 3D volumetric space

In the format provided by the
authors and unedited

Supplementary Materials for

Irregular distribution of grid cell firing fields in rats exploring a 3D volumetric space

Roddy M. Grieves, Selim Jedidi-Ayoub, Karyna Mishchanchuk, Anyi Liu, Sophie Renaudineau, Éléonore Duvelle and Kate J. Jeffery

This PDF file includes:

Supplementary Figures S1-12
Table S1

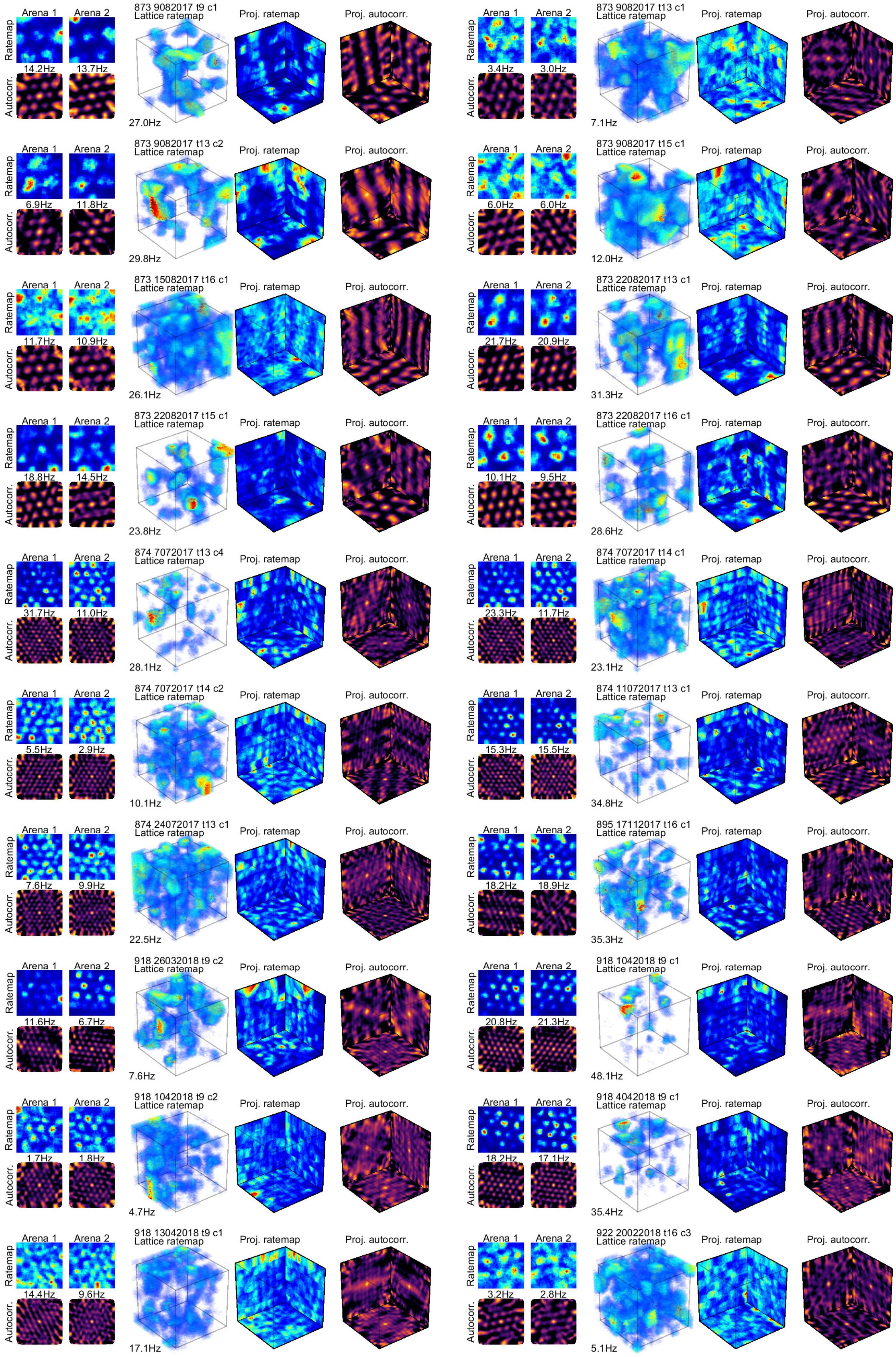
Arena 1
Arena 2
Ratemap
Autocorr.
Lattice ratemap
Proj. ratemap
Proj. autocorr.
873 9082017 t9 c1
14.2Hz
13.7Hz
27.0Hz
873 9082017 t13 c1
3.4Hz
3.0Hz
7.1Hz
873 9082017 t13 c2
6.9Hz
11.8Hz
29.8Hz
873 9082017 t15 c1
6.0Hz
6.0Hz
12.0Hz
873 15082017 t16 c1
11.7Hz
10.9Hz
26.1Hz
873 22082017 t13 c1
21.7Hz
20.9Hz
31.3Hz
873 22082017 t15 c1
18.8Hz
14.5Hz
23.8Hz
873 22082017 t16 c1
10.1Hz
9.5Hz
28.6Hz
874 7072017 t13 c4
31.7Hz
11.0Hz
28.1Hz
874 7072017 t14 c1
23.3Hz
11.7Hz
23.1Hz
874 7072017 t14 c2
5.5Hz
2.9Hz
10.1Hz
874 11072017 t13 c1
15.3Hz
15.5Hz
34.8Hz
874 24072017 t13 c1
7.6Hz
9.9Hz
22.5Hz
895 17112017 t16 c1
18.2Hz
18.9Hz
35.3Hz
918 26032018 t9 c2
11.6Hz
6.7Hz
7.6Hz
918 1042018 t9 c1
20.8Hz
21.3Hz
48.1Hz
918 1042018 t9 c2
1.7Hz
1.8Hz
4.7Hz
918 4042018 t9 c1
18.2Hz
17.1Hz
35.4Hz
918 13042018 t9 c1
14.4Hz
9.6Hz
17.1Hz
922 20022018 t16 c3
3.2Hz
2.8Hz
5.1Hz

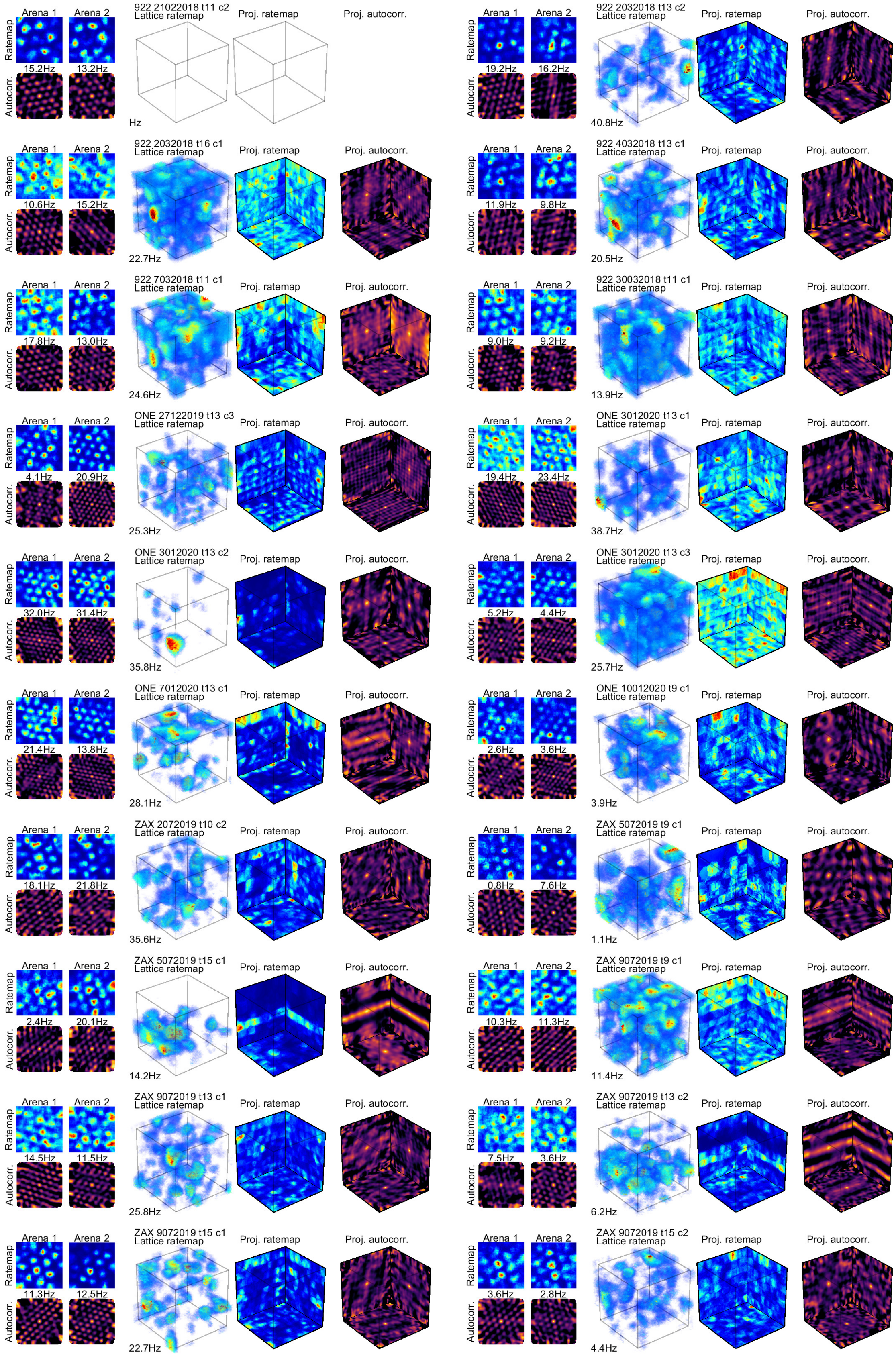
Arena 1
Arena 2
Ratemap
Autocorr.
Lattice ratemap
Proj. ratemap
Proj. autocorr.
922 21022018 t11 c2
15.2Hz
13.2Hz
Hz
922 2032018 t13 c2
19.2Hz
16.2Hz
40.8Hz
922 2032018 t16 c1
10.6Hz
15.2Hz
22.7Hz
922 4032018 t13 c1
11.9Hz
9.8Hz
20.5Hz
922 7032018 t11 c1
17.8Hz
13.0Hz
24.6Hz
922 30032018 t11 c1
9.0Hz
9.2Hz
13.9Hz
ONE 27122019 t13 c3
4.1Hz
20.9Hz
25.3Hz
ONE 3012020 t13 c1
19.4Hz
23.4Hz
38.7Hz
ONE 3012020 t13 c2
32.0Hz
31.4Hz
35.8Hz
ONE 3012020 t13 c3
5.2Hz
4.4Hz
25.7Hz
ONE 7012020 t13 c1
21.4Hz
13.8Hz
28.1Hz
ONE 10012020 t9 c1
2.6Hz
3.6Hz
3.9Hz
ZAX 2072019 t10 c2
18.1Hz
21.8Hz
35.6Hz
ZAX 5072019 t9 c1
0.8Hz
7.6Hz
1.1Hz
ZAX 5072019 t15 c1
2.4Hz
20.1Hz
14.2Hz
ZAX 9072019 t9 c1
10.3Hz
11.3Hz
11.4Hz
ZAX 9072019 t13 c1
14.5Hz
11.5Hz
25.8Hz
ZAX 9072019 t13 c2
7.5Hz
3.6Hz
6.2Hz
ZAX 9072019 t15 c1
11.3Hz
12.5Hz
22.7Hz
ZAX 9072019 t15 c2
3.6Hz
2.8Hz
4.4Hz

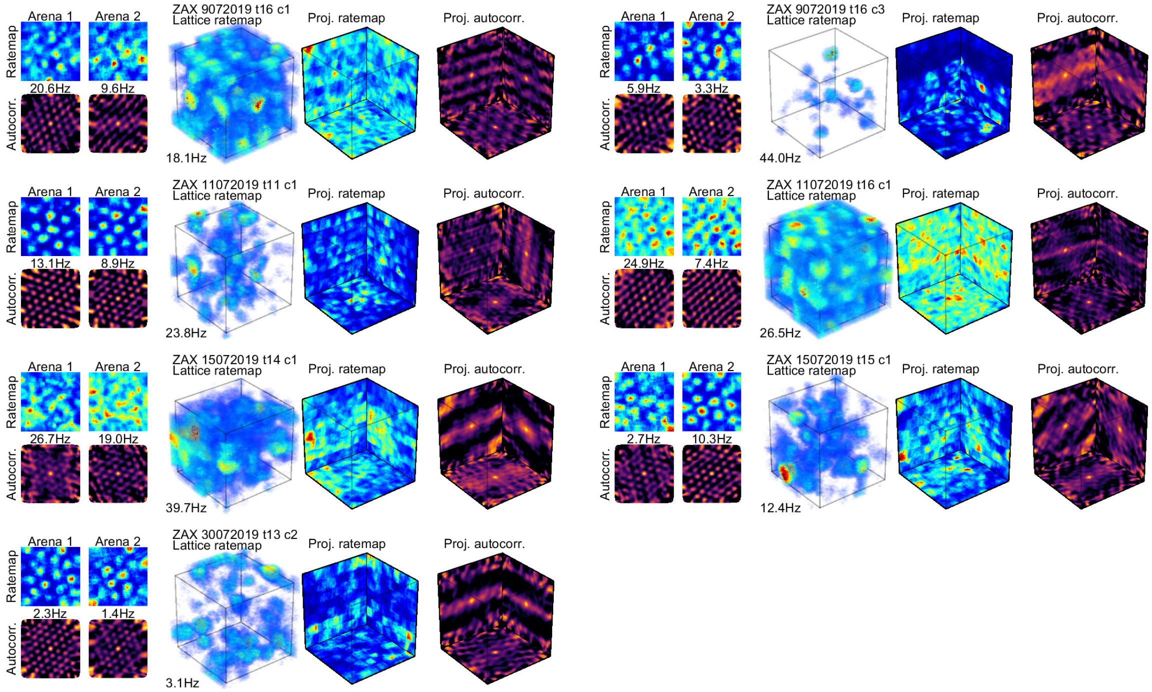

Fig. S1. Arena and lattice firing rate maps for all analysed grid cells.

Colorbars can be seen in Fig. 1. Two grid cells are shown per row. For each grid cell: Left) XY projected firing rate maps for the 1st and 2nd arena sessions respectively (top row) and autocorrelations of these (bottom row). Firing rate maps are scaled between 0Hz and the maximum firing rate value is given beneath the map. Autocorrelations are scaled between -0.2 and 1. Middle left) the volumetric firing rate map in oblique view, voxels with a firing rate less than 10% of the maximum are transparent. Lines show the outermost edges of the lattice maze. Text above the map gives the rat number, date, tetrode and cluster of the cell. Text below the map gives the maximum firing rate displayed. Middle right) projected planar firing rate maps shown in their planes of projection. These are scaled between 0Hz and the maximum value in all 3 maps. Lines show the outermost edges of the lattice maze. Right) autocorrelations of the projected firing rate maps shown in their planes of projection. These are scaled between -0.2 and 1. Lines show the outermost edges of the lattice maze. It is unknown why cell 922 21022018 t11 c2 did not fire in the lattice maze but was recorded in both arena sessions.

a Microdrive and wireless headstage

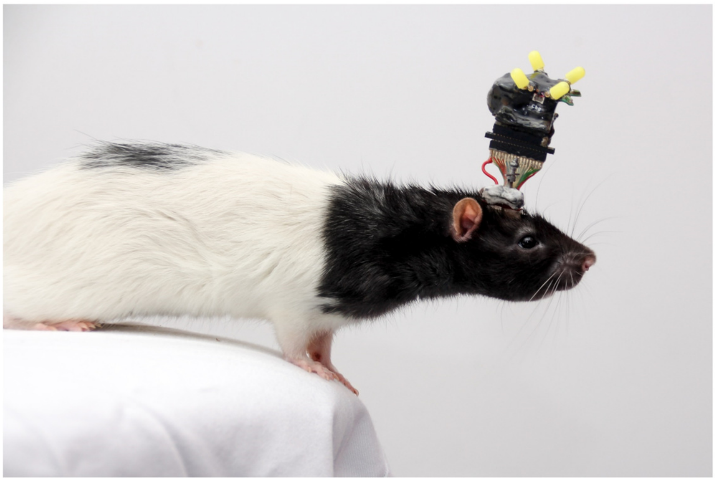

b Electrode tract histology for all animals

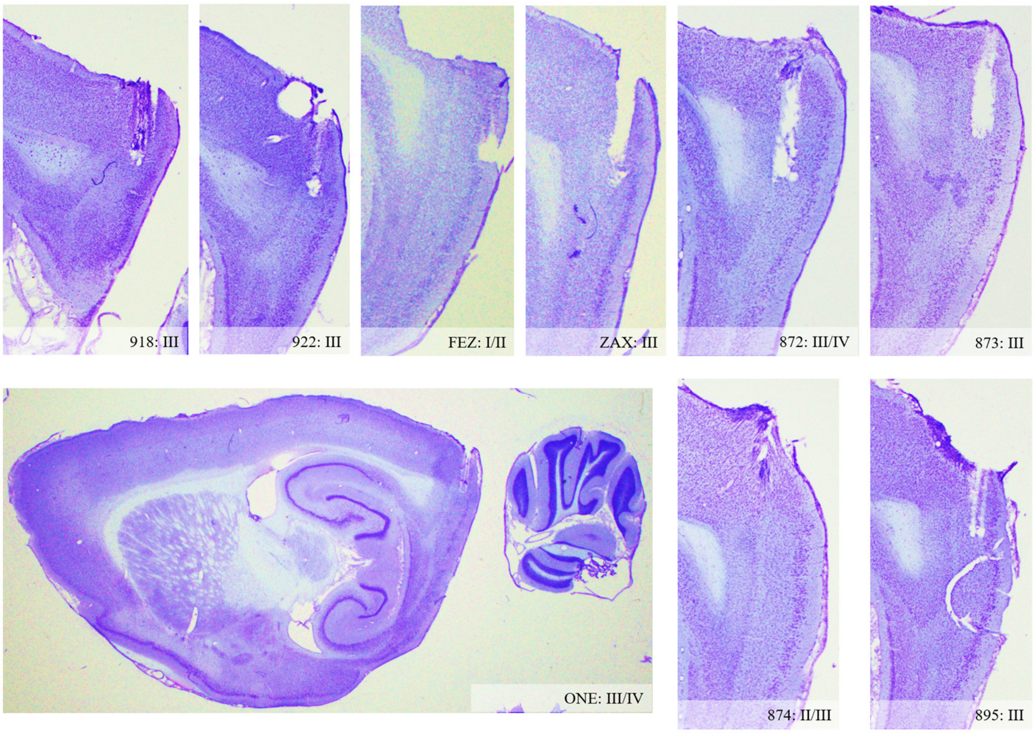

Fig. S2. Electrodes mainly targeted layer III of the mEC.

a, Photograph of a rat implanted with a microdrive and connected to the wireless headstage. **b**, Supplement to Fig. 1F. Histological Nissl-stained sections. For each rat, the sagittal brain section best demonstrating the position of the electrodes is given, text gives the animal identification and estimated layer of the mEC where recordings were made. Close-ups of the mEC are provided for every rat except rat 'ONE' where the whole brain section is given.

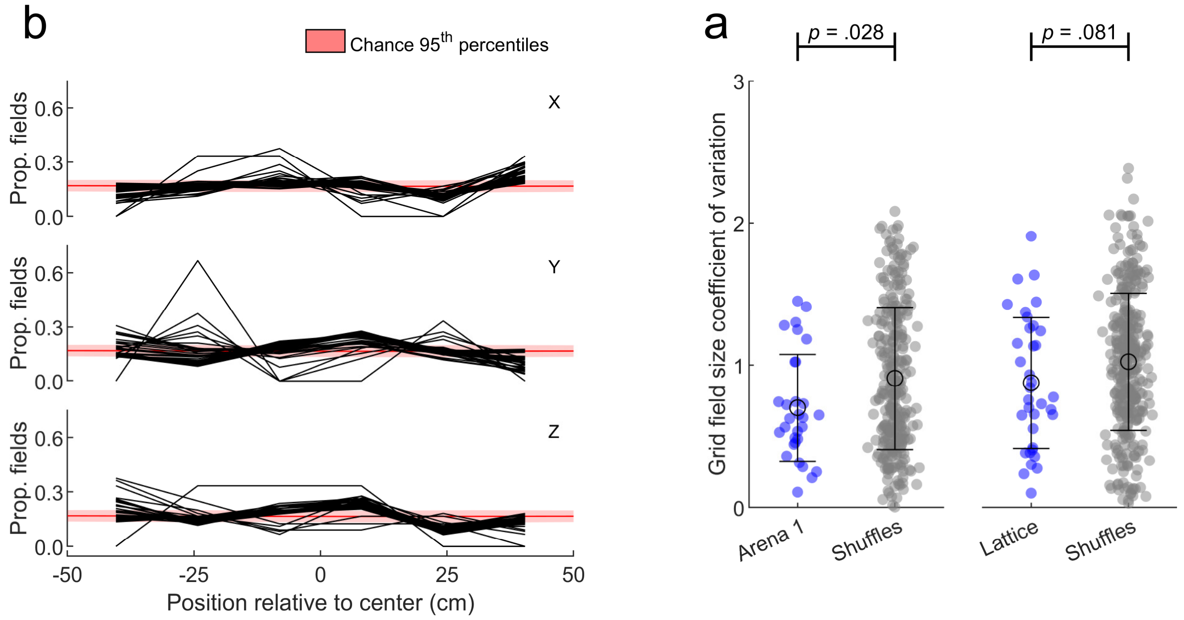

Fig. S3. Additional grid field characteristics in the lattice

Supplement to Figure 2D. **a**, Grid fields were distributed throughout the lattice. Proportion of fields in every lattice layer for all grid cells (n=47, black lines) in the X, Y and Z axes (top, middle and bottom). See Methods: *Grid field distribution in the lattice*. **b**, Grid fields were more variable in the lattice. Filled markers represent grid cells, open circles denote mean, error bars denote SEM. The consistency (coefficient of variation) of field volume within grid cells (n=47) and in shuffled data (n=470, see Methods: *Grid field size*). Lower values indicate that a cell's firing fields have consistent volumes. In the arena grid field volume was more consistent than chance ($F(1,328) = 4.9$, $p = .0280$, $\eta^2 = 0.0146$) as expected in grid cells. But this was not the case in the lattice, although this test is associated with a marginal probability ($F(1,383) = 3.1$, $p = .0815$, $\eta^2 = 0.0079$, one-way ANOVA).

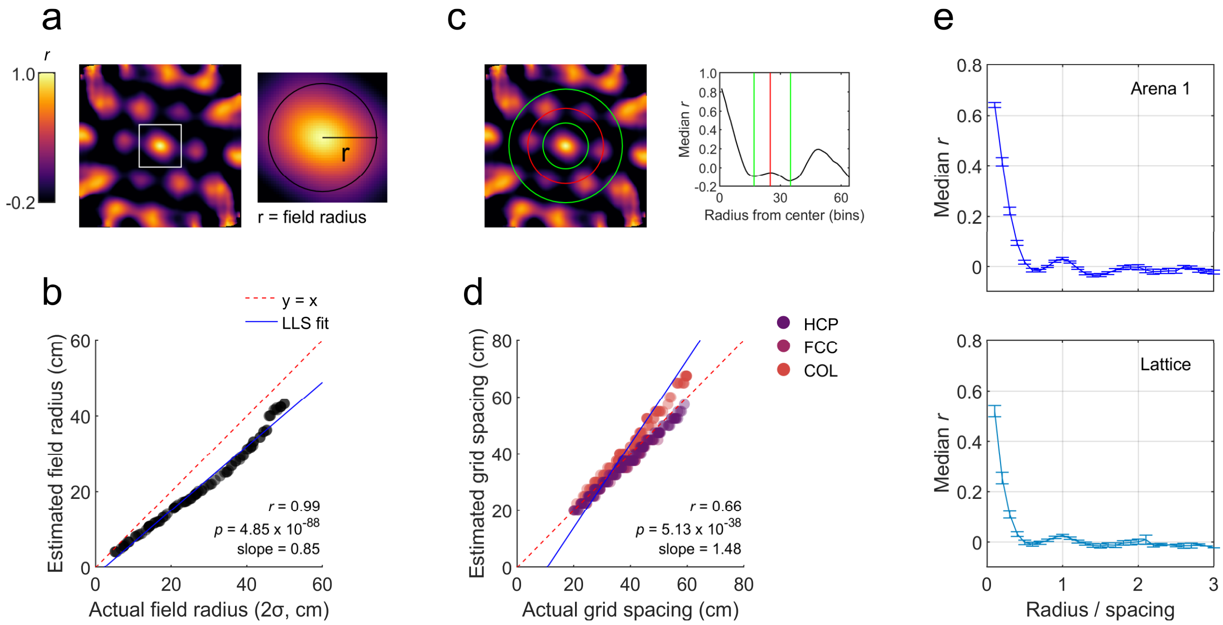

Fig. S4. Schematics and verification of the field radius and grid spacing analyses

a, Two-dimensional schematic showing the field size (radius) estimation procedure. The autocorrelation (top left) was thresholded at 0.25 and field size was estimated as the radius of the remaining central peak. **b**, This analysis was validated on simulated FCC field arrangements with varying field sizes (n=100 arrangements with field sigmas between 1 and 10 bins and grid spacing in mm equal to 120σ; see Methods: *Simulated field arrangements*). Note that if fields overlap this analysis will tend to overestimate field size. LLS: linear least squares line fit. Text gives the result of a Pearson linear correlation and the slope of the LLS. **c**, Two-dimensional schematic showing the field spacing (scale or wavelength) estimation procedure. The median correlation found at different distances from the central peak were mapped and grid spacing was estimated as the location of the first peak after the central one. **d**, This analysis was validated on HCP, FCC and columnar (COL) field arrangements (n=100 each, random fields excluded as they do not have a reference spacing to validate against). Note that the size of our mazes limited field spacing to a maximum of approximately 120cm. LLS: linear least squares line fit. Text gives the result of a Pearson linear correlation and the slope of the LLS. **e**, The field spacing estimation results from real grid cells (mean and SEM across cells). For each cell the spacing was estimated as in **c**, shown here is the median correlation at every radius normalized by the first peak (the estimated spacing). A clear peak can be seen in both the arena (n=47, top) and lattice (n=27, bottom). Cells for which no spacing could be estimated are not shown.

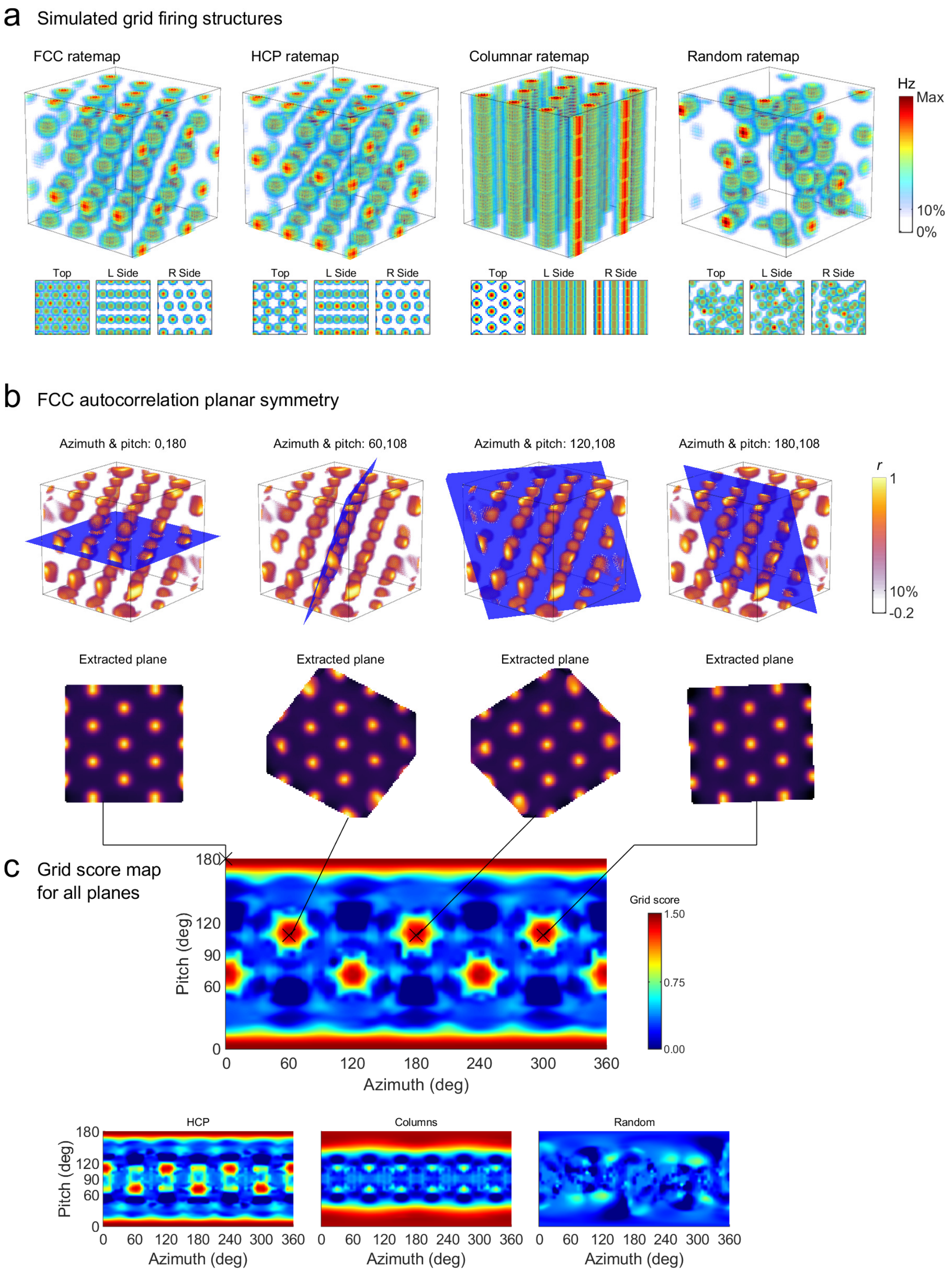

Fig. S5. Schematic demonstrating the planar symmetry analysis.

a, Example simulated field arrangements in oblique and side views. **b**, Taking the autocorrelation of the FCC arrangement shown in **a**, it is clear there are four planes that can be cut through the centre (top row) which slice through fields arranged in a planar hexagonal pattern (bottom row). **c**, We extract all possible planes and calculate the hexagonal grid score for each one. Mapping these according to their pitch/azimuth yields different patterns for the different field arrangements which can be used to differentiate them.

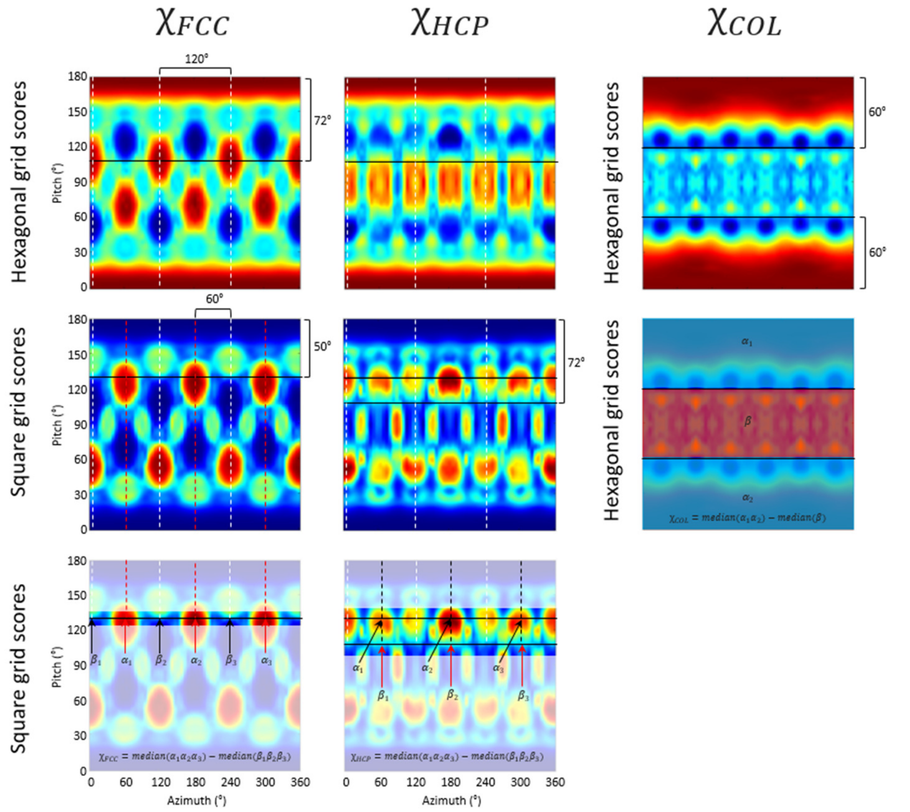

Fig. S6. Schematic showing how the structure scores (χ_{FCC}, χ_{HCP} & χ_{COL}) were calculated. After extracting all possible planes through a cell's spatial autocorrelation we calculated the hexagonal and square grid score for each one. Mapping these according to their pitch/azimuth yields different patterns for the different field arrangements which can be used to differentiate them. In each example the grid score maps are shown as cylindrical projections and have already been corrected so that the 'best plane' is horizontal (placing the highest hexagonal grid scores at the top and bottom of the maps). Left column demonstrates the process for the χ_{FCC} score: first, the three values at 72° from the best plane and separated by 120° between them with the maximum total sum were found. In an FCC arrangement, low square grid scores are found at 50° to the best plane at the same azimuthal angles and high square grid scores can be found offset 60° from them in azimuth. Thus, χ_{FCC} was calculated as the difference between these points. Middle column demonstrates the process for the χ_{HCP} score: again, the three values at 72° from the best plane and separated by 120° between them with the maximum total sum were found. In an HCP arrangement, high square grid scores are found at 50° to the best plane and offset 60° from them in azimuth, while low square grid scores are found at 72° to the best plane and at the same azimuth angles. Thus, χ_{HCP} was calculated as the difference between these points. Right column demonstrates the process for the χ_{COL} score: in a columnar arrangement there is a large field of pitch angles that intersect the columns and exhibit a high hexagonal grid score, however, there are virtually no high grid scores perpendicular to this plane. Thus, χ_{COL} was calculated as the difference between the median grid score found at the angles 0 - 60° & 120 - 180° and the median score found at the angles 60 to 120°. These angles were chosen as they divide the spherical domain into two regions with equal surface area (the poles and an equatorial 'belt').

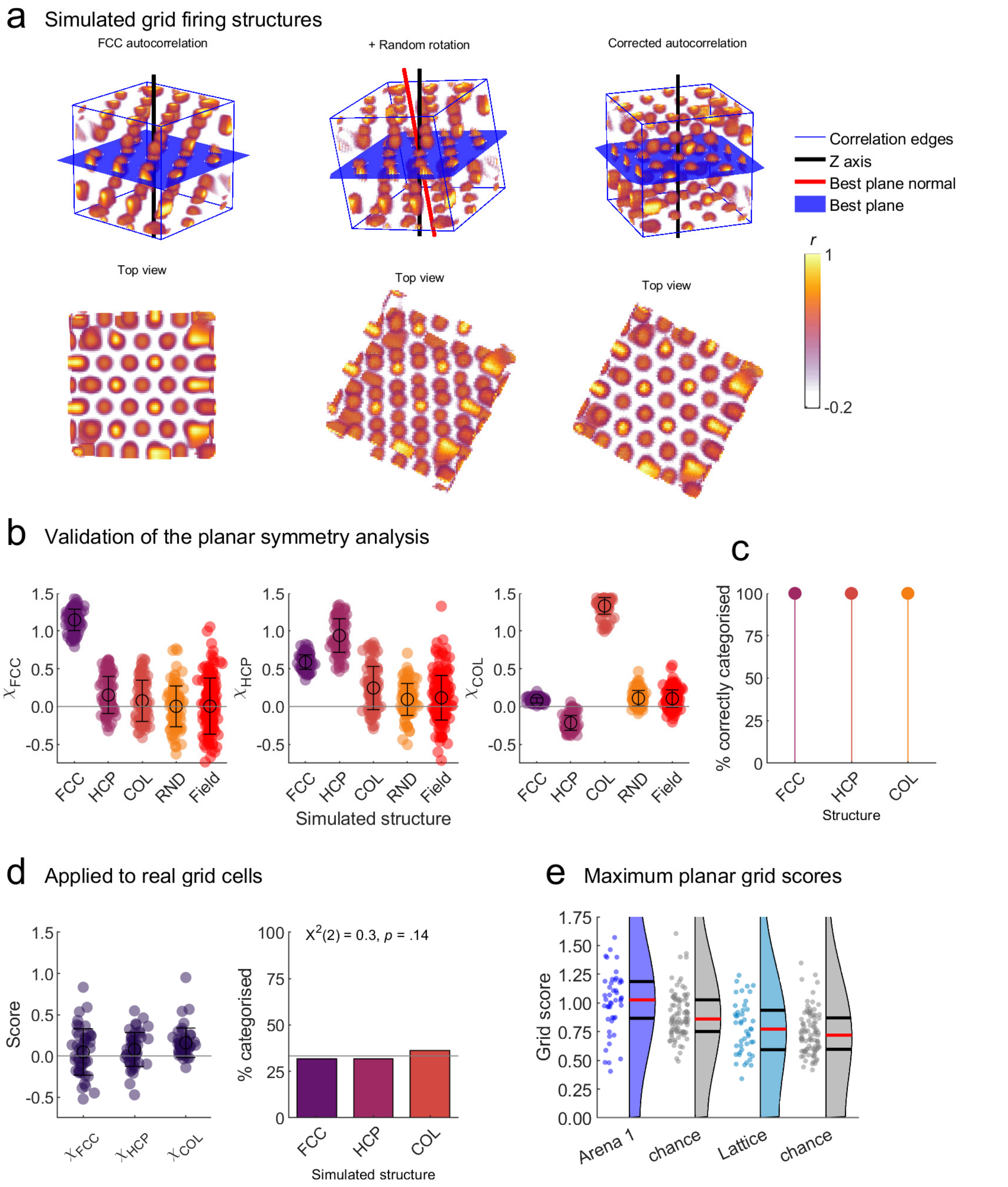

Fig. S7. Explanation and validation of the planar symmetry analysis.

For panels **b** & **d**: open circles denote mean, error bars denote SEM. **a**, Autocorrelations were randomly rotated before analysis by the planar symmetry method to avoid assumptions of the structure's orientation. This schematic shows the correct identification of the 'best plane' or the plane with the highest grid score and successful reorientation of the autocorrelation based on this. **b**, Filled markers represent simulated cells. Left) for every simulated field arrangement (n=100 simulated cells per arrangement) we calculated the three symmetry scores (χ_{FCC}, χ_{HCP} and χ_{COL}), in each case the correct score is highest for its corresponding field arrangement while the random ('RND') and shuffled field ('Field') arrangements are low in all three scores. **c**, If

simulated cells are categorized based on these scores (category = configuration with max score) they are grouped with 100% accuracy, confirming that the planar symmetry analysis can correctly identify field arrangements. **d**, Filled markers represent grid cells. Left) the three symmetry scores found for the real grid cells (n=46), all scores are much lower than expected if the arrangements were present and there was no significant difference between them ($F(2,138)$ = 2.9, p = .0584, η^2 = 0.040, one-way ANOVA). Right) if grid cells are categorized as in **c** an equivalent proportion of cells fall into each group meaning that no one field arrangement dominates the data. **e**, For each grid cell (n=47 & 46) we calculated the maximum possible planar grid score (the maximum value found in the hexagonal grid score maps in Fig. S6; blue markers and distributions) and compared these to shuffled arrangements of firing fields (n=2 per grid cell; Methods: *Grid field shuffle*; grey markers and distributions). As expected, arena grid scores were higher than chance (t(138) = 2.7, p = .0072, Cohen's d = 0.47). But lattice scores were not different from chance (t(135) = 0.9, p = .3668, Cohen's d = 0.16) suggesting that grid cells did not exhibit hexagonal arrangements of fields on any arbitrary plane through the lattice.

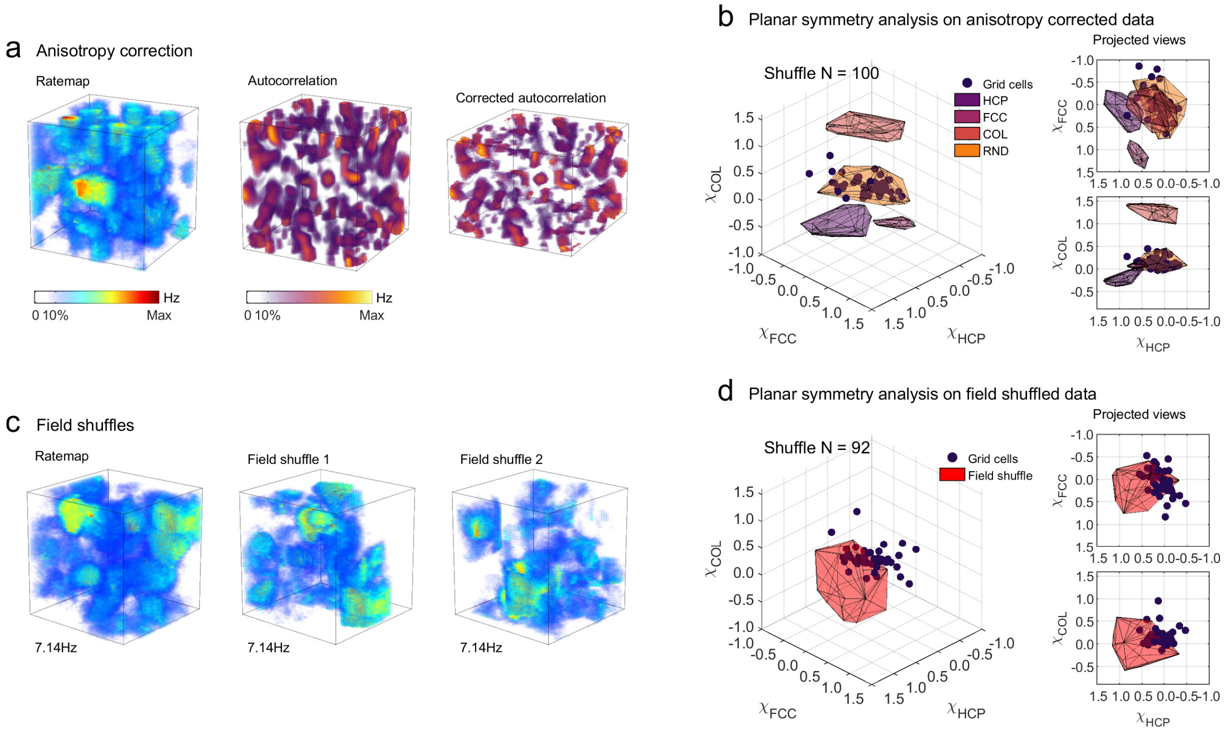

Fig. S8. Planar symmetry analysis yields similar results using a field shuffle

a, We repeated the planar symmetry analysis after correcting grid cell autocorrelations for anisotropy (Methods: *Autocorrelation correction for anisotropy*). An example of a grid cell ratemap is shown (left), the autocorrelation of this ratemap (middle) and the same autocorrelation after correcting for field anisotropy (right). Note that the corrected autocorrelation is compressed along the vertical axis so that the central peak is more spherical than in the uncorrected version. **b**, The configuration results when using these autocorrelations did not differ from uncorrected ones. n=46 cells. **c**, In addition to the FCC, HCP, columnar and random field arrangements described elsewhere we also used a grid field shuffle which randomly shuffles field positions while maintaining their local firing activity as best as possible. Owing to the computational demands of this analysis we performed the shuffle only twice per grid cell. An example of a grid cell ratemap is shown on the left and two versions of the same ratemap with shuffled firing fields on the right. **d**, The ratemaps in this shuffled dataset exhibit some higher FCC and HCP scores (n=92 shuffles, red polygon) than the real grid cells (n=46, black markers). However, on average the scores are all centred on zero, like the real grid cells (values shown in Fig. S7B, ‘Field’ group).

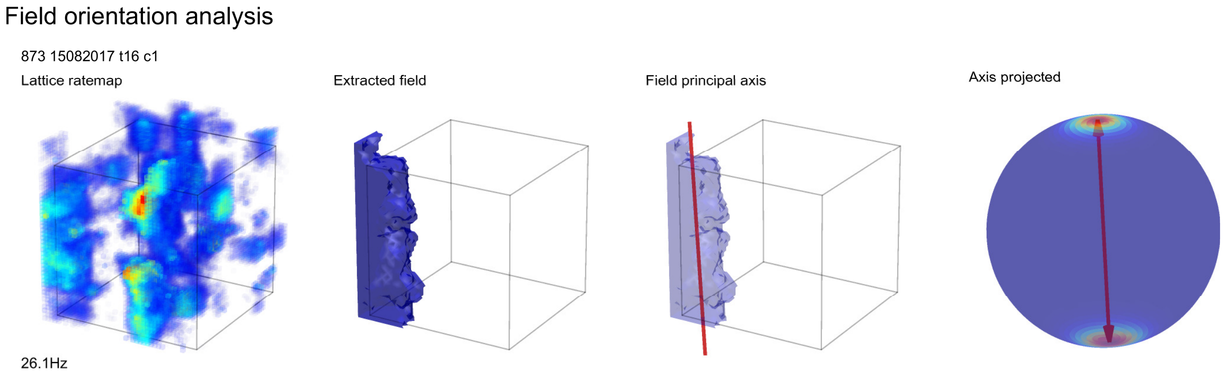

Fig. S9. Schematic demonstrating the field orientation analysis.

An example firing rate map (left) is thresholded at 20% of the peak firing rate and regions which passed our criteria were considered place fields (2nd plot shows an example field). We can visualize these regions (3rd plot) and extract features such as their principal axis (red line). To visualize the orientation of multiple fields we project these axes onto a unit sphere and generate a spherical Von-Mises kernel smoothed density map (right plot), where hot colours denote that many fields ‘pointed’ their principal axis in this orientation.

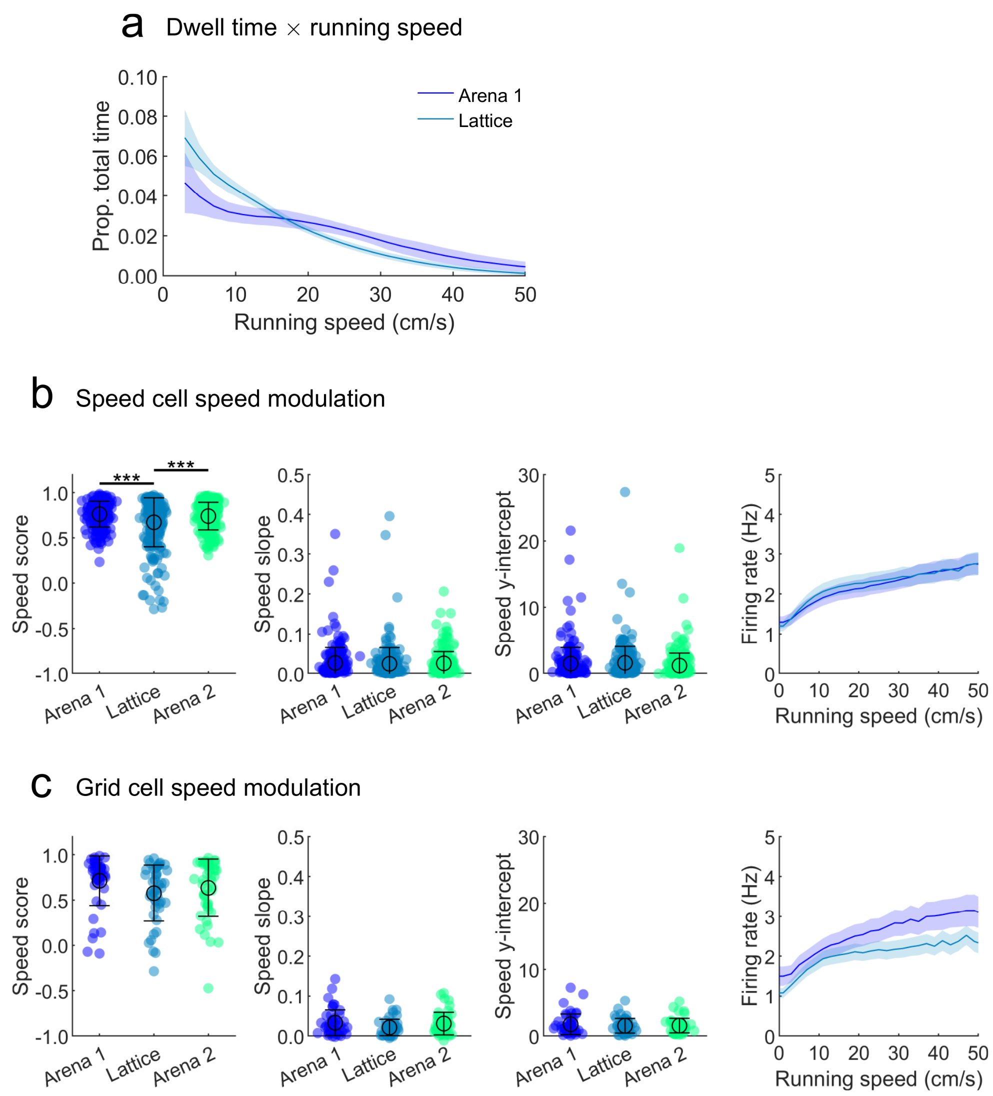

Fig. S10. Very little change in speed cell activity and speed coding by grid cells in the lattice.

For panels **b** & **c**: open circles denote mean, error bars denote SEM. **a**, The mean and SEM proportion of time animals spent moving at various running speeds, averaged across sessions. **b**, Speed scores of speed modulated non-grid cells (n=222) dropped in the lattice ($F(2,663) = 12.6$, $p = 4.13 \times 10^{-6}$, $\eta^2 = 0.0367$, one-way ANOVA), however, the slope and y-intercept of speed-firing rate relationships were unaffected ($p = .8047$ & $p = .1418$, respective one-way ANOVAs). Right plot shows the mean and SEM running speed vs firing rate relationship for all speed cells. **c**, Speed scores, slope and y-intercepts of speed-firing rate relationships were also unaffected in grid cells (n=47, 46 & 47; $p = .0921$, $p = .0895$ & $p = .6482$, respective one-way ANOVAs). Right plot shows the mean and SEM running speed vs firing rate relationship for all grid cells.

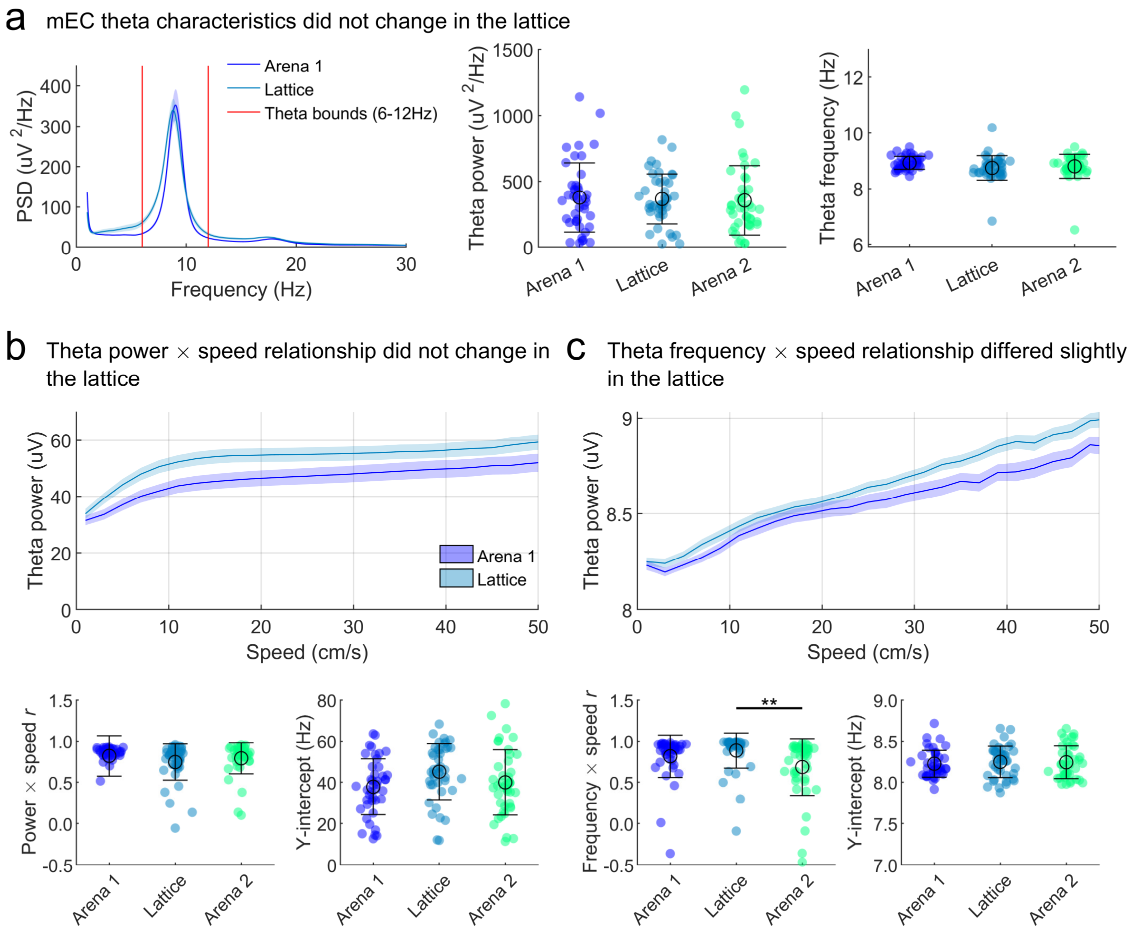

Fig. S11. Very little change in theta power and frequency or relationship with running speed in the lattice.

For all panels n=42 sessions. For panels **a** (right), **b** (bottom) & **c** (bottom): Filled markers represent sessions, open circles denote mean, error bars denote SEM. Markers represent sessions. Averages and errors computed across sessions. **a**, Left) the mean PSD in each maze, averaged across sessions. Red lines denote the frequency band associated with theta rhythm. Middle) maximum power associated with the theta band in each session, these did not differ ($F(2,123) = 0.1$, $p = .9180$, $\eta^2 = 0.0014$, one-way ANOVA). Right) frequency associated with max theta power in each maze session, these did not differ ($F(2,123) = 2.5$, $p = .0828$, $\eta^2 = 0.0397$). **b**, Mean and SEM theta power (amplitude of the Hilbert transform) observed at various running speeds, averaged across sessions. Below this are boxplots where markers represent sessions. These show the Pearson's correlation between power and speed which did not differ between mazes (left; $F(2,123) = 1.2$, $p = .2957$, $\eta^2 = 0.0196$, one-way ANOVA) and the y-intercept of the power and speed relationship which also did not differ (right; $F(2,123) = 2.9$, $p = .0602$, $\eta^2 = 0.0447$, one-way ANOVA). **c**, Same as **b** but for theta frequency. The correlation between theta frequency and speed decreased in the second arena session with respect to the lattice ($F(2,123) = 5.6$, $p = .0046$, $\eta^2 = 0.0839$, one-way ANOVA) while the y-intercept did not differ (right; $F(2,123) = 2.9$, $p = .8367$, $\eta^2 = 0.0029$, one-way ANOVA).

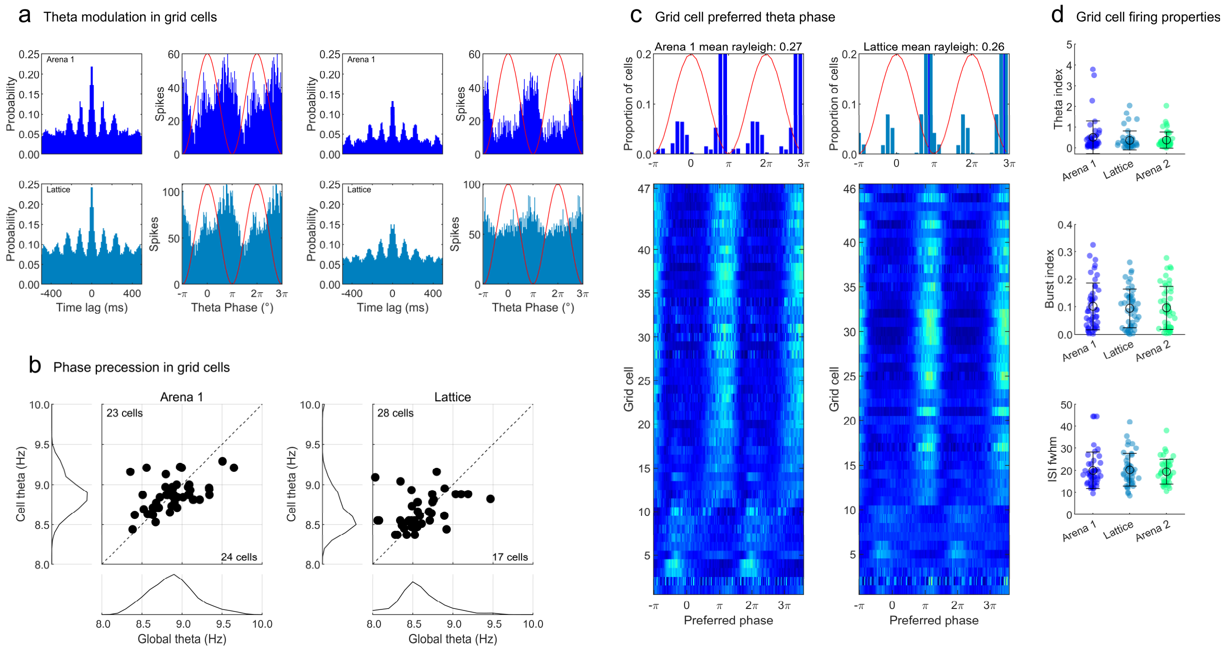

Fig. S12. Grid cell spiking dynamics were preserved in the lattice maze.

a, Spike probability properties of two example grid cells. Left four plots are for one cell, right four plots are a different cell. Top row gives data for the first arena, bottom row gives data for the lattice. For each maze the left plot shows the 400ms spike autocorrelogram (1ms bins) and the right plot shows the spike-theta phase histogram. Red lines trace the amplitude of a scaled theta wave at every phase. Theta modulation and phase preference were unaffected in the lattice. **b**, Scatter plots showing the frequency of intrinsic theta (extracted from the spike autocorrelograms) vs extrinsic theta (extracted from the power spectral density estimate of the LFP). Markers represent grid cells, numbers represent counts that are above or below the diagonal. Markers above the dashed line represent cells firing at a rate faster than global theta, an indicator of phase precession. Cells were not modulated at a higher frequency than extrinsic theta in the arena (n=47, $\chi^2(1) = 0.02$, $p = .8840$) or lattice (n=45, $\chi^2(1) = 2.69$, $p = .1011$, Chi-square tests of expected proportions). **c**, Histograms showing the preferred theta phase (value of circular mean) of all grid cells. Red lines trace the amplitude of a scaled theta wave at every phase, blue vertical lines show the circular mean of the preferred phases. Cells showed an equal preference for one theta phase in both mazes ($F(1,92) = 0.1$, $p = .7067$, $\eta^2 = 0.0016$, one-way ANOVA comparing spike phase Rayleigh vector lengths) and these preferred phases did not differ between the mazes (k = 423, $p > .99$, two-sample Kuiper test). Shown below each plot is the firing probability of all grid cells relative to theta phase. Each row represents a cell and rows are sorted from top to bottom by the circular mean of the cell's phase angles from early to late. **d**, Filled markers represent cells, open circles denote mean, error bars denote SEM. General firing properties such as the theta index extracted from each grid cell's (n=47, 46 & 47) spike autocorrelation (top; $F(2,135) = 0.9$, $p = .3905$, $\eta^2 = 0.0138$, one-way ANOVA), burst index (middle; $F(2,137) = 0.1$, $p = .9025$, $\eta^2 = 0.0015$, one-way ANOVA) and the full width at half maximum of the interspike interval (fwhm of the ISI: bottom; $F(2,130) = 0.1$, $p = .8616$, $\eta^2 = 0.0023$, one-way ANOVA) were unchanged in the lattice maze.

Table S1. Sessions cell statistics for each rat

	Rat	Sessions	Cells	Grid cells (% cells)	Directional cells (% cells)	Speed cells (% cells)	mEC layer
	872*	2	65	0 (0.0)	0 (0.0)	9 (4.0)	III/IV
	873[a]	3	82	8 (9.8)	0 (0.0)	25 (11.3)	IV
	874	4	64	5 (7.8)	3 (7.5)	20 (9.0)	II/III
	895[a]	2	48	1 (2.1)	1 (2.5)	21 (9.5)	III
	918	5	26	5 (19.2)	19 (47.5)	14 (6.3)	III
	922	10	97	7 (7.2)	10 (25.0)	47 (21.2)	III
	FEZ*	3	68	0 (0.0)	0 (0.0)	7 (3.2)	I/II
	ONE	5	70	6 (8.6)	0 (0.0)	32 (14.4)	III/IV
	ZAX	8	105	15 (14.3)	7 (17.5)	47 (21.2)	III
Total	9	42	625	47 (7.5)	40 (6.4)	222 (35.5)	-

Session and cell totals for each rat. Histological verification of the mEC layers can be seen in Fig. S2.

*Note that two rats did not yield any grid cells but contributed to the behaviour and other electrophysiological data.

[a]Rats 873 and 895 exhibited horizontal planar grid firing patterns.
