# Supplementary material for: Irregular distribution of grid cell firing fields in rats exploring a 3D volumetric space
Source: Nat Neurosci. 2021 Aug 11;24(11):1567–73. doi: 10.1038/s41593-021-00907-4 (PMC8553607; doi:10.1038/s41593-021-00907-4)
Supplement: Supplementary file 2 — Reporting Summary [file 41593_2021_907_MOESM2_ESM.pdf]

nature research

Corresponding author(s): Roddy Grieves
Kate Jeffery

Last updated by author(s): Jul 8, 2021

Reporting Summary

Nature Research wishes to improve the reproducibility of the work that we publish. This form provides structure for consistency and transparency in reporting. For further information on Nature Research policies, see our Editorial Policies and the Editorial Policy Checklist.

Statistics

For all statistical analyses, confirm that the following items are present in the figure legend, table legend, main text, or Methods section.

n/a	Confirmed	
☐	☒	The exact sample size (*n*) for each experimental group/condition, given as a discrete number and unit of measurement
☐	☒	A statement on whether measurements were taken from distinct samples or whether the same sample was measured repeatedly
☐	☒	The statistical test(s) used AND whether they are one- or two-sided<br>*Only common tests should be described solely by name; describe more complex techniques in the Methods section.*
☐	☒	A description of all covariates tested
☐	☒	A description of any assumptions or corrections, such as tests of normality and adjustment for multiple comparisons
☐	☒	A full description of the statistical parameters including central tendency (e.g. means) or other basic estimates (e.g. regression coefficient) AND variation (e.g. standard deviation) or associated estimates of uncertainty (e.g. confidence intervals)
☐	☒	For null hypothesis testing, the test statistic (e.g. F, t, r) with confidence intervals, effect sizes, degrees of freedom and P value noted<br>*Give P values as exact values whenever suitable.*
☒	☐	For Bayesian analysis, information on the choice of priors and Markov chain Monte Carlo settings
☒	☐	For hierarchical and complex designs, identification of the appropriate level for tests and full reporting of outcomes
☐	☒	Estimates of effect sizes (e.g. Cohen's *d*, Pearson's *r*), indicating how they were calculated

Our web collection on statistics for biologists contains articles on many of the points above.

Software and code

Policy information about availability of computer code

Data collection	Descriptions of software are given in text. Mainly; dacqUSB (Axona), dacqTrack (Axona)
Data analysis	Descriptions of software are given in text. Mainly; Tint (v.4.4.12, Axona), Matlab (2020a, The Mathworks), Klustakwik v3, Chronux toolbox plugin for Matlab (2.12 v03, http://chronux.org), CircStat circular statistics toolbox for Matlab (Mathworks file exchange; Berens, P.), smoothn n-dimensional interpolation algorithm (Mathworks file exchange; Garcia, D.). All custom Matlab codes are available for downloaded here: DOI: 10.17632/s3h7n7jyr4.1.

For manuscripts utilizing custom algorithms or software that are central to the research but not yet described in published literature, software must be made available to editors and reviewers. We strongly encourage code deposition in a community repository (e.g. GitHub). See the Nature Research guidelines for submitting code & software for further information.

Data

Policy information about availability of data

All manuscripts must include a data availability statement. This statement should provide the following information, where applicable:

- Accession codes, unique identifiers, or web links for publicly available datasets
- A list of figures that have associated raw data
- A description of any restrictions on data availability

The full data set, in its analyzed Matlab compatible format, is available here: DOI: 10.17632/s3h7n7jyr4.1; this includes all of the data reported or presented in the main text or supplementary files and everything needed to recreate the main analyses and figures. It also includes all tracking data, spike waveforms and spike time information. The raw data files are too large to be made publicly available (150GB) and because the only benefit they offer is the ability to recluster the raw spike data (something which researchers are unlikely to want or need) these data are instead available from the authors on request.

Field-specific reporting

Please select the one below that is the best fit for your research. If you are not sure, read the appropriate sections before making your selection.

☒ Life sciences ☐ Behavioural & social sciences ☐ Ecological, evolutionary & environmental sciences

For a reference copy of the document with all sections, see nature.com/documents/nr-reporting-summary-flat.pdf

Life sciences study design

All studies must disclose on these points even when the disclosure is negative.

Sample size	Sample sizes of 4-10 animals are typical in hippocampal research (Grieves et al. 2020; Flores-Abreu et al. 2014; Porter et al. 2018). We collected data from 9 animals (grid cells in 7 of these) which is a large enough number to ensure the effects we see are not specific to one animal or cohort. Cell yields can vary dramatically in hippocampal recordings, we recorded 115 grid cells of which we selected only the most stable, leaving 47 grid cells. This number is in line with other research using wireless telemetry in animals navigating in 3D (i.e. 55 place cells in flying bats: Yartsev & Ulanovsky, 2013; 44 grid cells in climbing rats: Hayman et al. 2015; 19 head direction cells in climbing rats: Page et al., 2018). We feel this yield is high given the difficulty of the wireless and 3D nature of the recordings. No a priori power analysis was undertaken.
Data exclusions	No animals or grid cells were excluded. Data clusters that were not classified as grid cells were excluded.
Replication	Replication was not undertaken in this study although our effects are present across animals and sessions. Replication of our effects has to be performed by an independent laboratory and there are not currently laboratories with the required equipment to carry out this replication.
Randomization	We had one treatment group so there was no need for randomization.
Blinding	All animals were part of the same treatment group (arena-lattice-arena) and so the only blinding possible would be against the maze type. However, blinding was not possible during data collection as the difference in mazes was immediately apparent. Furthermore, there was no way for the experimenter to influence the outcome of the neural recordings in a predictable manner. The same experimenters who collected the data analyzed it and were thus not able to be blinded during analysis; furthermore, it would also not be possible for the analysis to be performed blinded as the two mazes vary in their dimensions and shape making their data often immediately recognizable.

Reporting for specific materials, systems and methods

We require information from authors about some types of materials, experimental systems and methods used in many studies. Here, indicate whether each material, system or method listed is relevant to your study. If you are not sure if a list item applies to your research, read the appropriate section before selecting a response.

Materials & experimental systems

n/a	Involved in the study	
☒	☐	Antibodies
☒	☐	Eukaryotic cell lines
☒	☐	Palaeontology and archaeology
☐	☒	Animals and other organisms
☒	☐	Human research participants
☒	☐	Clinical data
☒	☐	Dual use research of concern

Methods

n/a	Involved in the study	
☒	☐	ChIP-seq
☒	☐	Flow cytometry
☒	☐	MRI-based neuroimaging

Animals and other organisms

Policy information about studies involving animals; ARRIVE guidelines recommended for reporting animal research

Laboratory animals	Lister hooded rats, male, 250-400g in weight, age unknown (estimated to be between 10 and 20 weeks).
Wild animals	The study did not involve wild animals
Field-collected samples	The study did not involve samples taken from the field/wild.
Ethics oversight	Animal care technicians, Named Veterinary Surgeons and Named Animal Care and Welfare Officers provided ethical and care guidance. This experiment complied with the national [Animals (Scientific Procedures) Act, 1986, United Kingdom] and international [European Communities Council Directive of November 24, 1986 (86/609/EEC)] legislation governing the maintenance of laboratory animals and their use in scientific experiments. Experimental procedures were approved by the UK Home Office and ethical approval was granted through consultation with veterinary staff at University College London.

Note that full information on the approval of the study protocol must also be provided in the manuscript.
