# Supplementary figures and images for: Irregular distribution of grid cell firing fields in rats exploring a 3D volumetric space
Source: Nat Neurosci. 2021 Aug 11;24(11):1567–73. doi: 10.1038/s41593-021-00907-4 (PMC8553607; doi:10.1038/s41593-021-00907-4)

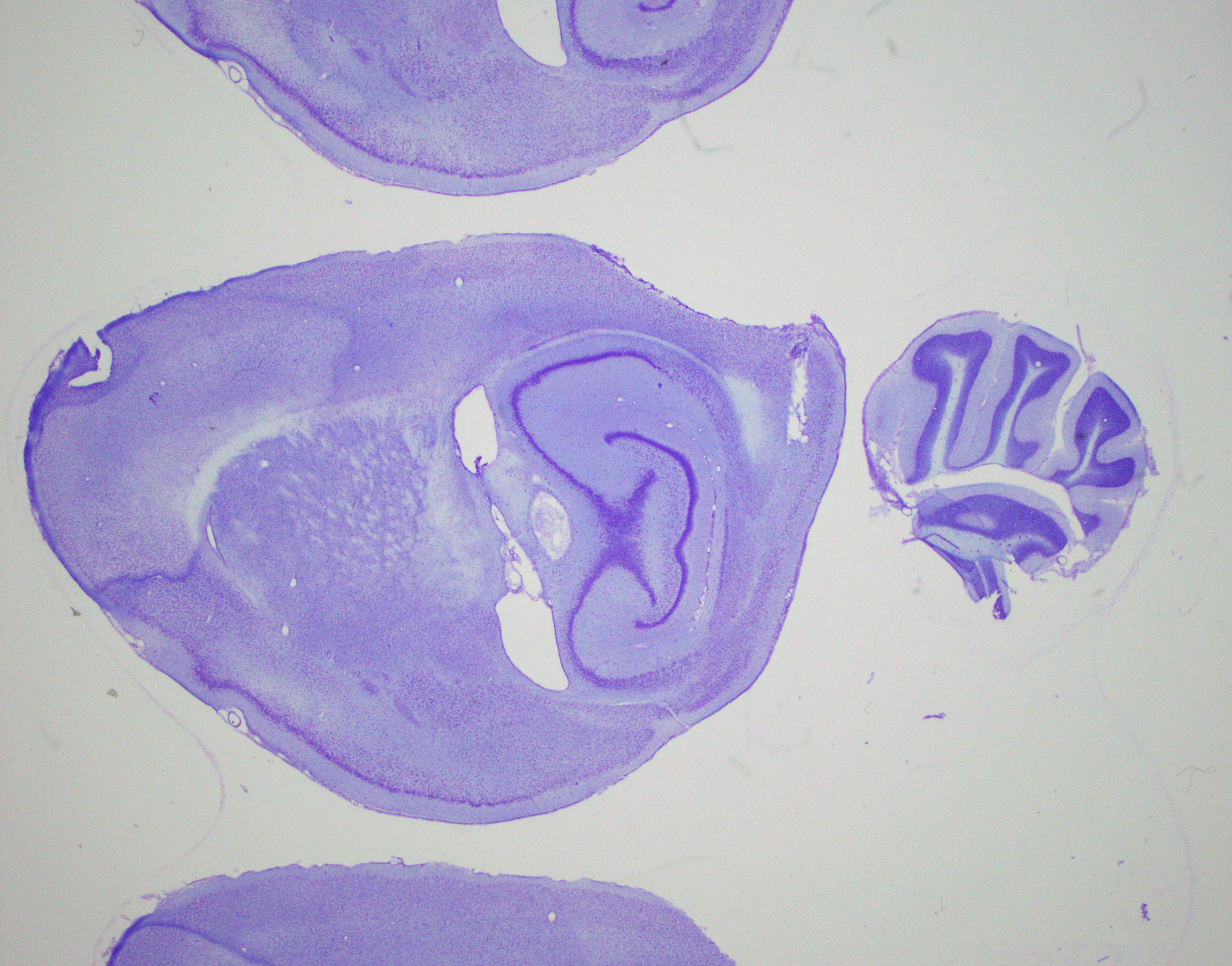

Supplement: Source Data Fig. 1 — Unprocessed histology images. [file 41593_2021_907_MOESM4_ESM.zip › 872P5 1.TIF]

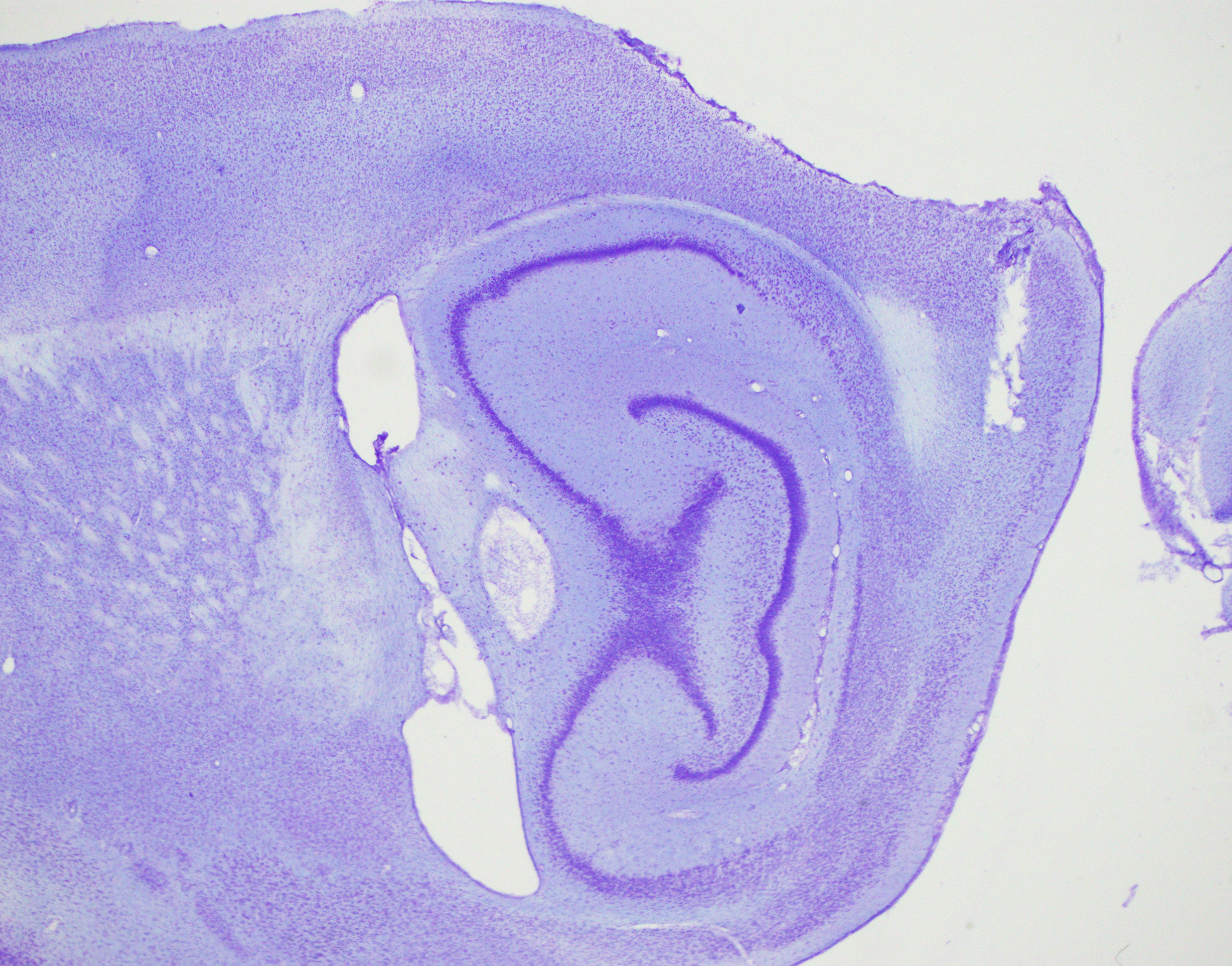

Supplement: Source Data Fig. 1 — Unprocessed histology images. [file 41593_2021_907_MOESM4_ESM.zip › 872P5.TIF]

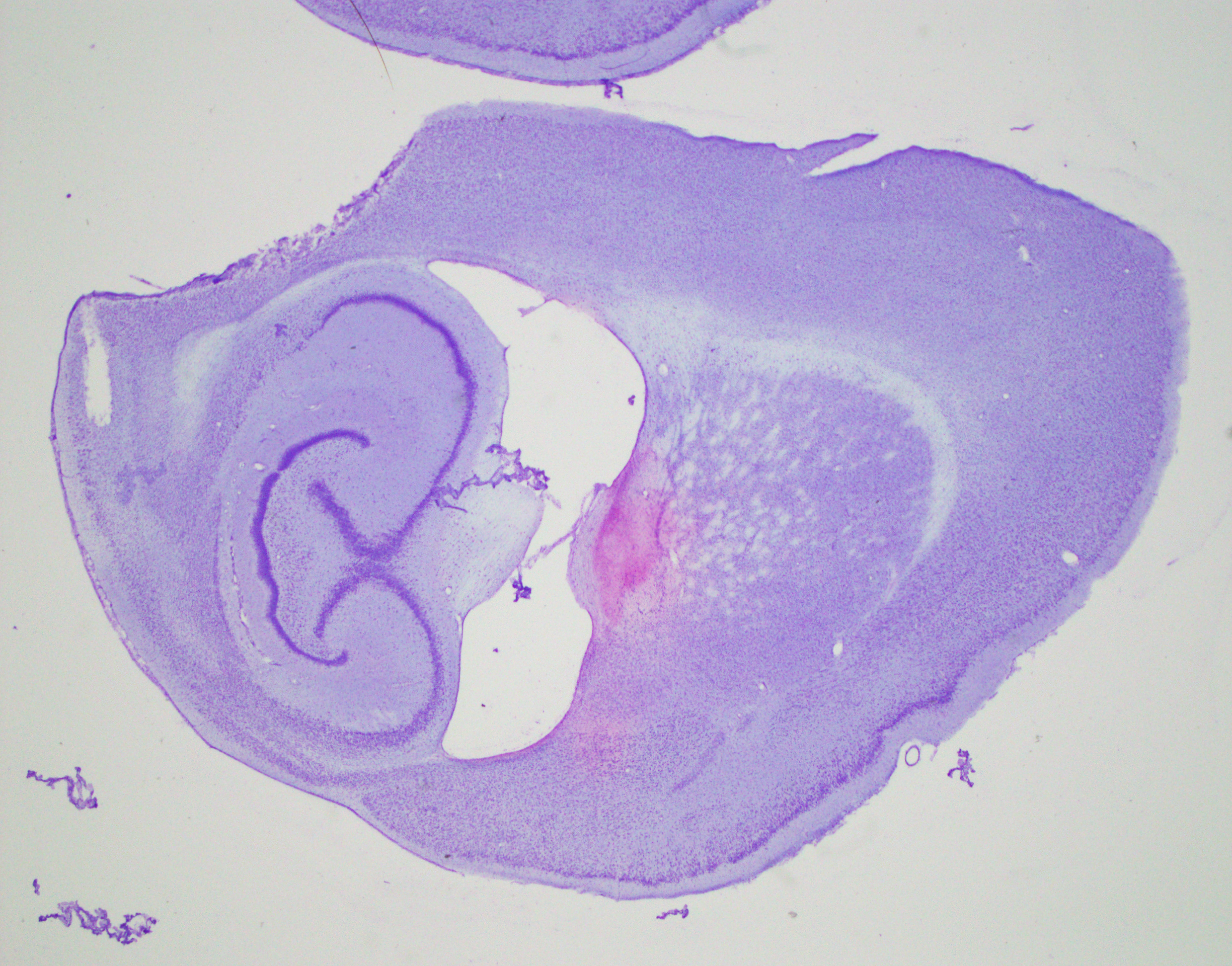

Supplement: Source Data Fig. 1 — Unprocessed histology images. [file 41593_2021_907_MOESM4_ESM.zip › 873P6 1.TIF]

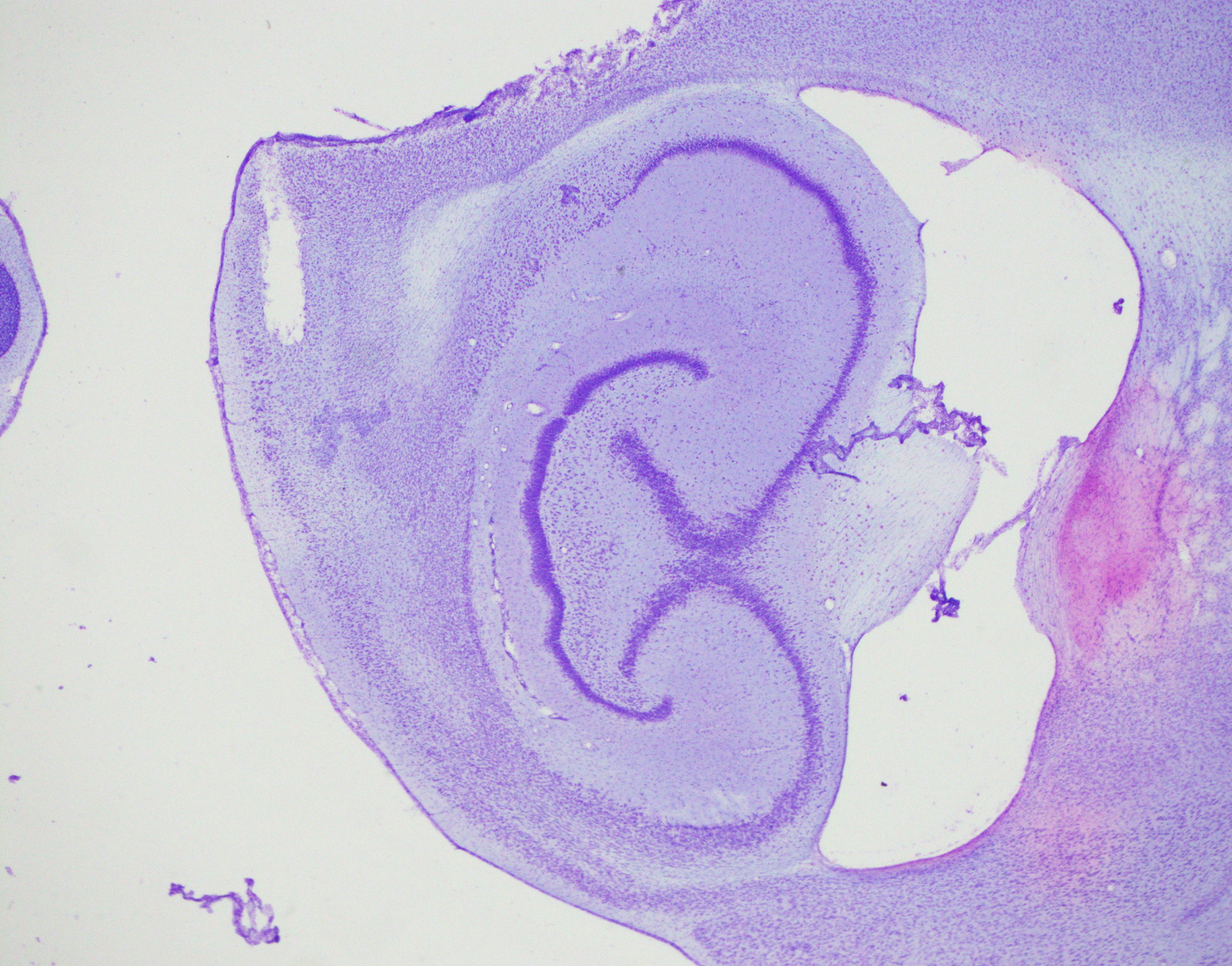

Supplement: Source Data Fig. 1 — Unprocessed histology images. [file 41593_2021_907_MOESM4_ESM.zip › 873P6 2.TIF]

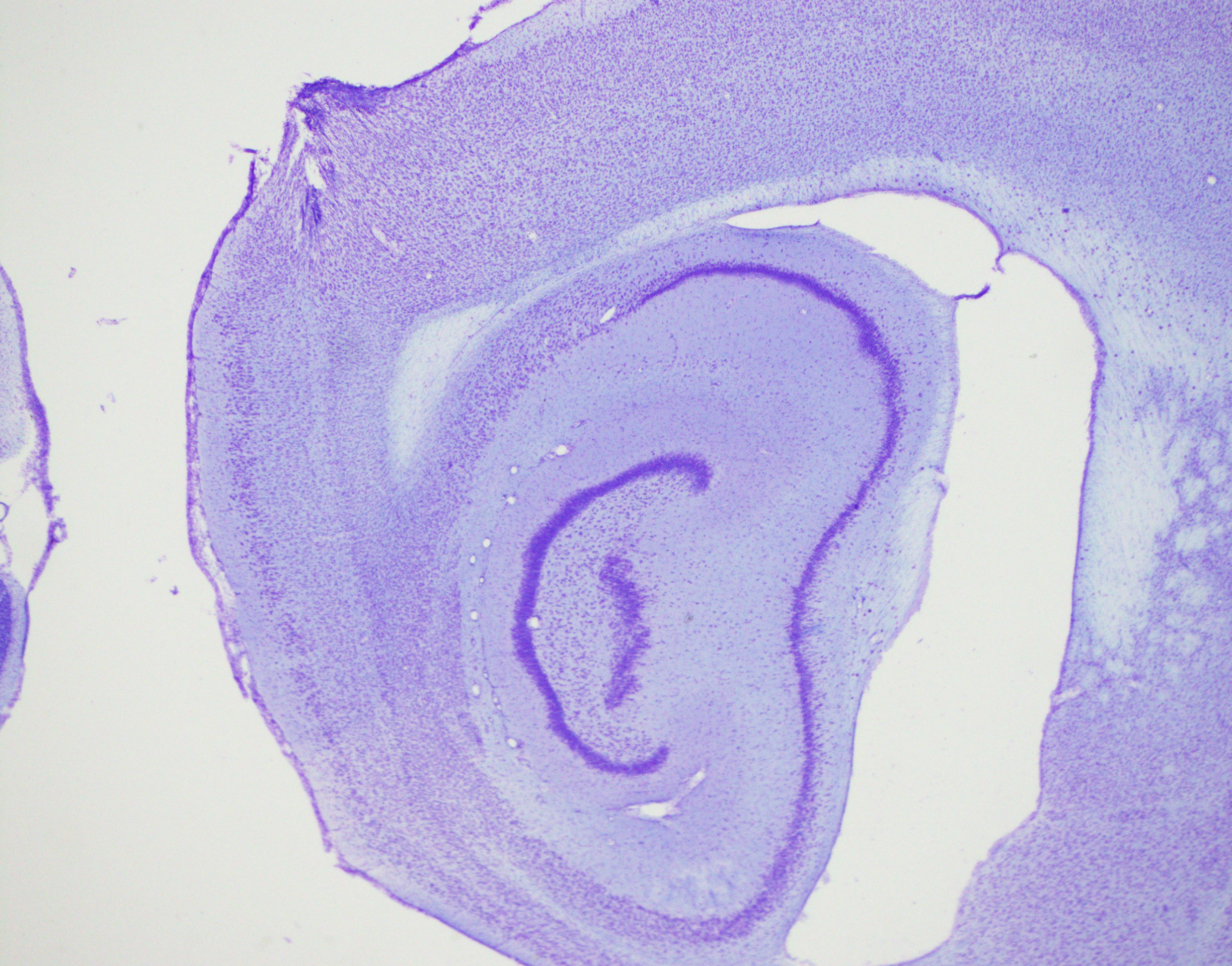

Supplement: Source Data Fig. 1 — Unprocessed histology images. [file 41593_2021_907_MOESM4_ESM.zip › 874 P7 2.TIF]

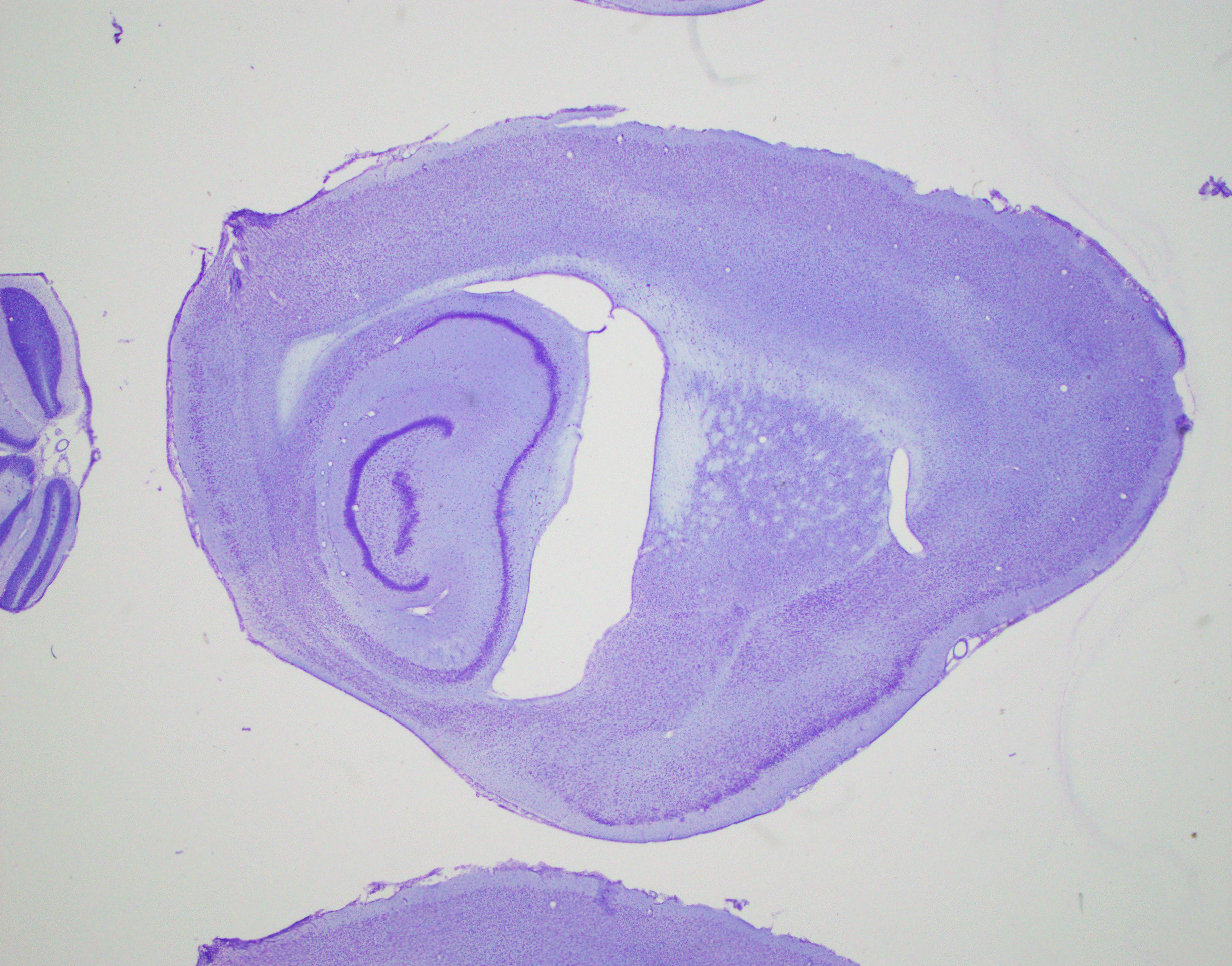

Supplement: Source Data Fig. 1 — Unprocessed histology images. [file 41593_2021_907_MOESM4_ESM.zip › 874P7 1.TIF]

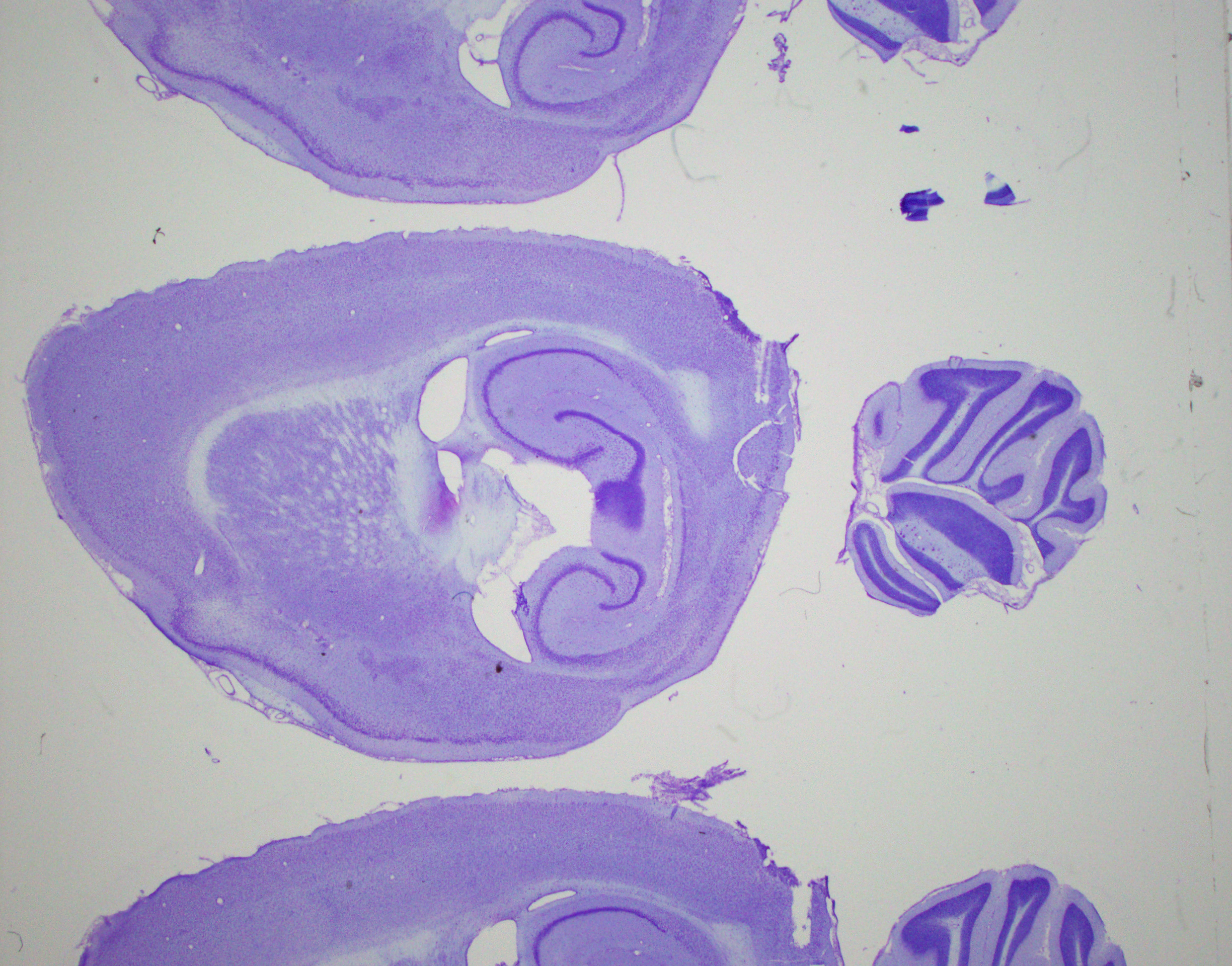

Supplement: Source Data Fig. 1 — Unprocessed histology images. [file 41593_2021_907_MOESM4_ESM.zip › 895P10 1.TIF]

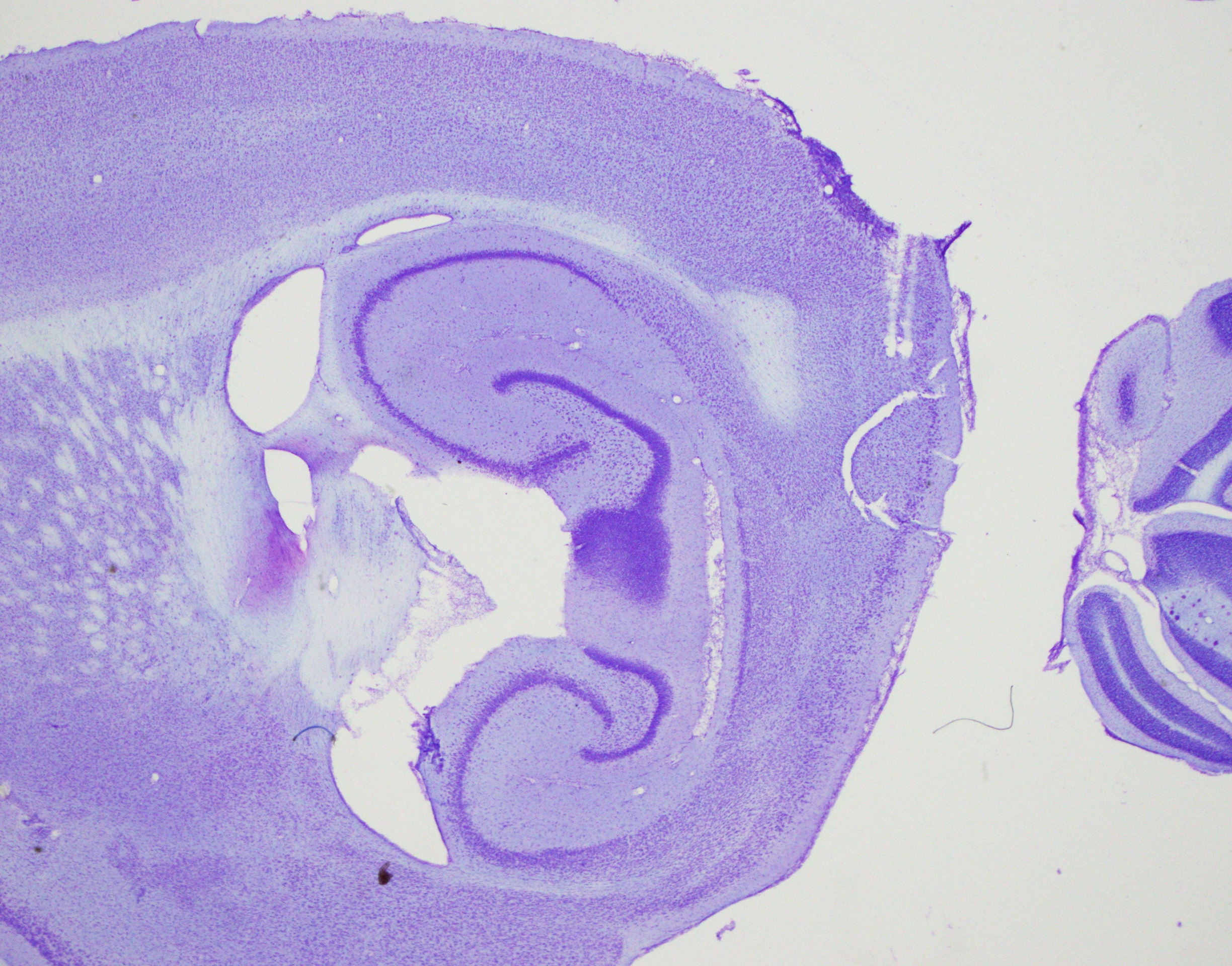

Supplement: Source Data Fig. 1 — Unprocessed histology images. [file 41593_2021_907_MOESM4_ESM.zip › 895P10 2.TIF]

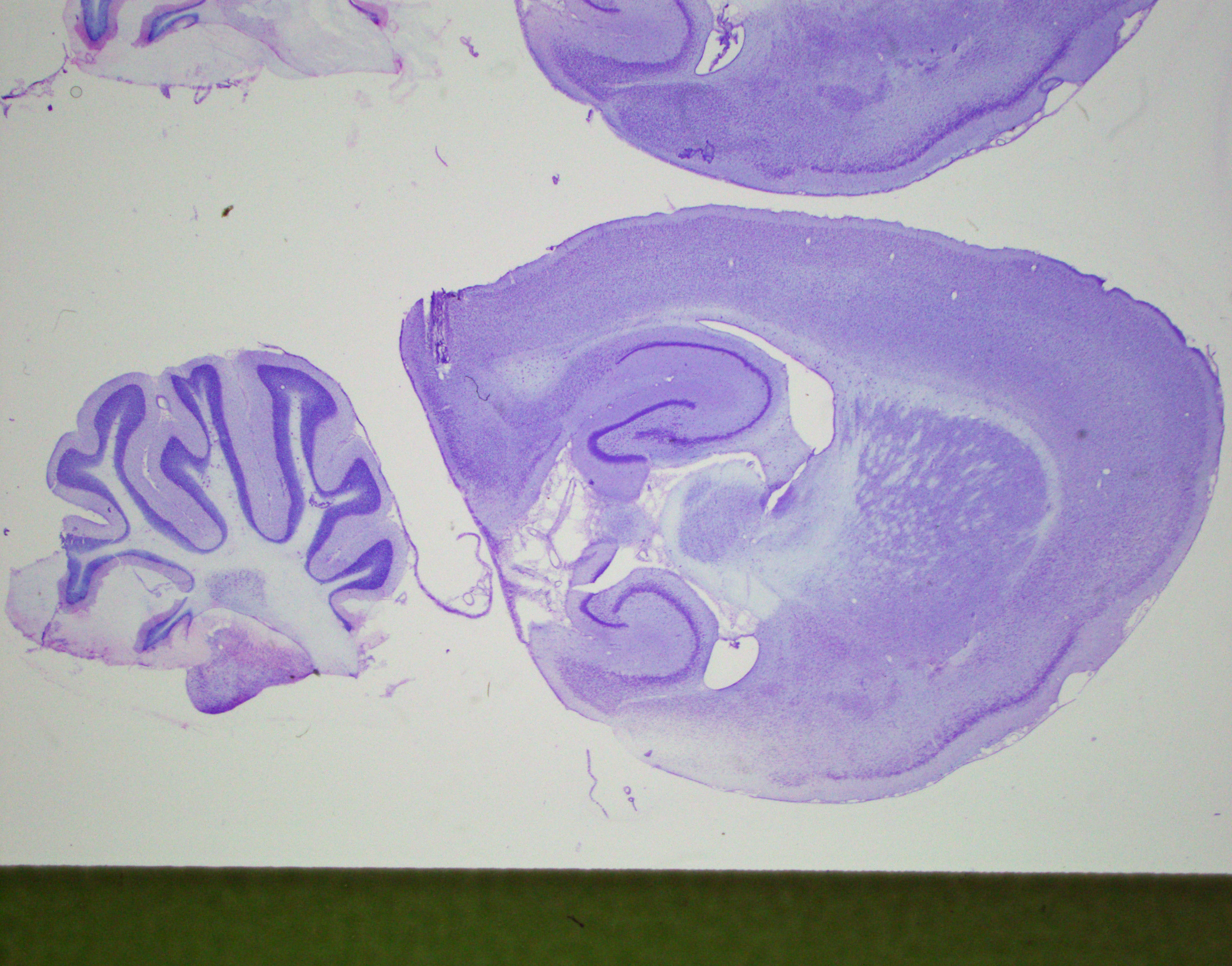

Supplement: Source Data Fig. 1 — Unprocessed histology images. [file 41593_2021_907_MOESM4_ESM.zip › 918P14 1.TIF]

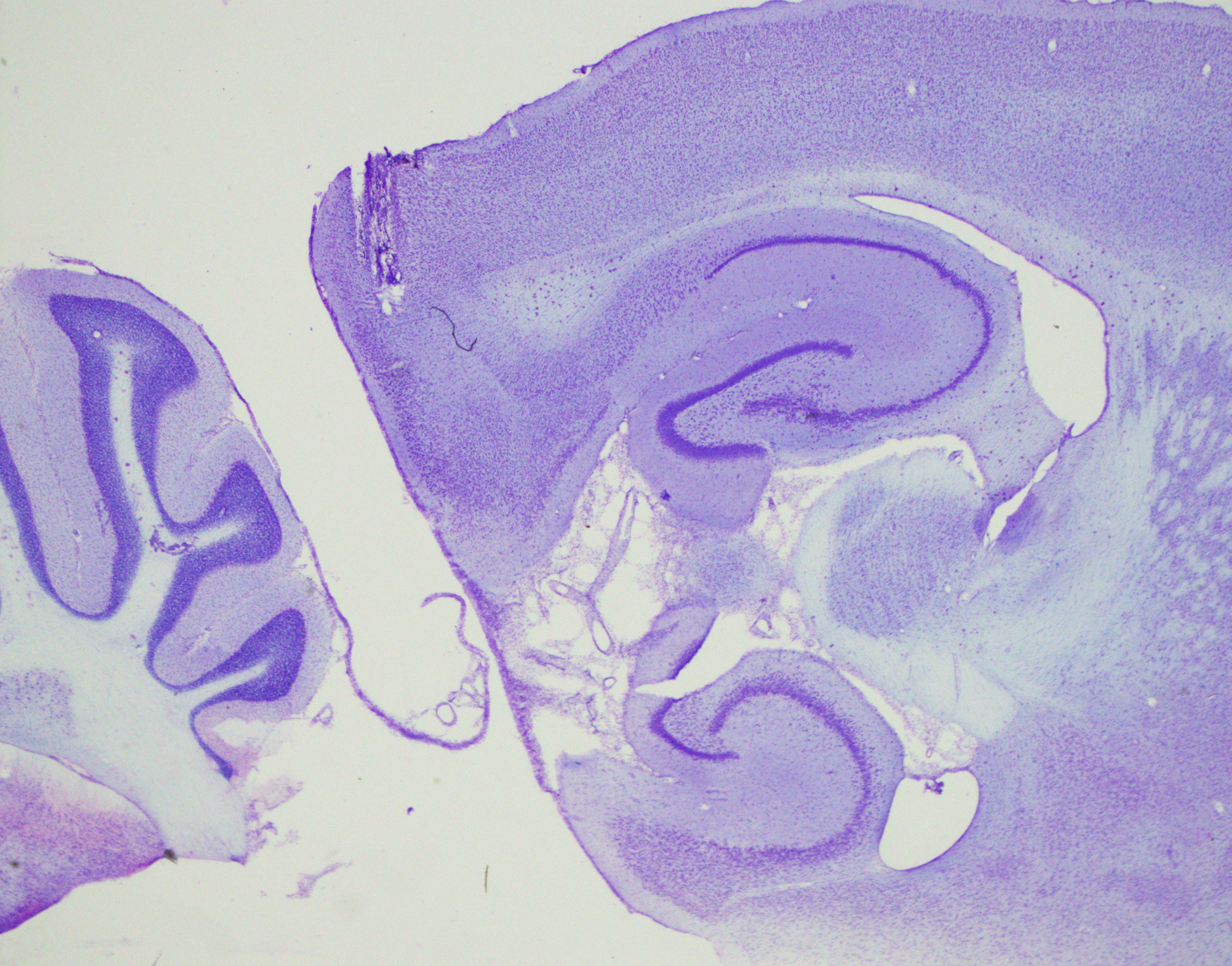

Supplement: Source Data Fig. 1 — Unprocessed histology images. [file 41593_2021_907_MOESM4_ESM.zip › 918P14 2.TIF]

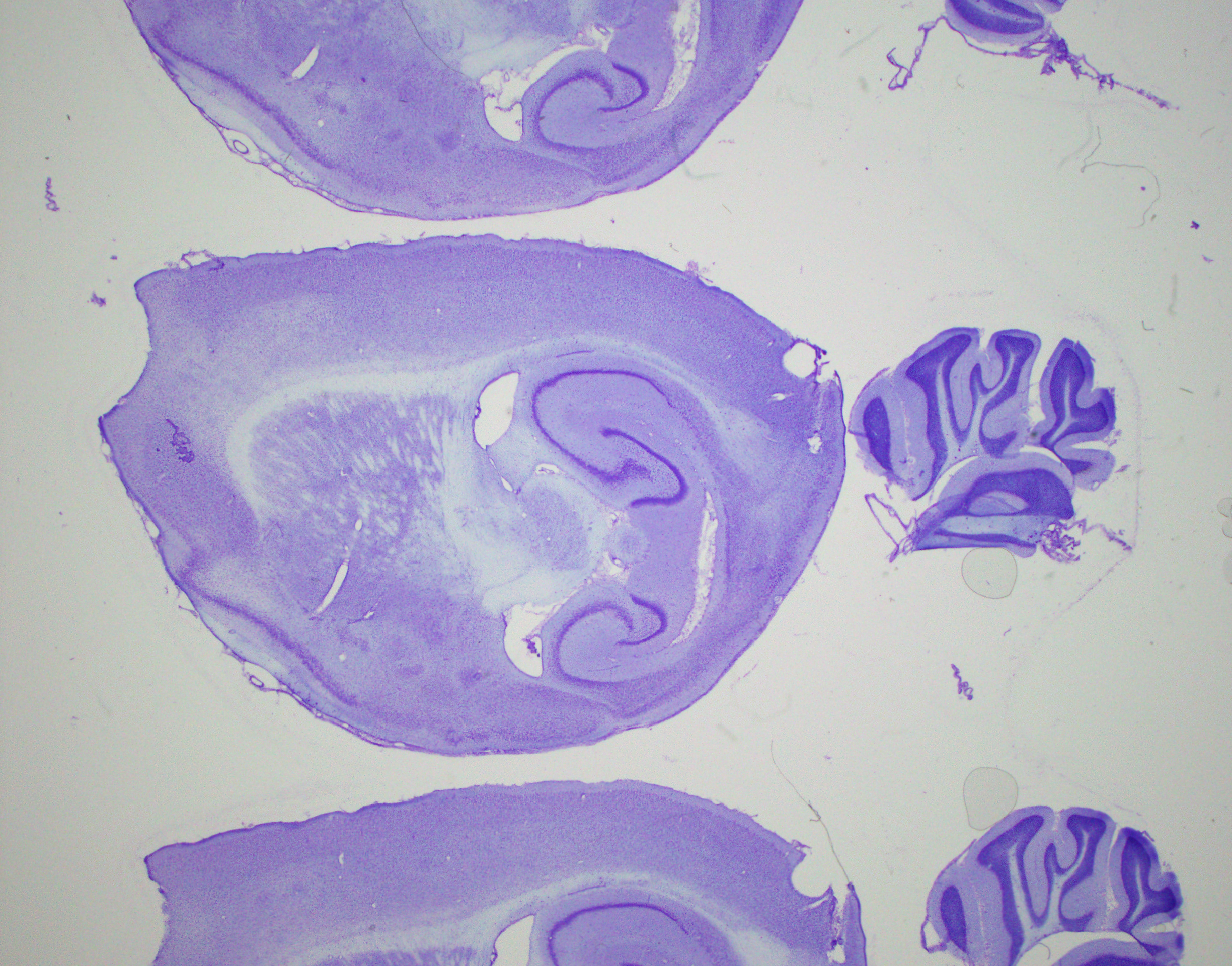

Supplement: Source Data Fig. 1 — Unprocessed histology images. [file 41593_2021_907_MOESM4_ESM.zip › 922P15 1.TIF]

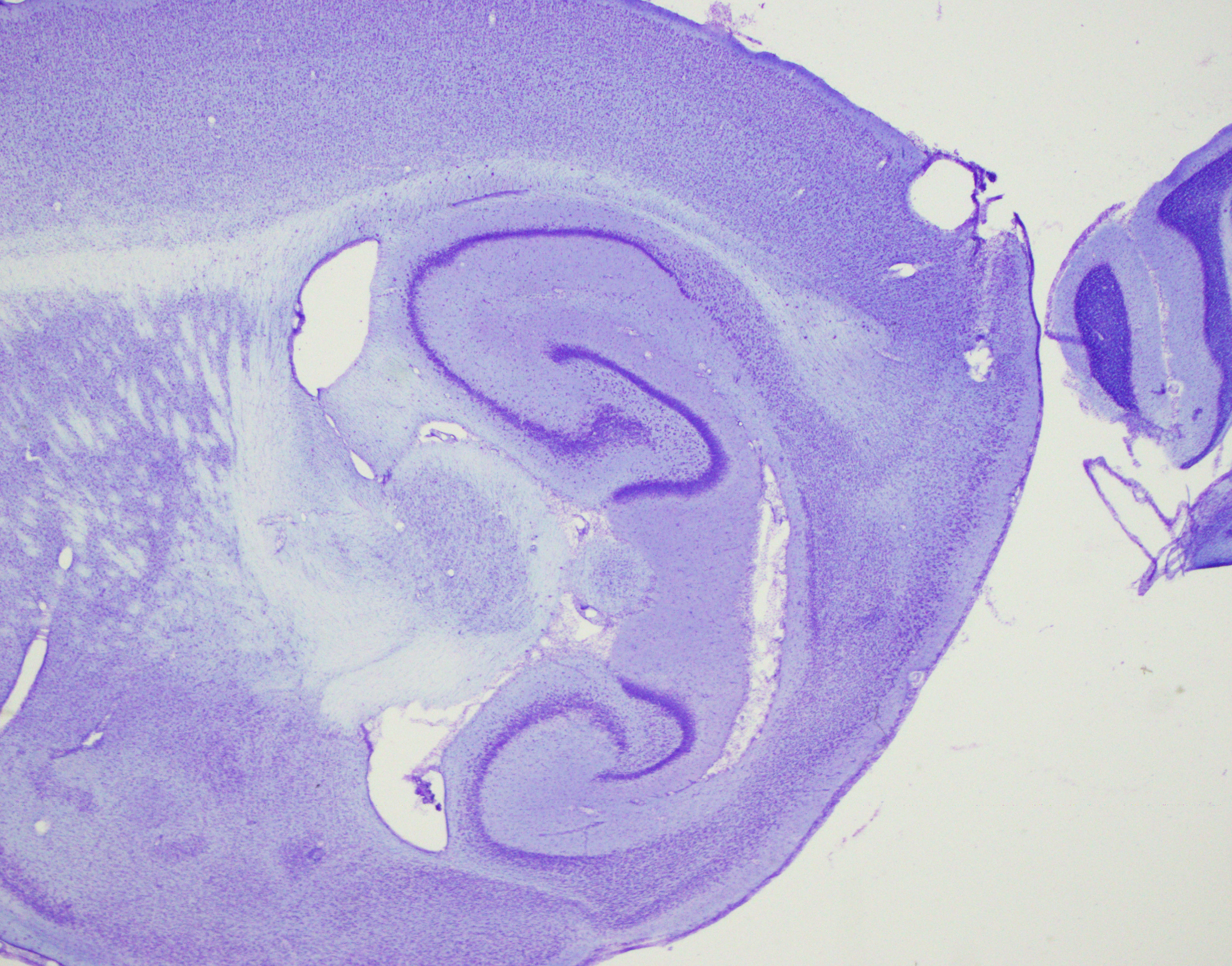

Supplement: Source Data Fig. 1 — Unprocessed histology images. [file 41593_2021_907_MOESM4_ESM.zip › 922P15 2.TIF]

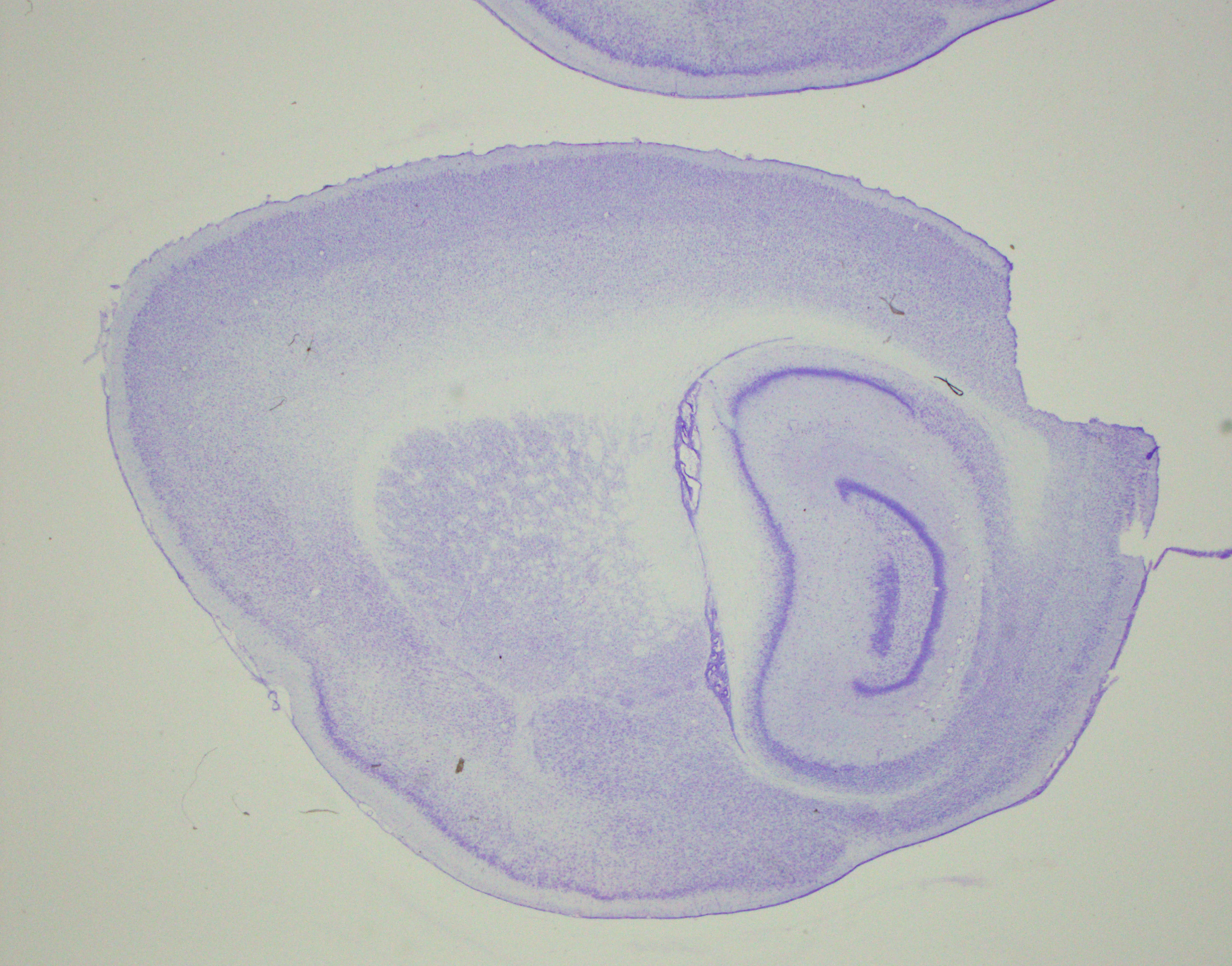

Supplement: Source Data Fig. 1 — Unprocessed histology images. [file 41593_2021_907_MOESM4_ESM.zip › FEZ 1.TIF]

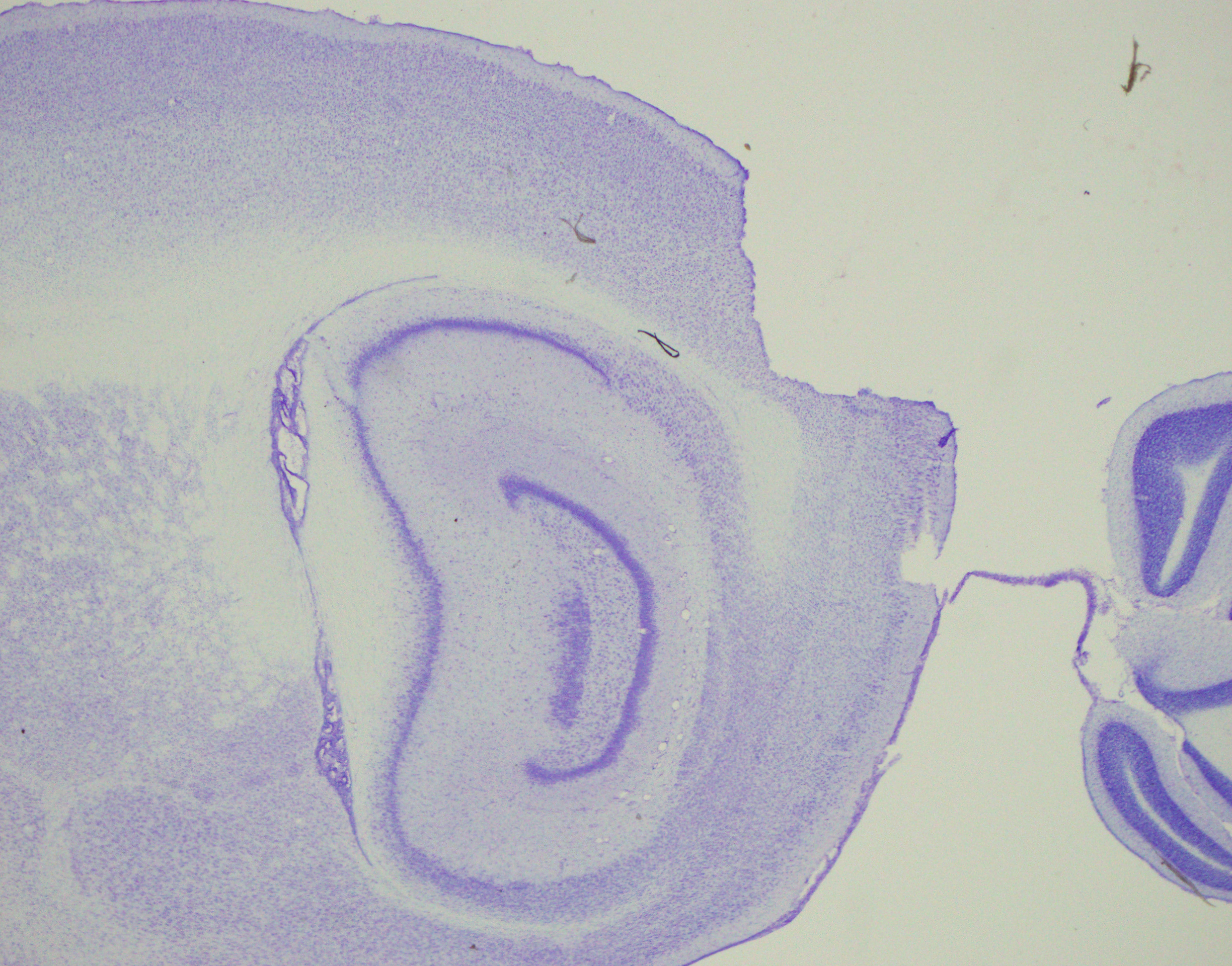

Supplement: Source Data Fig. 1 — Unprocessed histology images. [file 41593_2021_907_MOESM4_ESM.zip › FEZ 2.TIF]

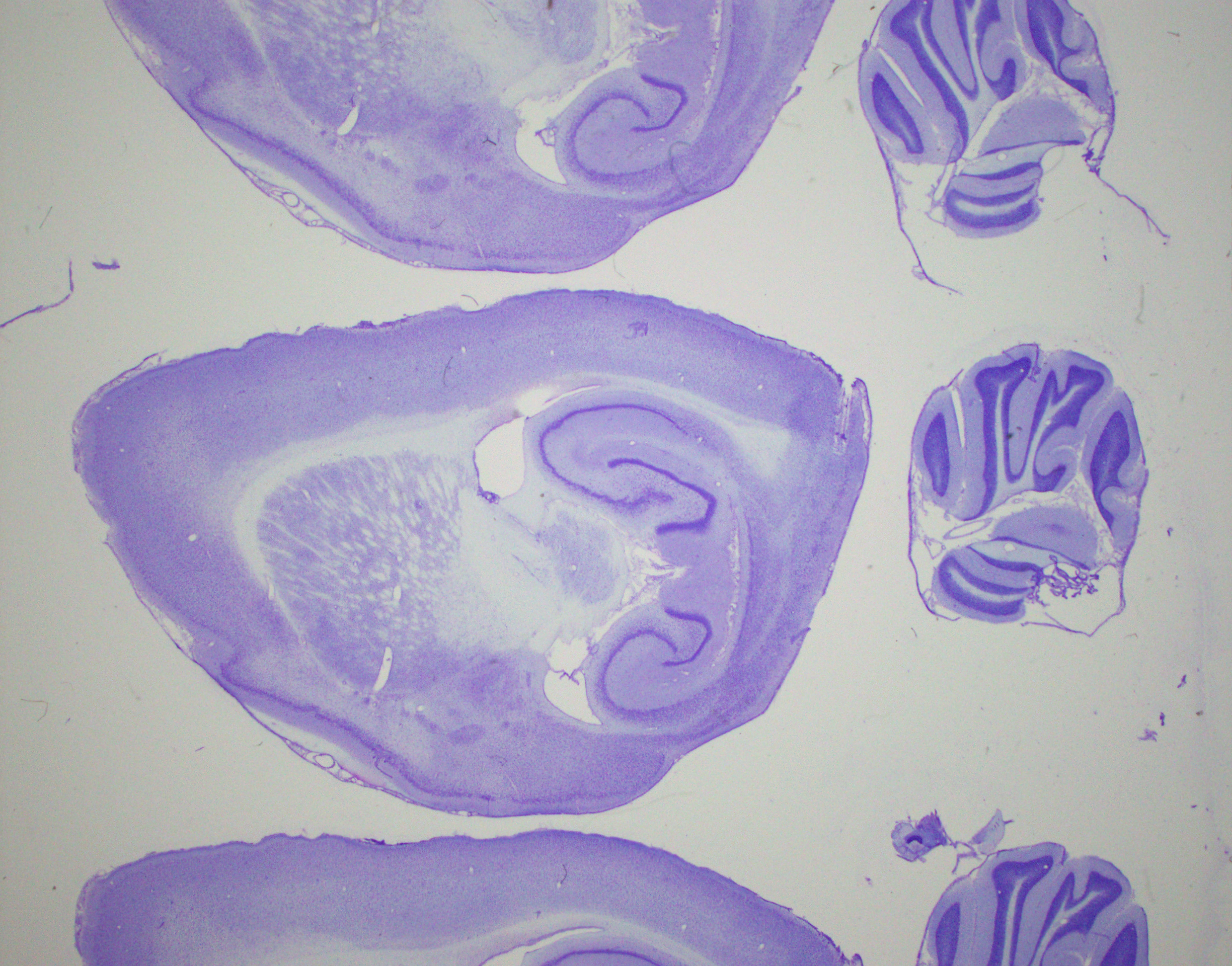

Supplement: Source Data Fig. 1 — Unprocessed histology images. [file 41593_2021_907_MOESM4_ESM.zip › ONE 1.TIF]

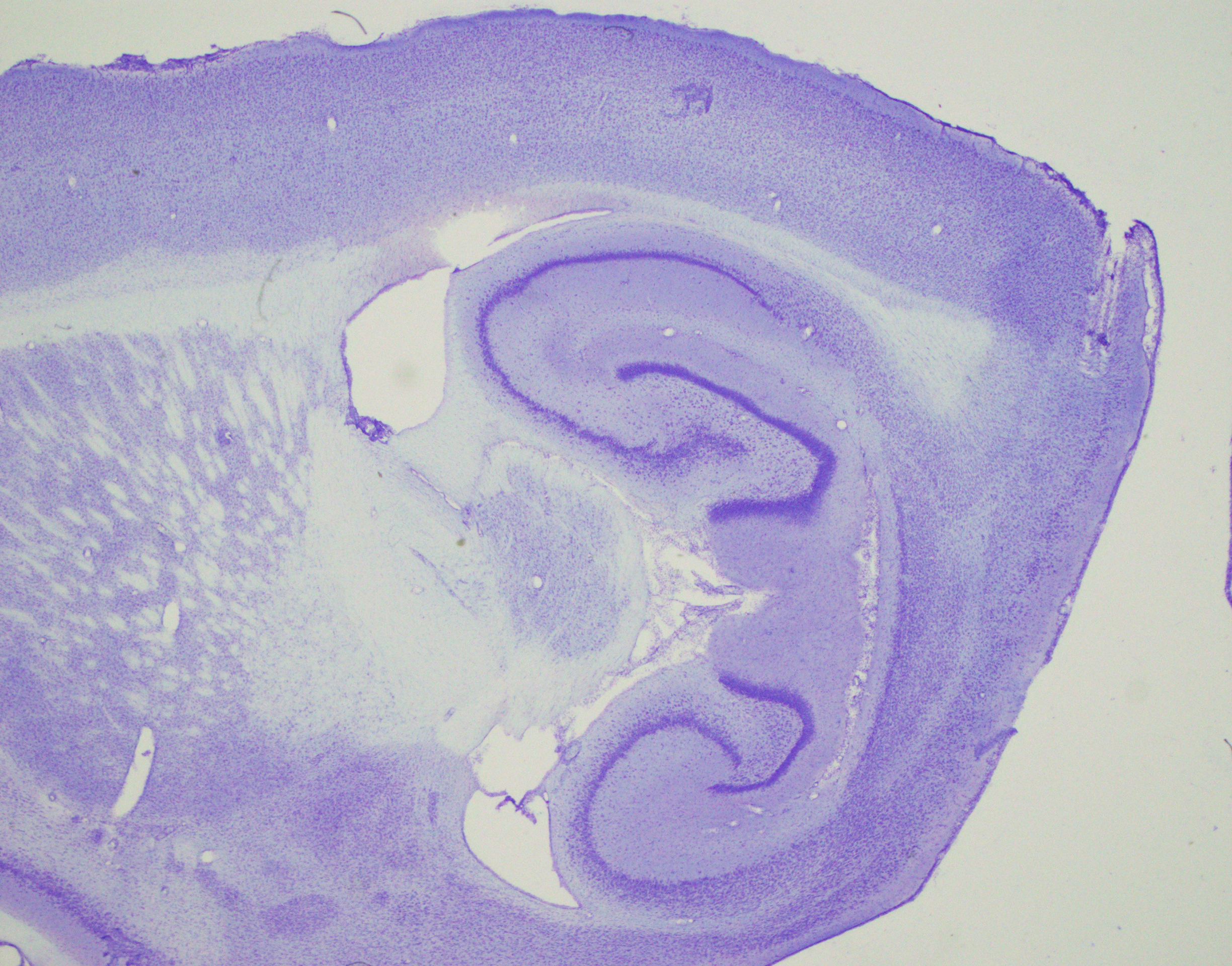

Supplement: Source Data Fig. 1 — Unprocessed histology images. [file 41593_2021_907_MOESM4_ESM.zip › ONE 2.TIF]

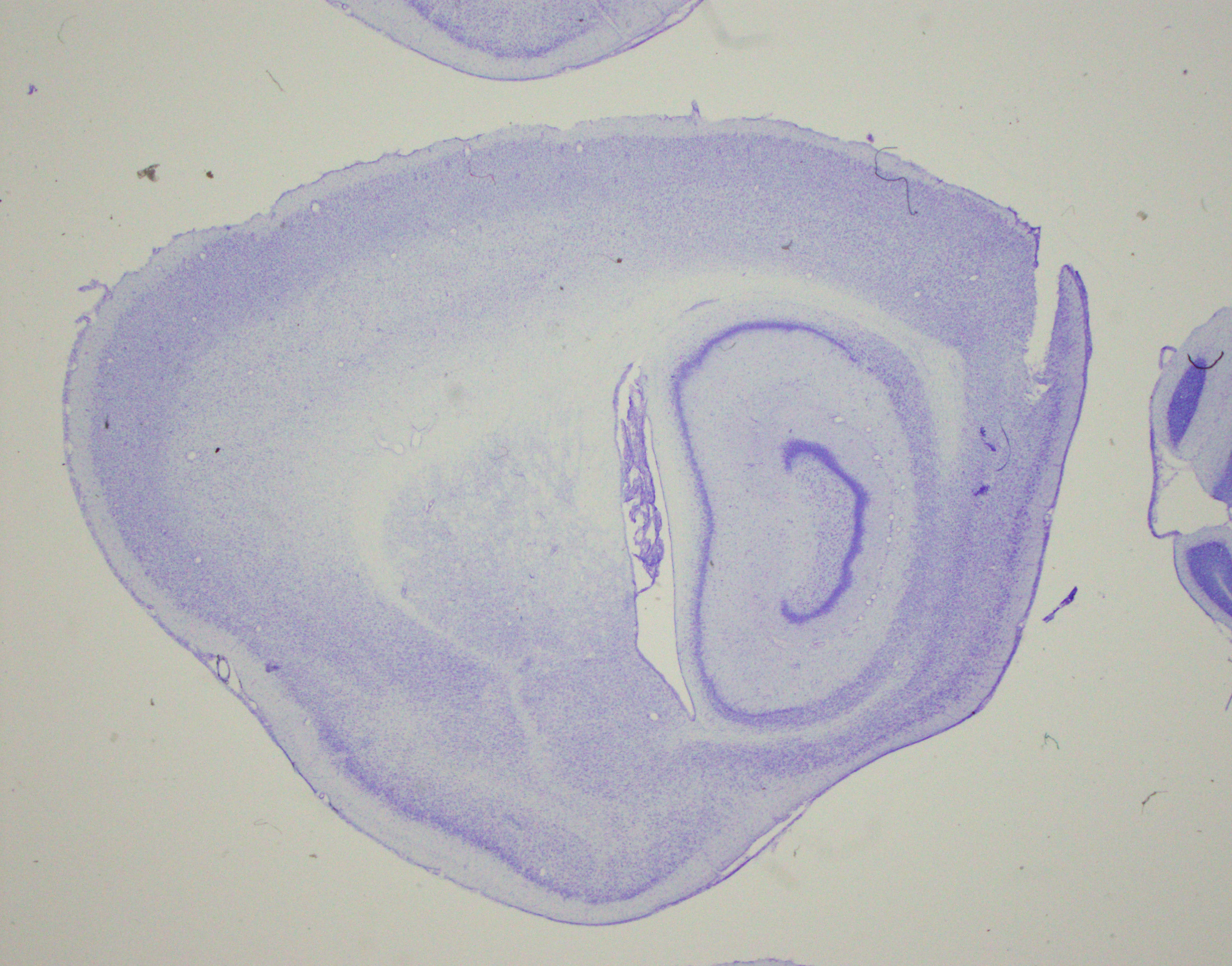

Supplement: Source Data Fig. 1 — Unprocessed histology images. [file 41593_2021_907_MOESM4_ESM.zip › SNO 1.TIF]

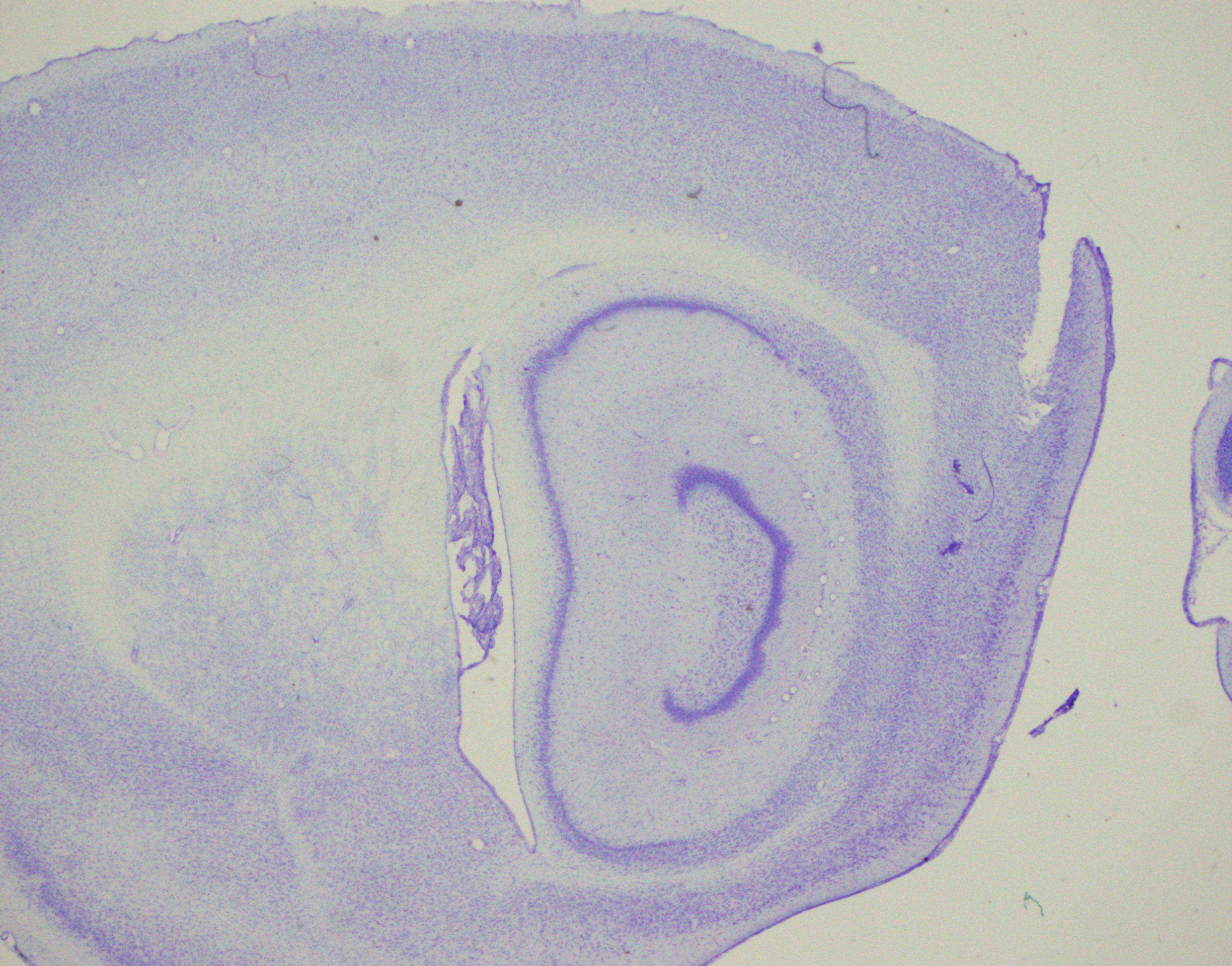

Supplement: Source Data Fig. 1 — Unprocessed histology images. [file 41593_2021_907_MOESM4_ESM.zip › SNO 2.TIF]

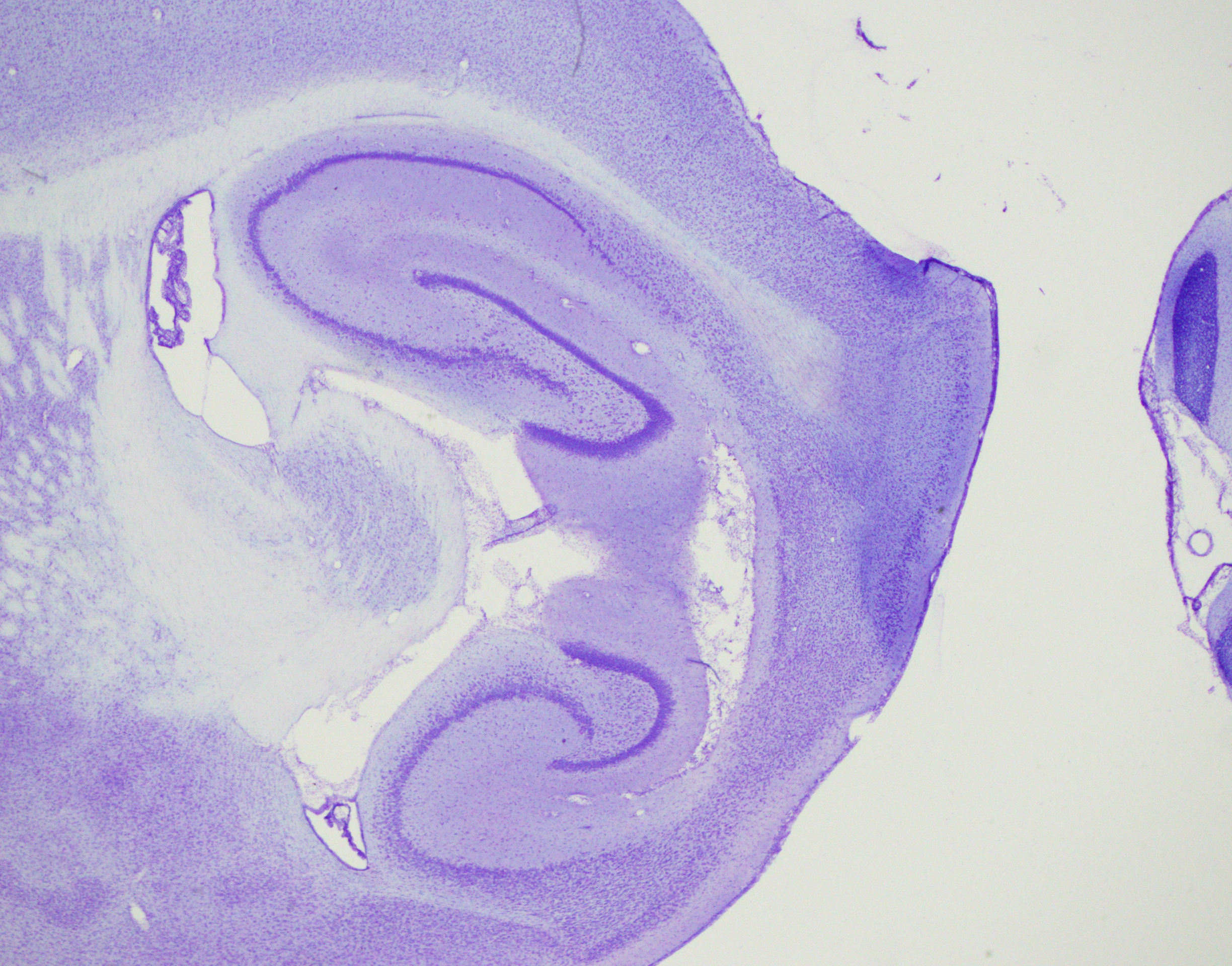

Supplement: Source Data Fig. 1 — Unprocessed histology images. [file 41593_2021_907_MOESM4_ESM.zip › ZAX 1.TIF]

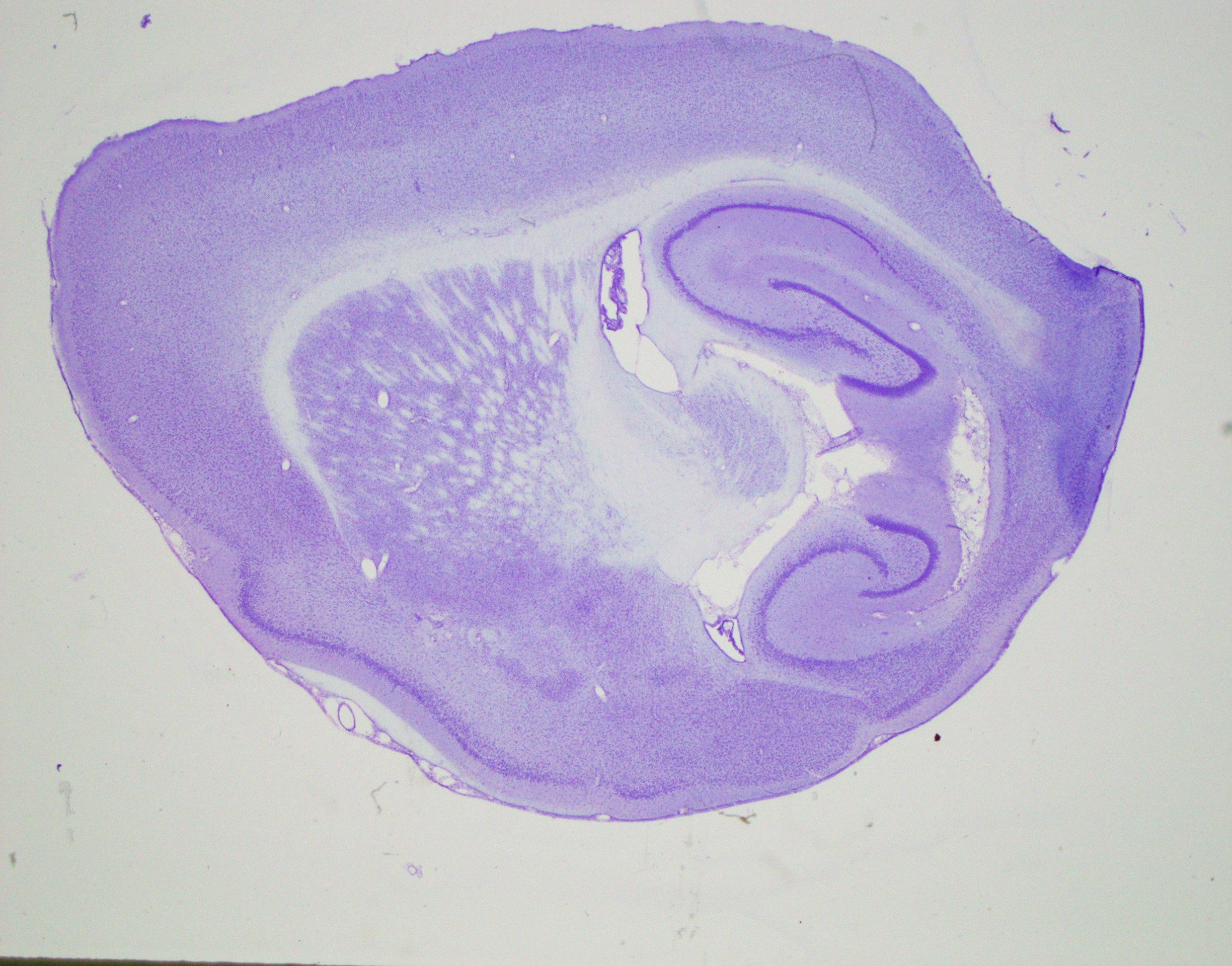

Supplement: Source Data Fig. 1 — Unprocessed histology images. [file 41593_2021_907_MOESM4_ESM.zip › ZAX 2.TIF]
